# Supplementary material for: New Diterpenes with a Fused 6-5-6-6 Ring System Isolated from the Marine Sponge-Derived Fungus Trichoderma harzianum
Source: Mar Drugs. 2019 Aug 19;17(8):480. doi: 10.3390/md17080480 (PMC6722853; doi:10.3390/md17080480)
Supplement: Supplementary file 1 [file marinedrugs-17-00480-s001.zip › Supplementary files/SM-555194-vol.1.pdf]

Supplementary Materials

New diterpenes with a fused 6-5-6-6 ring system isolated from the metabolites of a marine sponge-derived fungus *Trichoderma harzianum*

Takeshi Yamada[1,*], Ayano Fujii[1] and Takashi Kikuchi[1]

The Table of Contents

**Table S1**	**Spectral data including 2D NMR data for 1**	**2**
**Table S2**	**Spectral data including 2D NMR data for 2**	**3**
**Table S3**	**Spectral data including 2D NMR data for 3**	**4**
**Table S4**	**Spectral data including 2D NMR data for 4**	**5**
**Table S5**	**Spectral data for 2a and 2b**	**6**
**Figure S1**	**^{1}H NMR spectrum of 1 in $CDCl_3$**	**7**
**Figure S2**	**^{13}C NMR spectrum of 1 in $CDCl_3$**	**12**
**Figure S3**	**^{1}H-^{1}H COSY of 1**	**16**
**Figure S4**	**NOESY of 1**	**18**
**Figure S5**	**HMQC of 1**	**20**
**Figure S6**	**HMBC of 1**	**22**
**Figure S7**	**FABMS of 1**	**24**
**Figure S8**	**^{1}H NMR spectrum of 2 in $CDCl_3$**	**26**
**Figure S9**	**^{13}C NMR spectrum of 2 in $CDCl_3$**	**30**
**Figure S10**	**^{1}H-^{1}H COSY of 2**	**34**
**Figure S11**	**NOESY of 2**	**36**
**Figure S12**	**HMQC of 2**	**38**
**Figure S13**	**HMBC of 2**	**40**
**Figure S14**	**FABMS of 2**	**42**
**Figure S15**	**^{1}H NMR spectrum of 3 in $CDCl_3$**	**44**
**Figure S16**	**^{13}C NMR spectrum of 3 in $CDCl_3$**	**48**
**Figure S17**	**^{1}H-^{1}H COSY of 3**	**52**
**Figure S18**	**NOESY of 3**	**54**
**Figure S19**	**HMQC of 3**	**56**

**Figure S20**	**HMBC of 3**	**58**
**Figure S21**	**FABMS of 3**	**60**
**Figure S22**	**^{1}H NMR spectrum of 4 in $CDCl_3$**	**62**
**Figure S23**	**^{13}C NMR spectrum of 4 in $CDCl_3$**	**67**
**Figure S24**	**^{1}H-^{1}H COSY of 4**	**71**
**Figure S25**	**NOESY of 4**	**73**
**Figure S26**	**HMQC of 4**	**75**
**Figure S27**	**HMBC of 4**	**77**
**Figure S28**	**FABMS of 4**	**80**
**Figure S29**	**^{1}H NMR spectrum of 2a in $CDCl_3$**	**82**
**Figure S30**	**^{1}H-^{1}H COSY of 2a**	**87**
**Figure S31**	**NOESY of 2a**	**89**
**Figure S32**	**FABMS of 2a**	**91**
**Figure S33**	**^{1}H NMR spectrum of 2b in $CDCl_3$**	**93**
**Figure S34**	**^{1}H-^{1}H COSY of 2b**	**98**
**Figure S35**	**NOESY of 2b**	**100**
**Figure S36**	**FABMS of 2b**	**102**

Table S1 NMR spectral data of **1** in $CDCl_3$

Position	δ_H[a]		J/Hz	^1H-^1H COSY	NOESY[b]	δ_C		HMBC (C)[c]
1	4.11	d	5.4 (2)	2	7β, 20	80.4	(d)	2, 5, 6, 7
2	3.88	dd	7.8 (3), 5.4 (1)	1, 3	17, 19	83.7	(d)	1, 3, 17
3	1.88	qd	7.8 (17), 7.8 (3)	2α, 2β, 17	11, 20	36.6	(d)	2, 4, 5, 12, 17
4						41.2	(s)	
5						39.4	(s)	
6	1.50	dd	4.8 (7α), 3.0 (7β)	7α, 7β	18, 19	53.2	(d)	4
7α	1.78	dd	13.8 (7β), 4.8 (6)	6, 7β	9α, 12, 18	40.9	(t)	1, 6, 8, 9, 20
7β	1.70	dd	13.8 (7α), 3.0 (6)	6, 7α	1, 20			5, 12
8						39.6	(s)	
9α	1.03	m		9β, 10α, 10β	7α, 12	43.5	(t)	
9β	1.43	m		9α, 10α, 10β	20			20
10α	1.59	m		9α, 9β, 10β, 11	16	21.6	(t)	8, 15
10β	1.80	m		9α, 9β, 10α, 11				
11	1.81	dd	13.2 (12), 4.2 (10)	10α, 10β, 12	3, 14β, 20	44.2	(d)	10, 12, 15, 16
12	1.32	d	13.2 (11)	11	7α, 9α, 16, 18	51.8	(d)	3, 4, 5, 8, 11, 15, 20
13α	1.23	ddd	13.8 (13β), 13.8 (14β), 3.6 (14α)	13β, 14α, 14β	18	26.3	(t)	
13β	1.72	ddd	13.8 (13α), 3.6 (14β), 3.6 (14α)	13α, 14α, 14β	17, 19			5, 12
14α	1.64	ddd	13.8 (12β), 3.6 (13α), 3.6 (13β)	13α, 13β, 14β	16	41.1	(t)	
14β	1.46	ddd	13.8 (12α), 13.8 (13α), 3.6 (13β)	13α, 13β, 14α	11, 17			11
15						73.6	(s)	
16	1.18	s			10α, 12, 14α	20.5	(q)	11, 14, 15
17	1.23	d	7.2 (3)	3	2, 13β, 14β, 19	20.0	(q)	2, 3, 4
18ax	0.99	s			6, 7α, 12, 13α	25.7	(q)	4, 5, 6, 19
19eq	1.04	s			2, 6, 13β, 17	25.2	(q)	4, 5, 6, 18
20	0.98	s			1, 3, 7β, 9β, 11	19.8	(q)	7, 8, 9, 12

a ^1H chemical shift values (δ ppm from $SiMe_4$) followed by multiplicity and then the coupling constants (J /Hz). Figures in parentheses indicate the proton coupling with that position. b The correlations with geminal and vicinal protones are removed. c Long range ^1H-^{13}C correlations from H to C observed in the HMBC experiment.

Table S2 NMR spectral data of **2** in $CDCl_3$

Position	δ_H[a]		J/Hz	^{1}H-^{1}H COSY	NOESY[b]	δ_C	HMBC (C)[c]
1α	2.54	ddd	15.0 (1α), 11.4 (2), 11.4 (6)	2, 6	19	36.5 (t)	2, 3, 6, 7
1β	1.68	m		2, 6	20		2, 5, 6, 7
2	4.30	ddd	11.4 (1β), 7.2 (3), 6.6 (1α)	1α, 2α, 3	17, 19	74.2 (d)	1, 3, 17
3	1.98	qd	7.2 (17), 7.2 (2)	2, 17	11, 14β, 20	37.9 (d)	2, 4, 5, 12, 17
4						40.8 (s)	
5						39.0 (s)	
6	1.62	m		1α, 1β, 7α, 7β	18, 19	41.8 (d)	
7α	1.71	dd	13.2 (7β), 4.2 (6)	6, 7β	9α, 12, 18	42.5 (t)	1, 6, 8, 9
7β	1.56	dd	13.2 (7α), 3.0 (6)	6, 7α	20		5, 8, 12, 20
8						39.7 (s)	
9α	1.02	m		9β, 10α, 10β	7α, 12	43.9 (t)	
9β	1.43	m		9α, 10α, 10β	20		11, 12, 20
10α	1.60	m		9α, 9β, 10β, 11	12, 16	21.6 (t)	11, 15
10β	1.81	m		9α, 9β, 10α, 11			9
11	1.85	m		10α, 10β, 12	3, 20	44.4 (d)	10, 12, 15, 16
12	1.30	d	13.2 (11)	11	7α, 9α, 10α, 16, 18	51.9 (d)	3, 4, 5, 8, 9, 10, 11, 15, 20
13α	1.18	ddd	13.8 (13β), 13.8 (14β), 3.6 (14α)	13β, 14α, 14β	18, 19	26.4 (t)	12
13β	1.68	m		13α, 14α, 14β	17		
14α	1.62	m		13α, 13β, 14β		41.2 (t)	
14β	1.46	ddd	13.8 (12α), 13.8 (13α), 3.0 (13β)	13α, 13β, 14α	3, 17		
15						73.8 (s)	
16	1.18	s			10α, 12	20.4 (q)	11, 14, 15
17	1.19	d	7.8 (3)	3	2, 13β, 14β, 19	20.4 (q)	2, 3, 4
18ax	0.95	s			6, 7α, 12, 13α	25.3 (q)	4, 5, 6, 19
19eq	0.96	s			2, 6, 13α, 17	25.4 (q)	4, 5, 6, 18
20	1.13	s			1β, 3, 7β, 9β, 11	20.3 (q)	7, 8, 9, 12

a ^{1}H chemical shift values (δ ppm from $SiMe_4$) followed by multiplicity and then the coupling constants (*J* /Hz). Figures in parentheses indicate the proton coupling with that position. b The correlations with geminal and vicinal protones are removed. c Long range ^{1}H-^{13}C correlations from H to C observed in the HMBC experiment.

Table S3 NMR spectral data of **3** in $CDCl_3$

Position	δ_H[a]		J/Hz	^1H-^1H COSY	NOESY[b]	δ_C		HMBC (C)[c]
1	4.20	dd	9.6 (2α), 5.4 (2β)	2α, 2β, 6	7β, 20	72.6	(d)	5, 7
2α	2.66	ddd	16.2 (2β), 9.6 (1), 9.6 (3)	1, 2β, 3	17, 19	41.8	(t)	1, 3, 17
2β	1.45	m		1, 2α, 3	20			1, 3, 4, 6
3	2.10	m		2α, 2β, 17	11, 20	26.7	(d)	2, 4, 5, 12, 17
4						38.9	(s)	
5						39.0	(s)	
6	1.41	dd	4.2 (7α), 3.0 (7β)	1, 7α, 7β	18	52.2	(d)	
7α	1.69	dd	13.8 (7β), 4.2 (6)	6	12, 18	41.3	(t)	1, 5, 6, 20
7β	1.65	dd	13.8 (7α), 3.0 (6)	6	1, 20			1, 5, 12, 20
8						38.8	(s)	
9	1.48	m		10	20	52.2	(t)	8, 10, 11, 12, 20
10	4.38	ddd	7.8 (9), 4.8 (11), 1.2 (9)	9, 11	12, 16	72.9	(d)	8, 15
11	1.85	dd	13.8 (12), 4.8 (10)	10, 12	3, 20	55.0	(d)	10, 12, 15, 16
12	1.28	d	13.8 (11)	11	7α, 10, 18	50.6	(d)	3, 4, 5, 8, 9, 10, 11, 15, 20
13α	1.19	ddd	14.0 (13β), 14.0 (14β), 3.0 (14α)	13β, 14α, 14β	18	25.9	(t)	4, 12, 14
13β	1.74	ddd	14.0 (13α), 3.0 (14β), 3.0 (14α)	13α, 14α, 14β	17, 19			
14α	1.60	ddd	14.0 (12β), 3.0 (13α), 3.0 (13β)	13α, 13β, 14β	16	41.0	(t)	13
14β	1.50	ddd	14.0 (12α),14.0 (13α), 3.0 (13β)	13α, 13β, 14α				4, 15, 16
15						73.1	(s)	
16	1.22	s			10, 14α	21.5	(q)	11, 14, 15
17	1.09	d	6.6 (3)	3	2α, 13β	22.4	(q)	2, 3, 4
18ax	0.97	s			6, 7α, 12, 13α	25.8	(q)	4, 5, 6, 19
19eq	1.14	s			2α, 13β	24.9	(q)	4, 5, 6, 18
20	1.17	s			1, 2β, 3, 7β, 9, 11	21.4	(q)	7, 8, 9, 12

a ^1H chemical shift values (δ ppm from $SiMe_4$) followed by multiplicity and then the coupling constants (*J* /Hz). Figures in parentheses indicate the proton coupling with that position. b The correlations with geminal and vicinal protones are removed. c Long range ^1H-^{13}C correlations from H to C observed in the HMBC experiment.

Table S4 NMR spectral data of **4** in $CDCl_3$

Position	δ_H[a]		J/Hz	^1H-^1H COSY	NOESY[b]	δ_C		HMBC (C)[c]
1	4.24	dd	10.2 (2α), 6.0 (2β)	2α, 2β, 17	7β, 20	72.1	(d)	5, 7
2α	2.74	ddd	16.2 (2β), 10.2 (1), 10.2 (3)	1, 2β, 3	19	36.9	(t)	1, 3, 4, 6
2β	1.64	ddd	16.2 (2α), 6.0 (1), 3.6 (3)	1, 2α, 3				17
3	1.88	dddd	10.2 (2α), 10,2 (17A), 3.6 (2β), 3.6 (17B)	2α, 2β, 17A	11, 20	34.0	(d)	
4						38.9	(s)	
5						38.5	(s)	
6	1.45	dd	4.8 (7α), 3.6 (7β)	7α, 7β	18	52.2	(d)	
7α	1.74	dd	13.8 (7β), 4.8 (6)	6, 7β	9α, 18	40.9	(t)	1, 6, 8, 9, 20
7β	1.68	dd	13.8 (7α), 3.6 (6)	6, 7α	1, 20			5, 12
8						39.2	(s)	
9α	1.03	m		10α	7α	43.5	(t)	
9β	1.43	m		10β	20			8, 11
10α	1.59	m		9α, 11	16	21.5	(t)	
10β	1.80	m		9β	20			
11	1.88	ddd	13.2 (12), 10.2 (10), 6.0 (10)	10α, 12	3, 14β, 20	44.1	(d)	12, 15
12	1.28	d	13.2 (11)	11	16, 18	52.0	(d)	3, 4, 5, 8, 9, 11, 15, 20
13α	1.28	m		13β, 14α, 14β		25.5	(t)	
13β	1.52	m		13α, 14α, 14β	17, 19			
14α	1.67	m		13α, 13β, 14β		40.87	(t)	4
14β	1.50	m		13α, 13β, 14α	11			15, 16
15						73.6	(s)	
16	1.18	s			10α, 12	20.5	(q)	11, 14, 15
17A	3.60	dd	10.2 (3), 10.2 (17B)	3	13β, 19	68.1	(t)	2, 3
17B	3.95	dd	10.2 (17A), 3.6 (3)		13β			2
18ax	0.99	s			6, 7α, 12	25.7	(q)	4, 5, 6, 19
19eq	1.13	s			2α, 13β, 17A	25.5	(q)	4, 5, 6, 18
20	0.92	s			1, 3, 7β, 9β, 10β, 11	19.5	(q)	7, 8, 9, 12

a ^{1}H chemical shift values (δ ppm from $SiMe_4$) followed by multiplicity and then the coupling constants (*J* /Hz). Figures in parentheses indicate the proton coupling with that position. b The correlations with geminal and vicinal protones are removed. c Long range ^{1}H-^{13}C correlations from H to C observed in the HMBC experiment.

Table S5 NMR spectral data of MTPA esters **2a** and **2b** in $CDCl_3$

Position	δ_H[a]		*J*/Hz	δ_H[a]		*J*/Hz
	**2a**			**2b**		
1α	2.82	ddd	16.2 (1α), 10.8 (2), 10.8 (6)	2.85	ddd	15.0 (1α), 11.4 (2), 11.4 (6)
1β	1.52	m		1.66	m	
2	5.25	ddd	11.4 (1β), 6.6 (3), 6.6 (1α)	5.28	ddd	11.4 (1β), 7.2 (3), 6.6 (1α)
3	2.21	qd	7.2 (17), 7.2 (2)	2.17	qd	7.2 (17), 7.2 (2)
4						
5						
6	1.64	m		1.64	m	
7α	1.68	m		1.72	dd	16.2 (7β), 4.2 (6)
7β	1.46	dd	13.8 (7α), 3.0 (6)	1.53	dd	16.2 (7α), 3.0 (6)
8						
9α	1.00	m		1.02	m	
9β	1.36	m		1.42	m	
10α	1.58	m		1.60	m	
10β	1.79	m		1.81	m	
11	1.81	m		1.81	m	
12	1.28	d	12.6 (11)	1.29	d	12.6 (11)
13α	1.20	ddd	14.4 (13β), 14.4 (14β), 3.0 (14α)	1.19	m	
13β	1.70	m		1.68	m	
14α	1.62	m		1.62	m	
14β	1.42	m		1.40	m	
15						
16	1.17	s		1.17	s	
17	1.13	d	7.2 (3)	1.02	d	7.2 (3)
18ax	0.97	s		0.98	s	
19eq	1.01	s		1.01	s	
20	0.75	s		0.97	s	
OCH_3	3.57	s		3.53	s	
Ar.H	7.38-7.41	m		7.39-7.41	m	
Ar.H	7.53	m		7.53	m	

a ^1H chemical shift values (δ ppm from $SiMe_4$) followed by multiplicity and then the coupling constants (J/Hz). Figures in parentheses indicate the proton coupling with that position.

Figure S1 ^{1}H NMR spectrum of 1 in CDCl$_3$

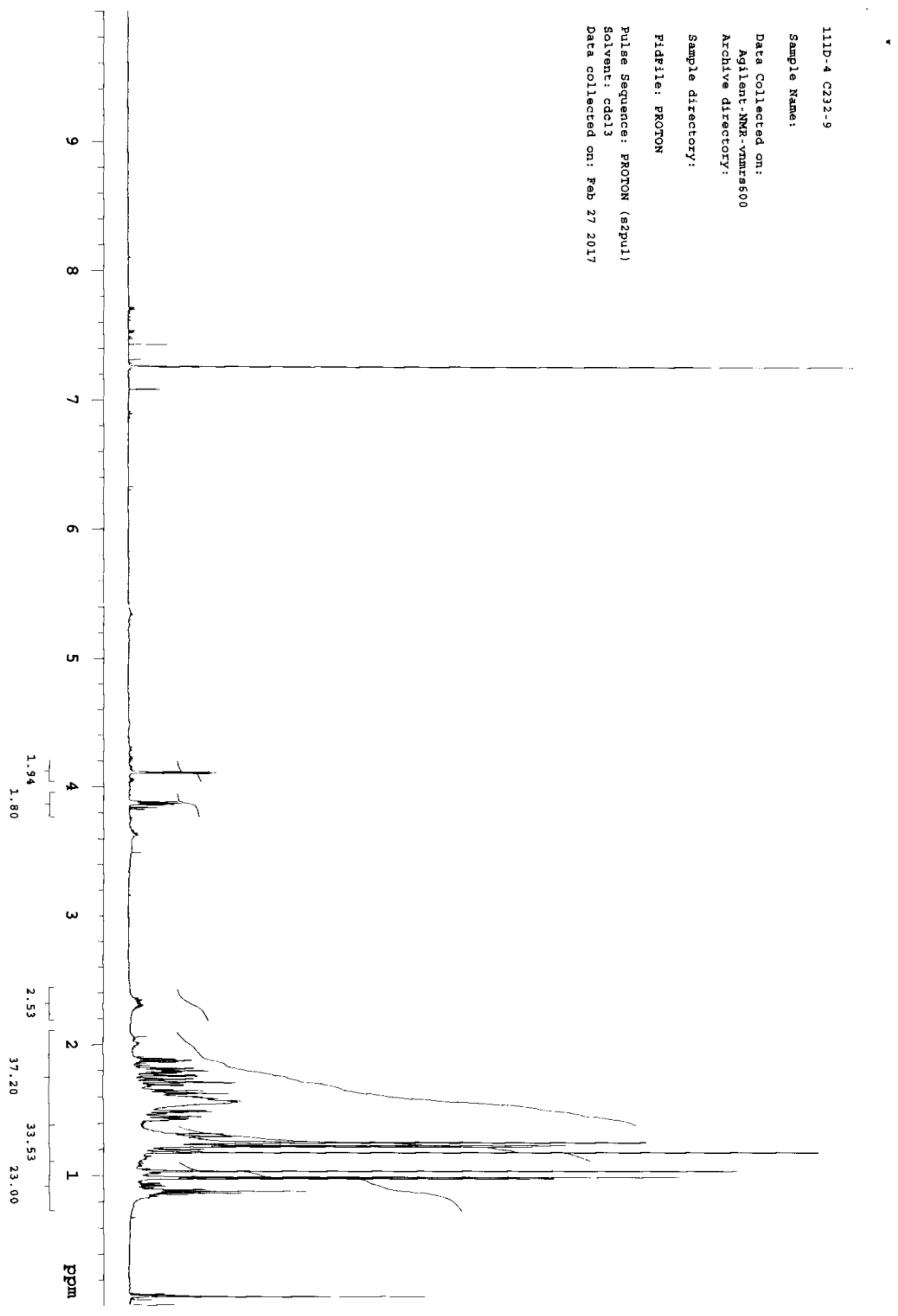

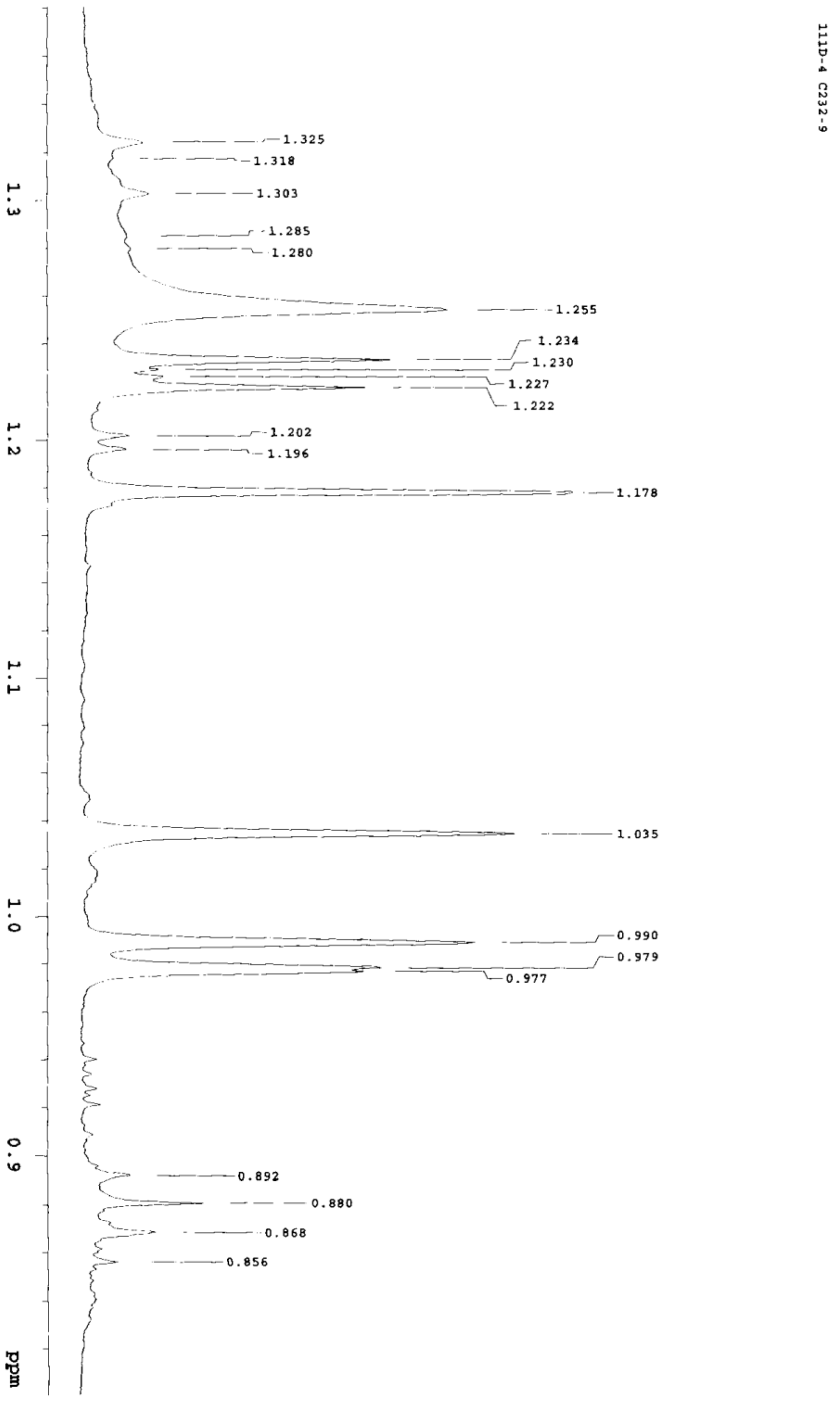
111D-4 C232-9
1.325
1.318
1.303
1.285
1.280
1.255
1.234
1.230
1.227
1.222
1.202
1.196
1.178
1.035
0.990
0.979
0.977
0.892
0.880
0.868
0.856
1.3
1.2
1.1
1.0
0.9
ppm

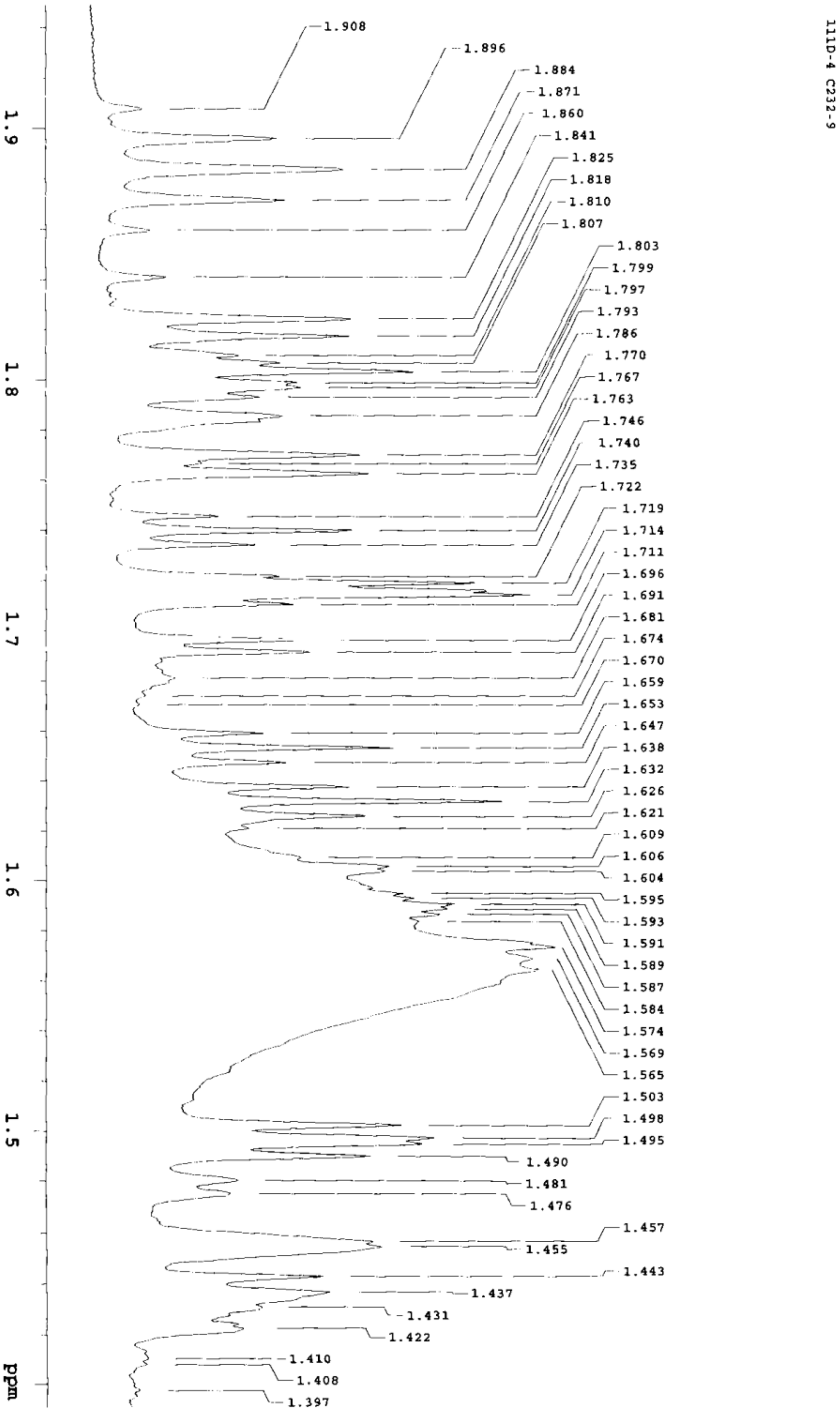
111D-4 C232-9
1.908
1.896
1.884
1.871
1.860
1.841
1.825
1.818
1.810
1.807
1.803
1.799
1.797
1.793
1.786
1.770
1.767
1.763
1.746
1.740
1.735
1.722
1.719
1.714
1.711
1.696
1.691
1.681
1.674
1.670
1.659
1.653
1.647
1.638
1.632
1.626
1.621
1.609
1.606
1.604
1.595
1.593
1.591
1.589
1.587
1.584
1.574
1.569
1.565
1.503
1.498
1.495
1.490
1.481
1.476
1.457
1.455
1.443
1.437
1.431
1.422
1.410
1.408
1.397
1.9
1.8
1.7
1.6
1.5
ppm

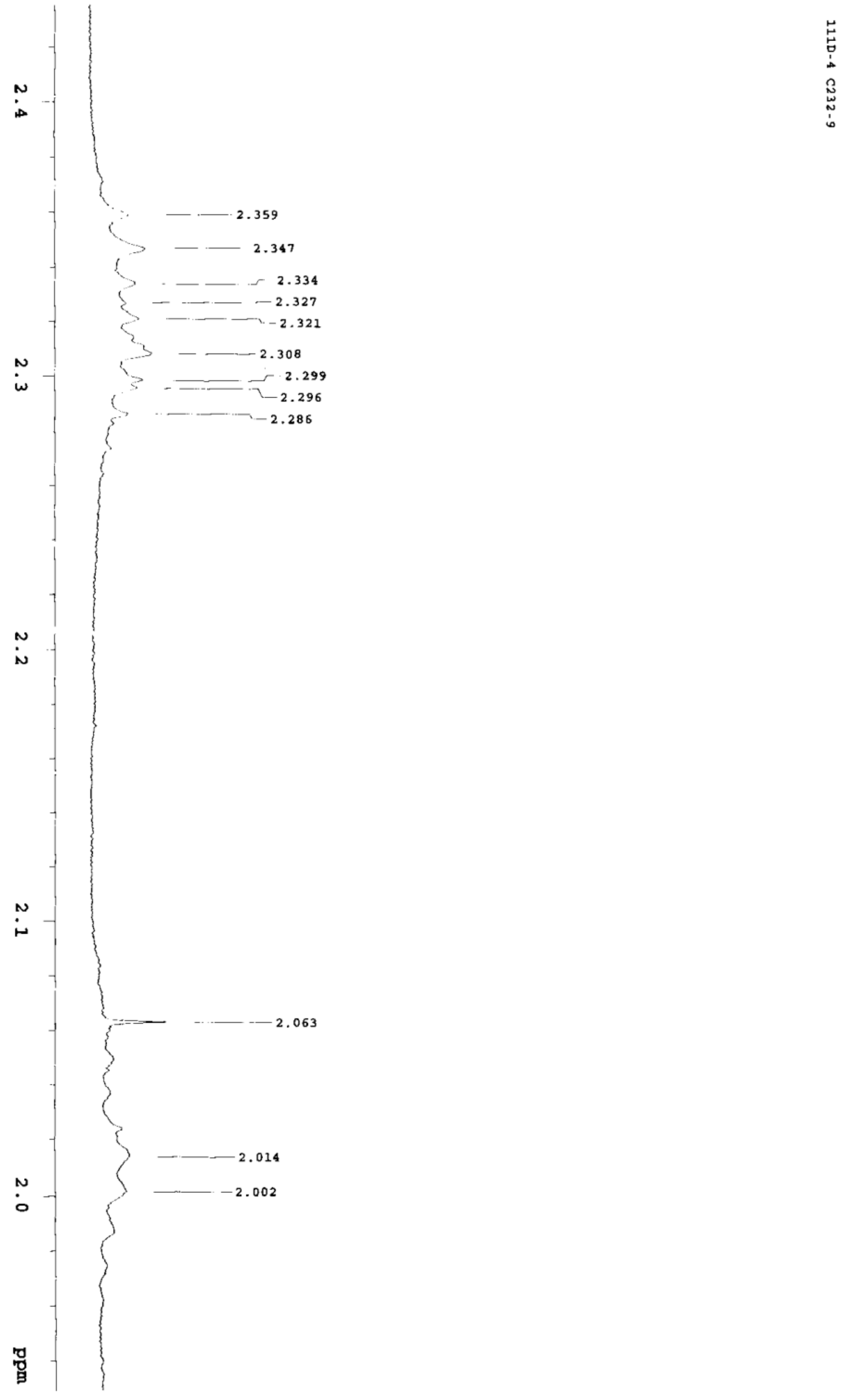
111D-4 C232-9
2.359
2.347
2.334
2.327
2.321
2.308
2.299
2.296
2.286
2.063
2.014
2.002
2.4
2.3
2.2
2.1
2.0
ppm

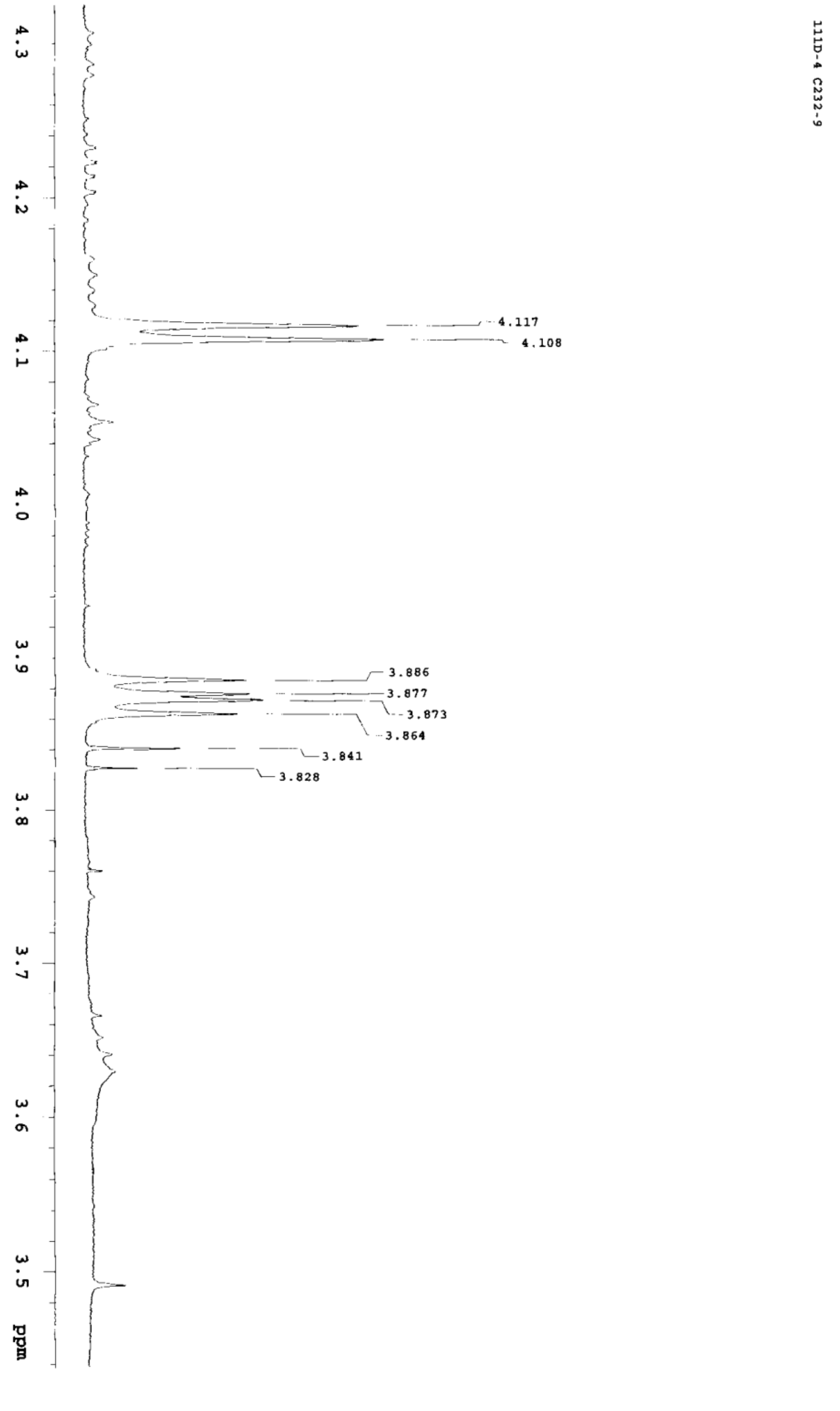
111D-4 C232-9
4.117
4.108
3.886
3.877
3.873
3.864
3.841
3.828
4.3
4.2
4.1
4.0
3.9
3.8
3.7
3.6
3.5
ppm

Figure S2 ^{13}C NMR spectrum of 1 in CDCl$_3$

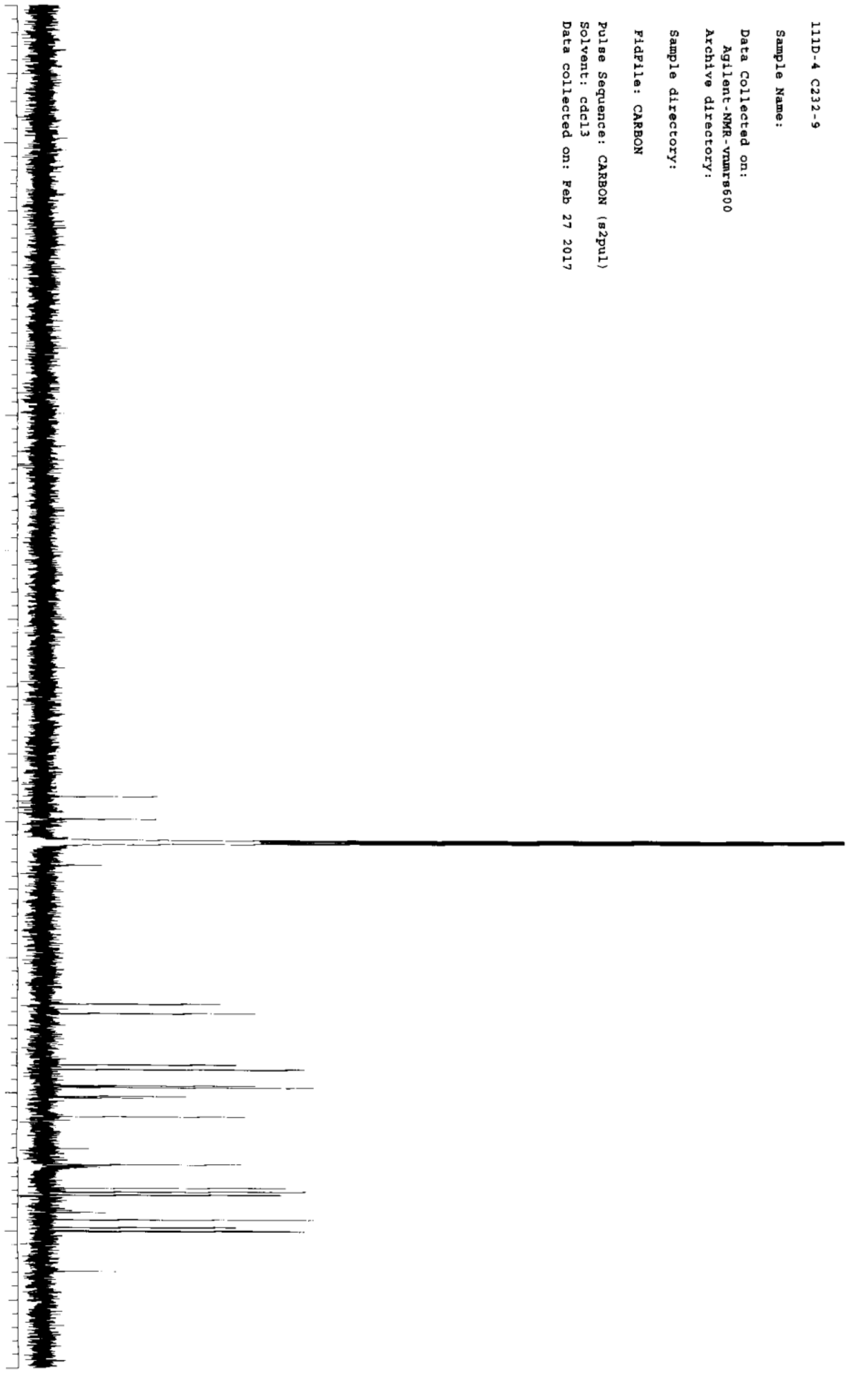

111D-4 C232-9

INDEX	FREQUENCY	PPM	HEIGHT
1	12623.0	83.682	21.9
2	12124.8	80.380	21.7
3	11647.3	77.215	2875.2
4	11615.0	77.000	3162.6
5	11582.6	76.785	2979.1
6	11104.0	73.613	11.2
7	8022.2	53.182	34.3
8	7810.7	51.780	41.4
9	6669.7	44.216	37.3
10	6564.6	43.519	50.5
11	6208.5	41.159	21.4
12	6201.6	41.113	41.0
13	6168.1	40.890	52.5
14	5973.9	39.603	27.6
15	5940.3	39.381	19.3
16	5523.0	36.614	39.1
17	4814.4	31.917	9.5
18	4479.2	29.694	38.4
19	4428.3	29.357	11.0
20	3962.5	26.269	46.9
21	3872.3	25.671	50.7
22	3804.1	25.219	46.0
23	3421.5	22.682	12.1
24	3256.2	21.586	52.5
25	3085.1	20.452	37.2
26	3015.7	19.992	47.6
27	2991.5	19.831	50.9
28	2127.9	14.107	14.0

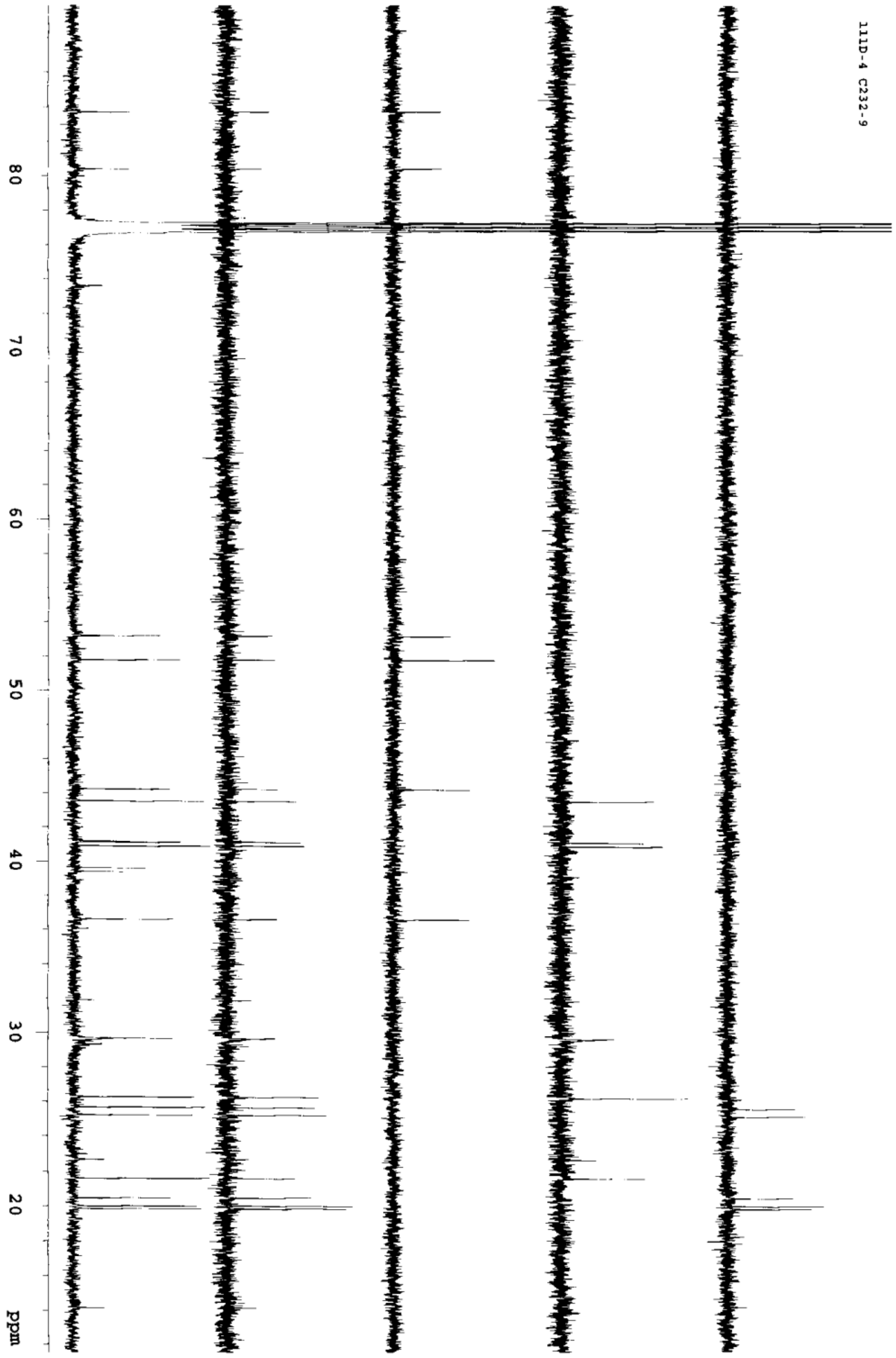
111D-4 C232-9
80
70
60
50
40
30
20
ppm

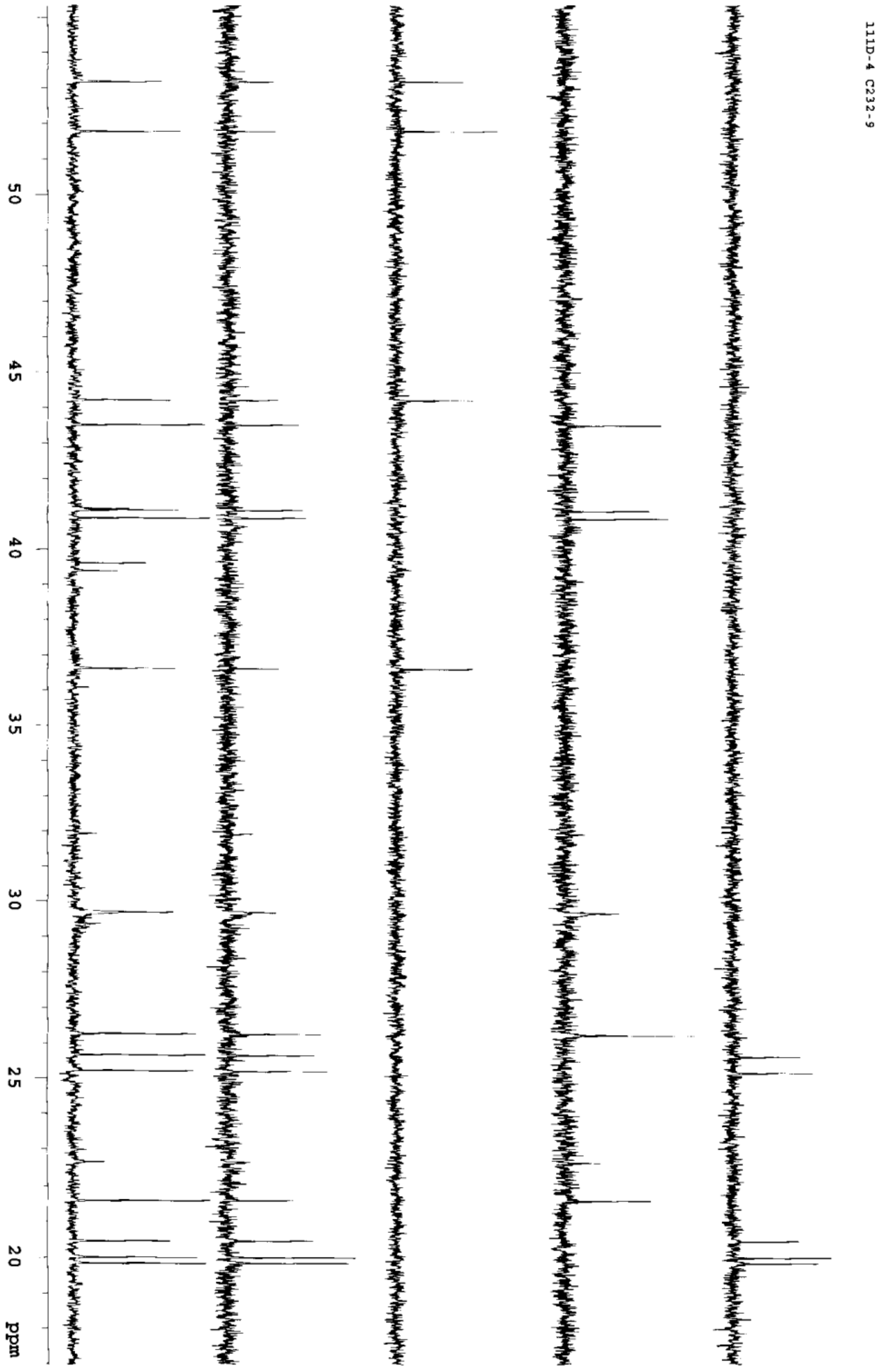
111D-4 C232-9
50
45
40
35
30
25
20
ppm

Figure S3 ^{1}H-^{1}H COSY of 1

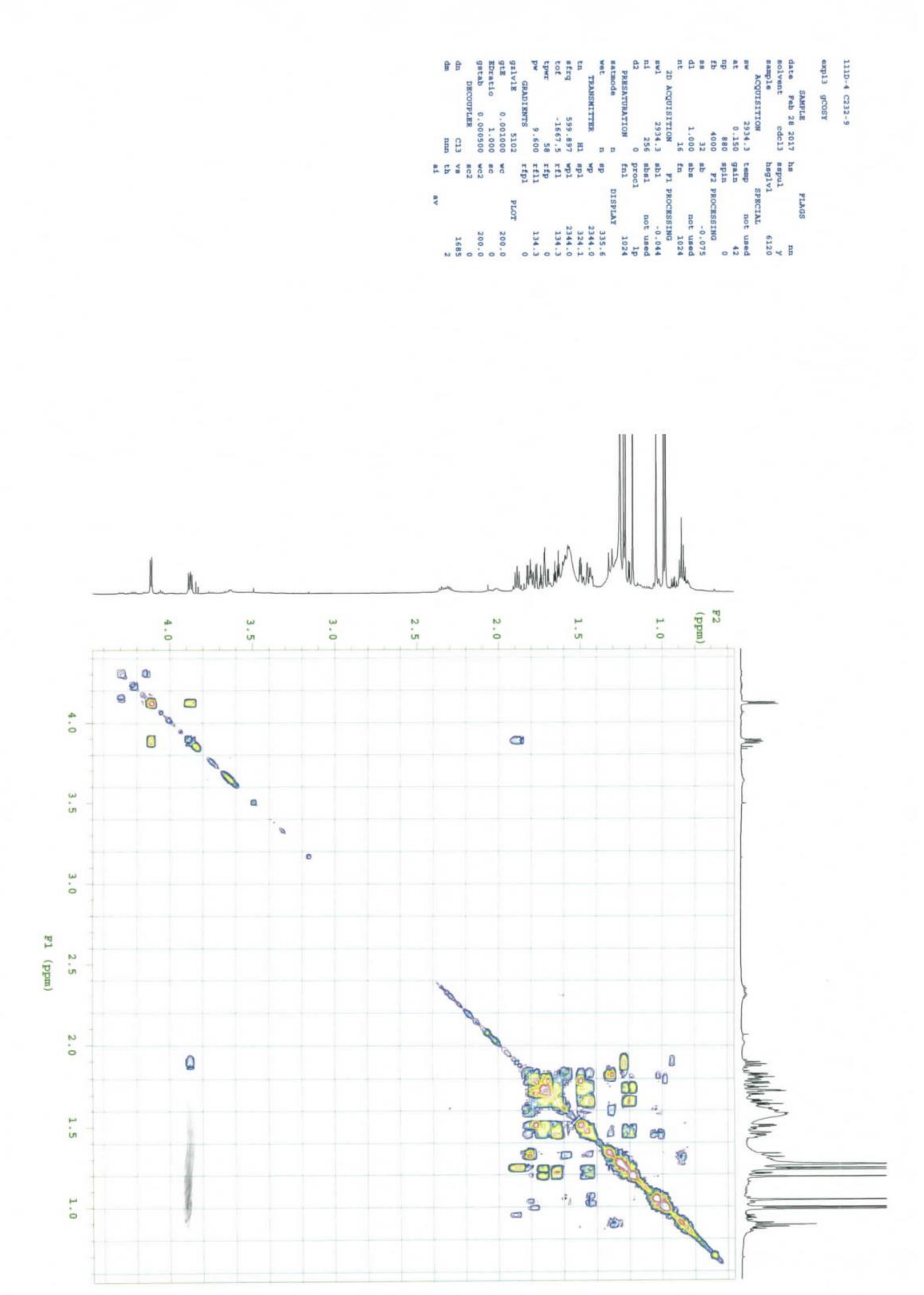

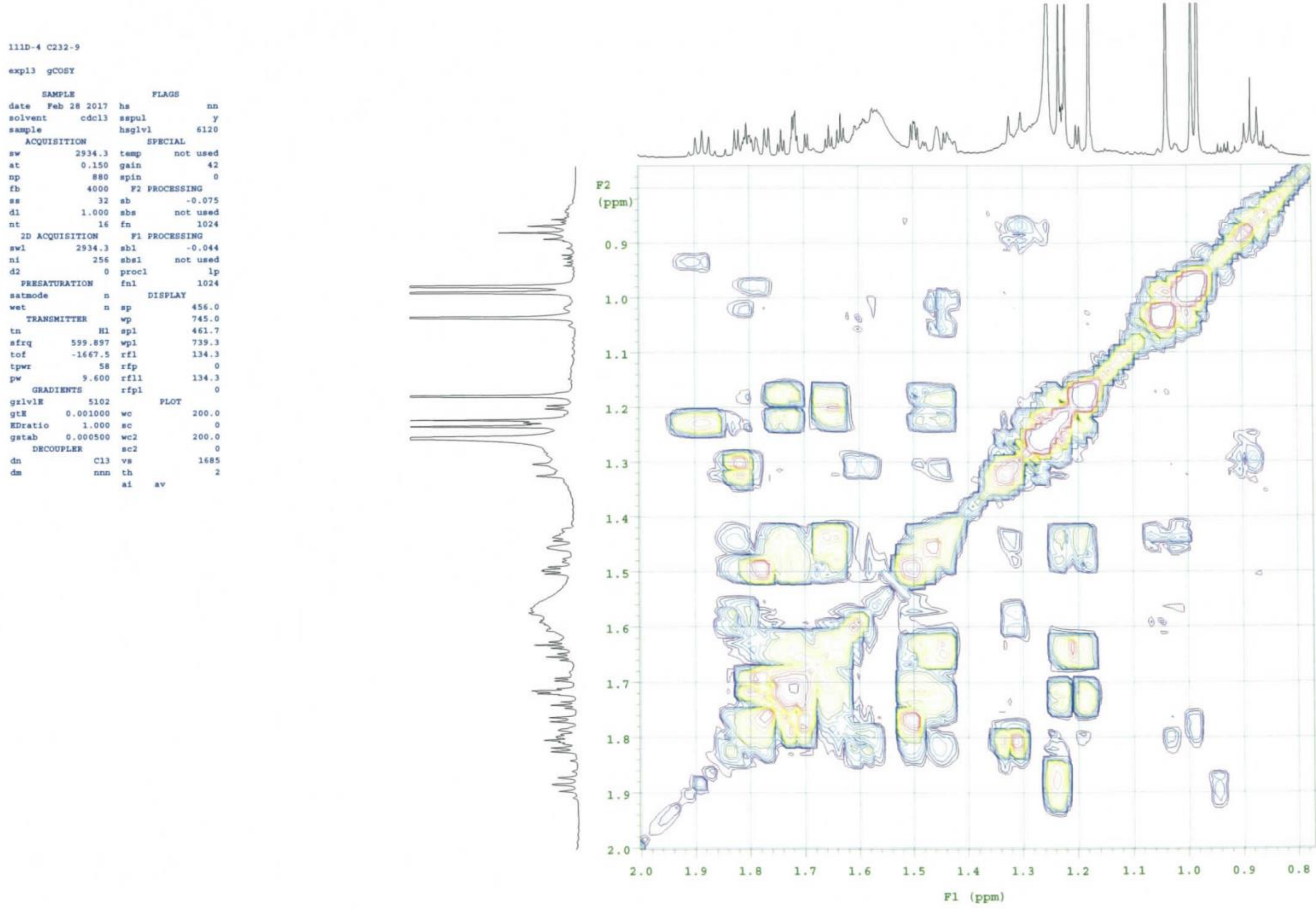
111D-4 C232-9
exp13 gCOSY
SAMPLE
date Feb 28 2017
solvent cdcl3
sample
ACQUISITION
sw 2934.3
at 0.150
np 880
fb 4000
ss 32
d1 1.000
nt 16
2D ACQUISITION
sw1 2934.3
ni 256
d2 0
PRESATURATION
satmode n
wet n
TRANSMITTER
tn H1
sfrq 599.897
tof -1667.5
tpwr 58
pw 9.600
GRADIENTS
gzlvlE 5102
gtE 0.001000
EDratio 1.000
gstab 0.000500
DECOUPLER
dn C13
dm nnn
FLAGS
hs nn
sspul y
hsglvl 6120
SPECIAL
temp not used
gain 42
spin 0
F2 PROCESSING
sb -0.075
sbs not used
fn 1024
F1 PROCESSING
sb1 -0.044
sbs1 not used
proc1 lp
fn1 1024
DISPLAY
sp 456.0
wp 745.0
sp1 461.7
wp1 739.3
rfl 134.3
rfp 0
rfl1 134.3
rfp1 0
PLOT
wc 200.0
sc 0
wc2 200.0
sc2 0
vs 1685
th 2
ai av
F2 (ppm)
F1 (ppm)

Figure S4 NOESY of 1

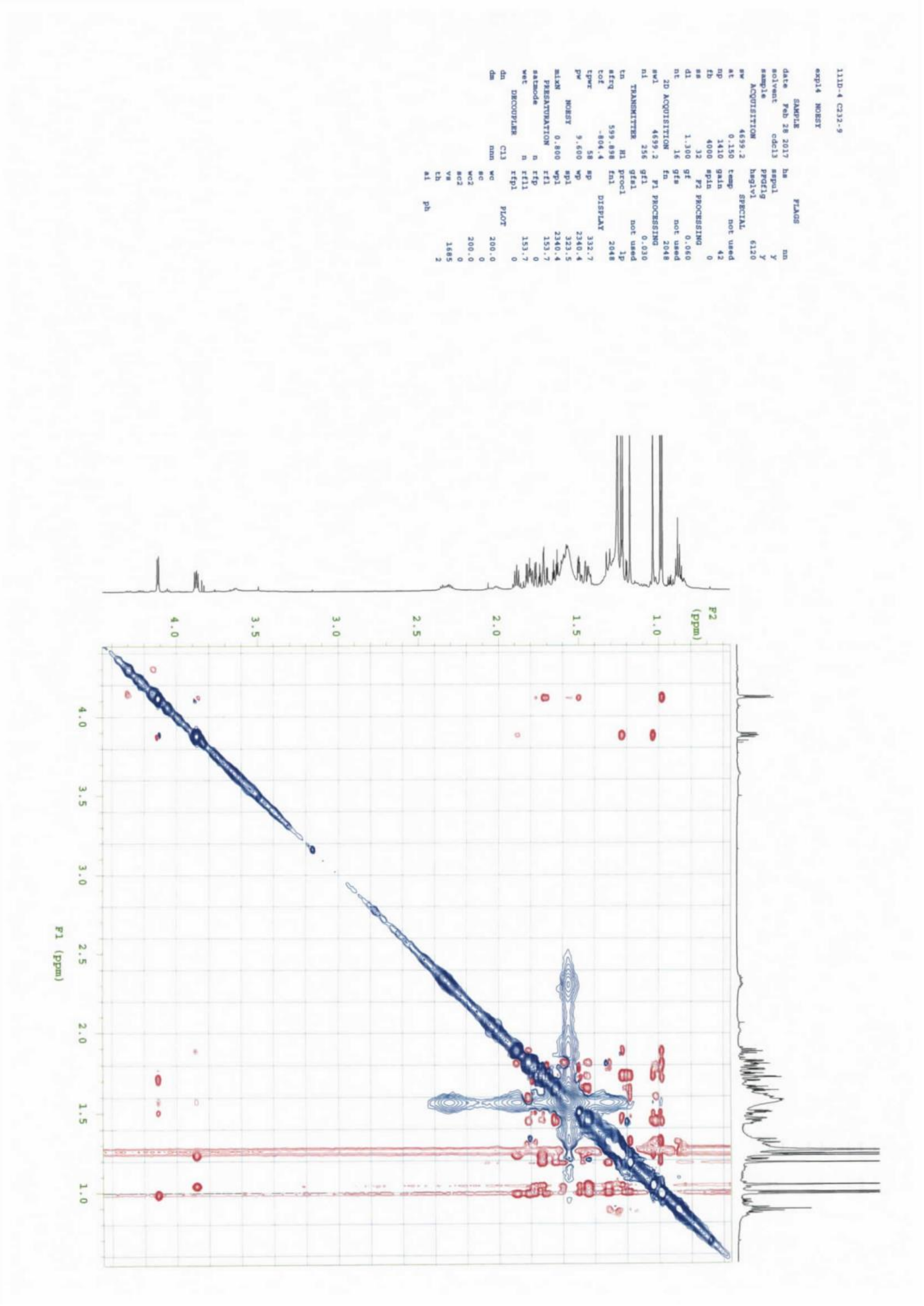

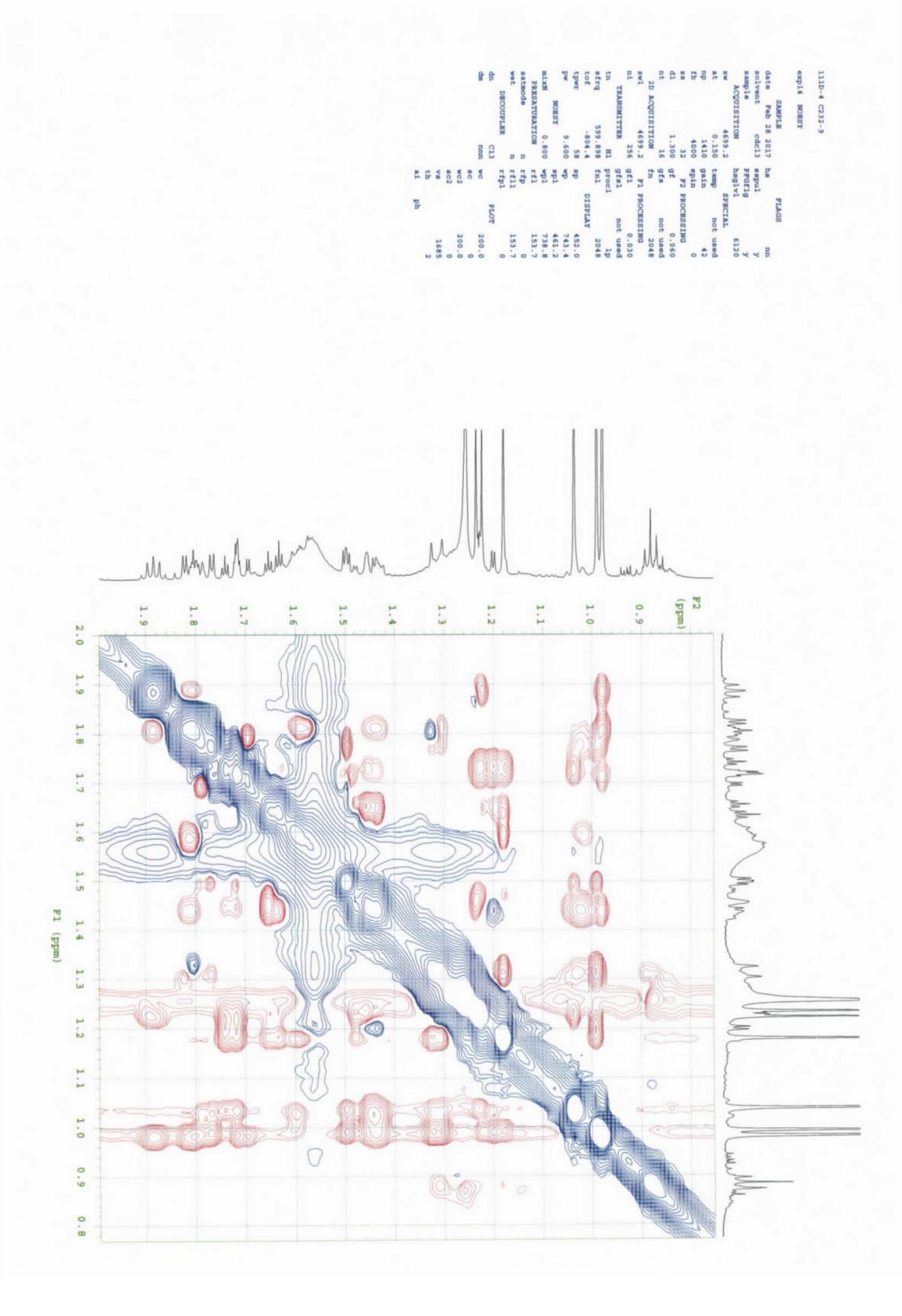
111D-4 C232-9
exp14 NOESY
SAMPLE
FLAGS
date Feb 28 2017
solvent cdcl3
F2 (ppm)
F1 (ppm)

Figure S5 HMQC of 1

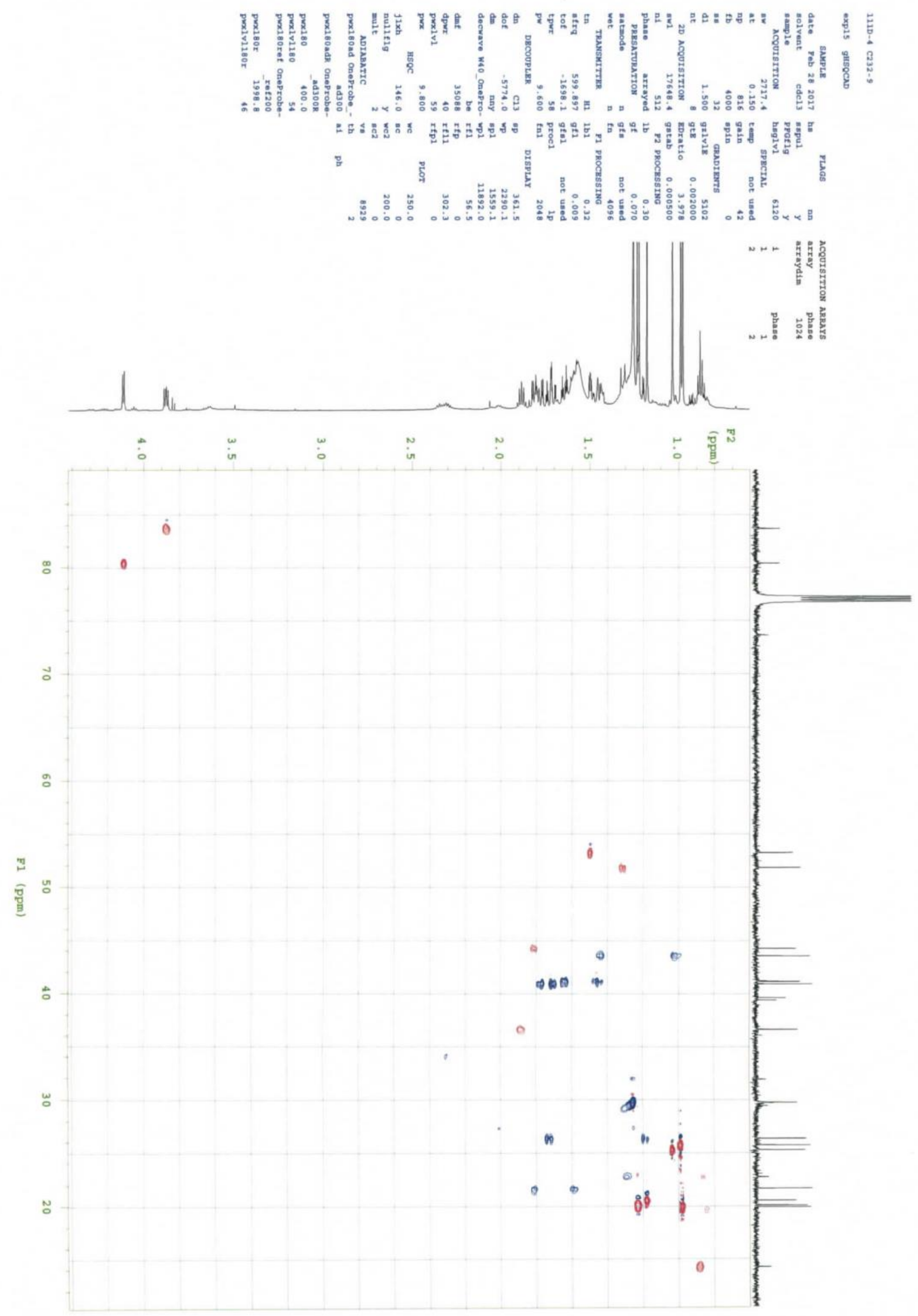

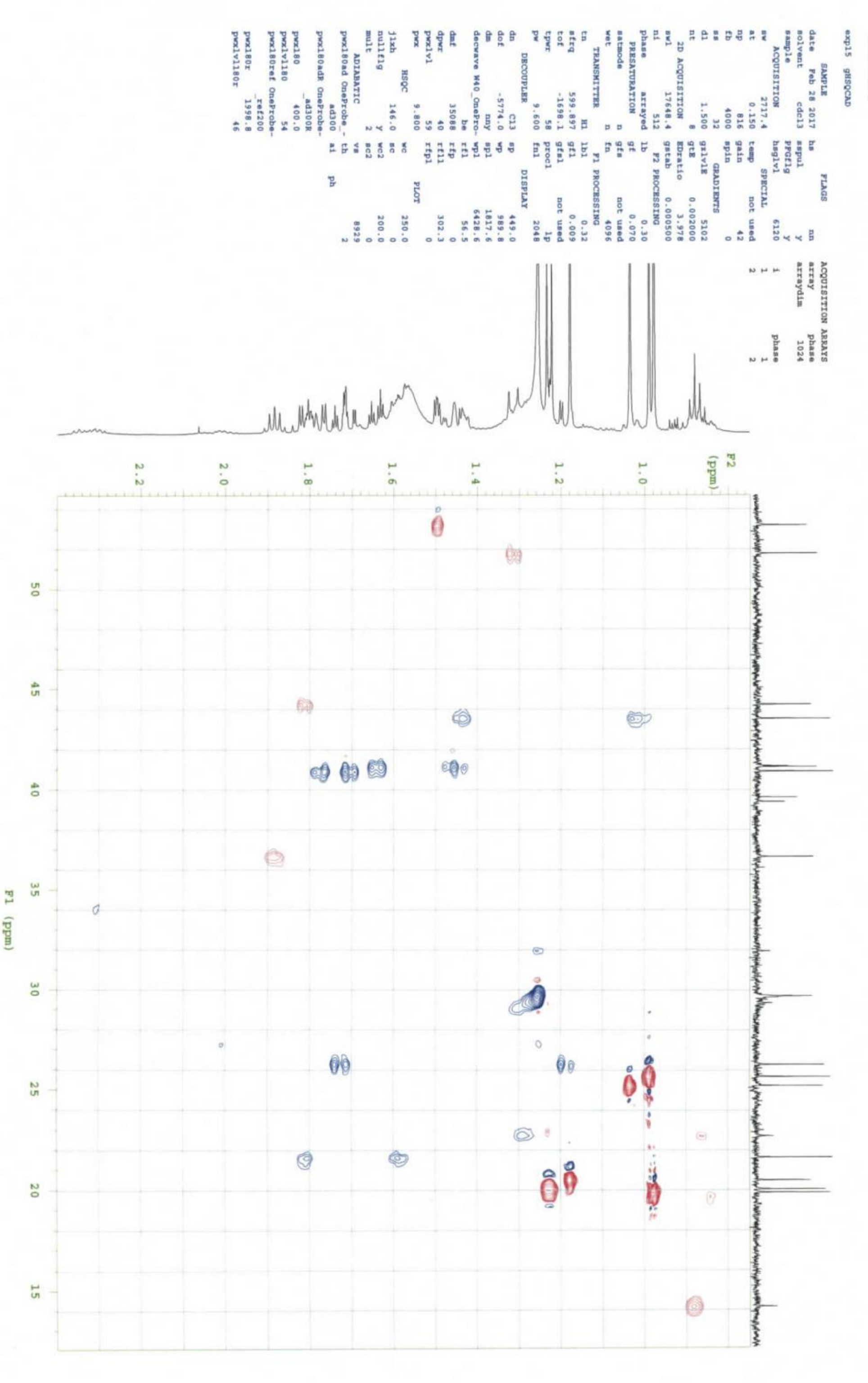

Figure S6 HMBC of 1

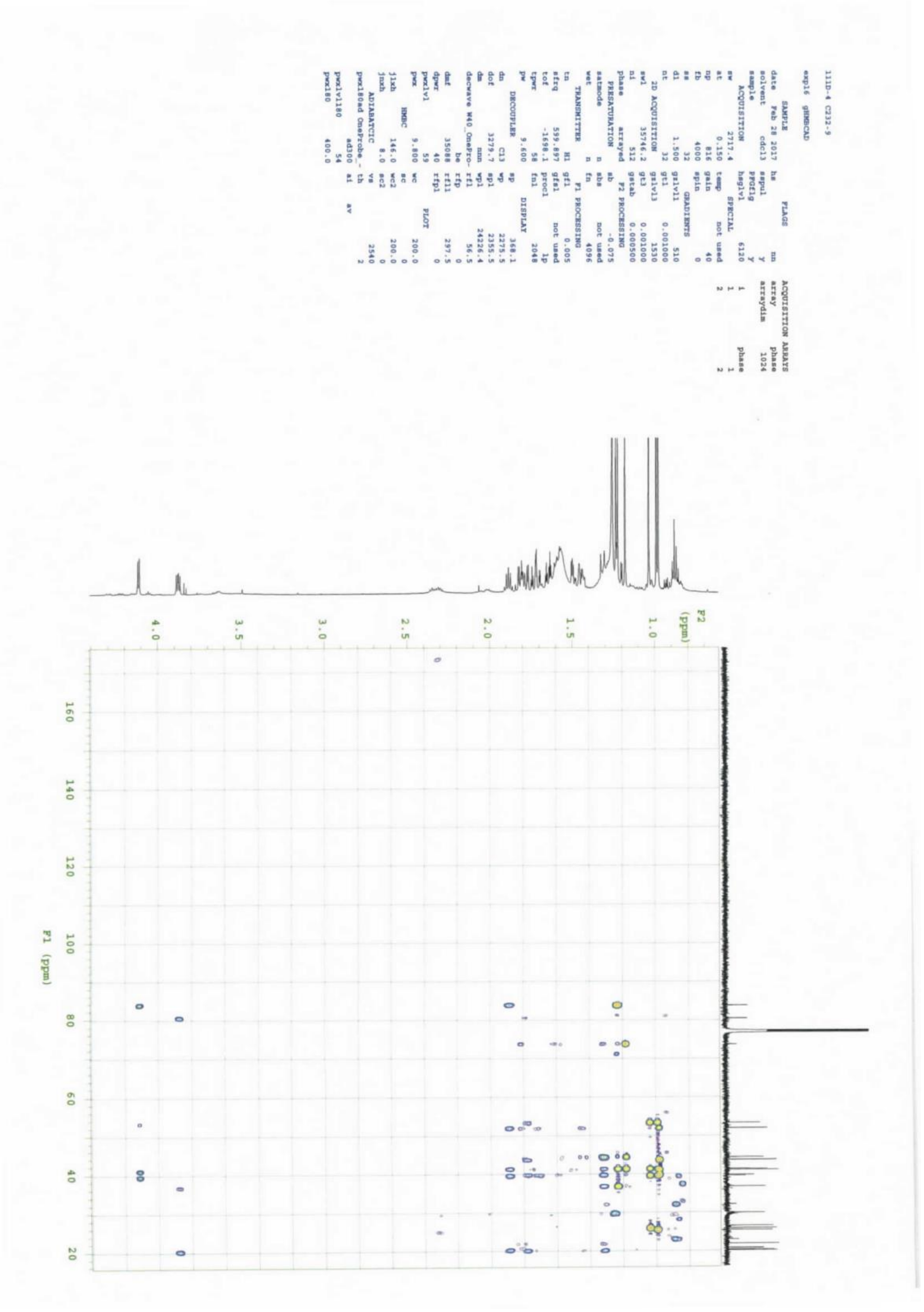

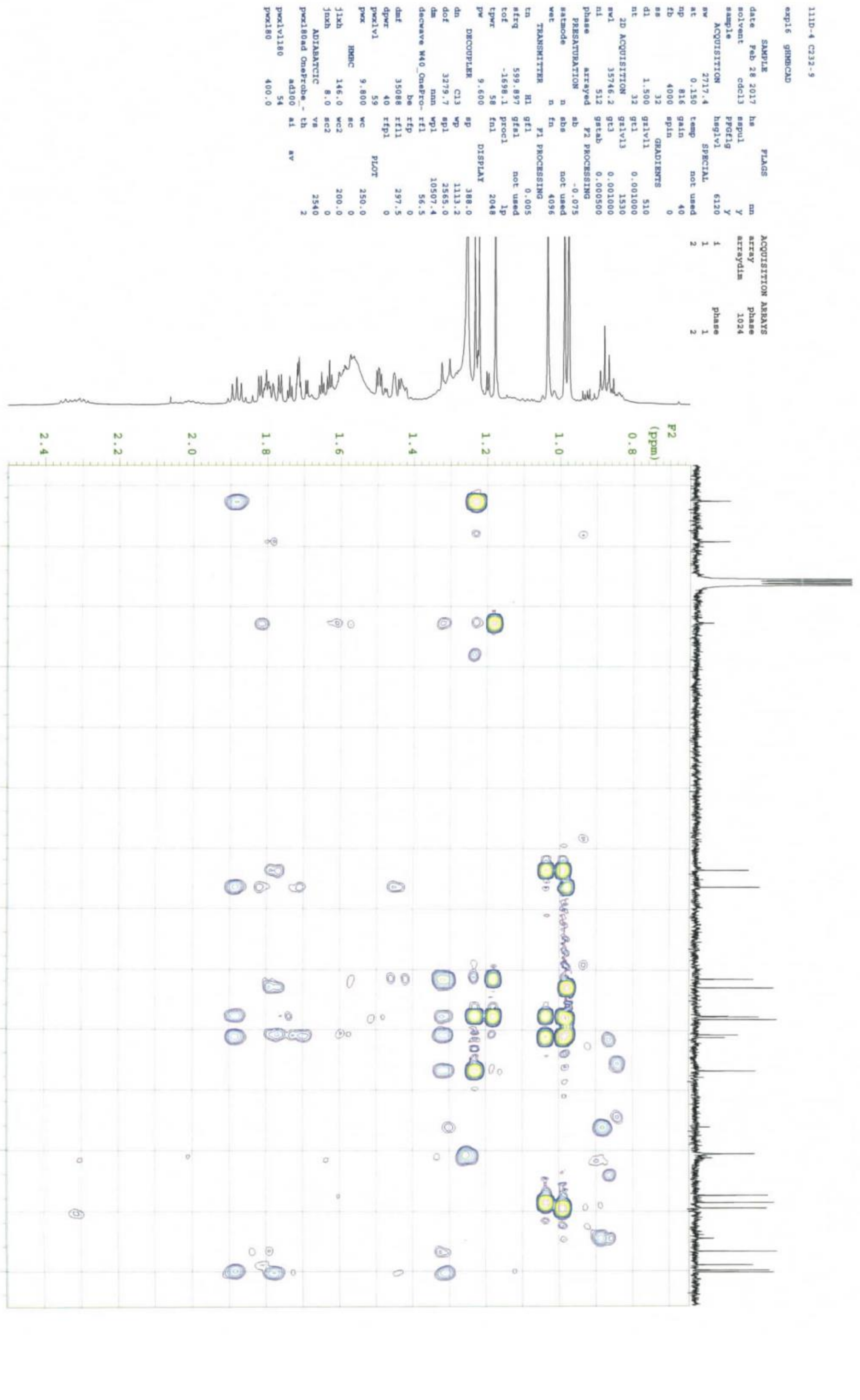
111D-4 C232-9
exp16 gHMBCAD
SAMPLE
date Feb 28 2017
solvent cdcl3
sample
ACQUISITION
sw 2717.4
at 0.150
np 816
fb 4000
ss 32
d1 1.500
nt 32
2D ACQUISITION
sw1 35746.2
ni 512
phase arrayed
PRESATURATION
satmode n
wet n
TRANSMITTER
tn H1
sfrq 599.897
tof -1698.1
tpwr 58
pw 9.600
DECOUPLER
dn C13
dof 3279.7
dm nnn
decwave W40_OnePro-be
dmf 35088
dpwr 40
pwxlvl 59
pwx 9.800
HMBC
j1xh 146.0
jnxh 8.0
ADIABATCIC
pwx180ad OneProbe_-ad300
pwxlvl180 54
pwx180 400.0
FLAGS
hs nn
sspul y
PFGflg y
hsglvl 6120
SPECIAL
temp not used
gain 40
spin 0
GRADIENTS
gzlvl1 510
gt1 0.001000
gzlvl3 1530
gt3 0.001000
gstab 0.000500
F2 PROCESSING
sb -0.075
sbs not used
fn 4096
F1 PROCESSING
gf1 0.005
gfs1 not used
proc1 lp
fn1 2048
DISPLAY
sp 388.0
wp 1113.2
sp1 2565.0
wp1 10507.4
rfl 56.5
rfp 0
rfl1 297.5
rfp1 0
PLOT
wc 250.0
sc 0
wc2 200.0
sc2 0
vs 2540
th 2
ai av
ACQUISITION ARRAYS
array phase
arraydim 1024
i phase
1 1
2 2
F2 (ppm)
0.8
1.0
1.2
1.4
1.6
1.8
2.0
2.2
2.4
80
70
60
50
40
30
20
F1 (ppm)

Figure S7 FABMS of 1

```
[ Elemental Composition ]                                           Page: 1
Data   : 1703086                    Date : 13-Mar-2017 19:27
Sample: 111D-4 C232-9-XXVII
Note : Matrix; G
Inlet : Direct                      Ion Mode : FAB+
RT : 7.32 min                       Scan#: (342,347)
Elements : C 25/15, H 40/30, O 8/0, Na 1/0
Mass Tolerance       : 20ppm, 1mmu if m/z > 50
Unsaturation (U.S.) : -1.0 - 30.0

Observed m/z Int%   Err[ppm / mmu]    U.S.  Composition
  345.2397   100.0     -2.6 /  -0.9    3.5  C 20 H 34 O 3 Na
```

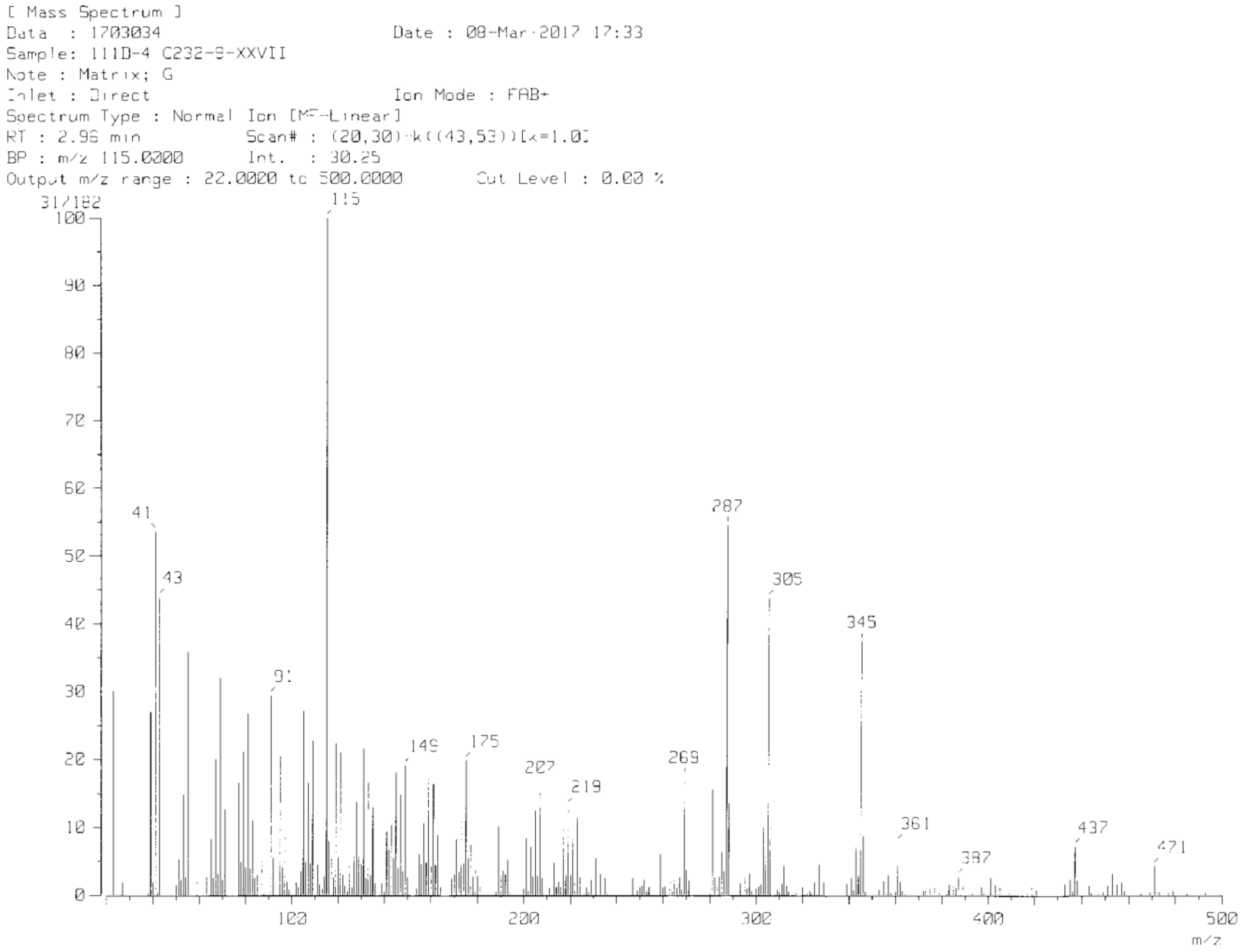
[Mass Spectrum]
Data : 1703034
Date : 08-Mar-2017 17:33
Sample: 111D-4 C232-9-XXVII
Note : Matrix; G
Inlet : Direct
Ion Mode : FAB+
Spectrum Type : Normal Ion [MF-Linear]
RT : 2.96 min
Scan# : (20,30)-k((43,53))[x=1.0]
BP : m/z 115.0000
Int. : 30.25
Output m/z range : 22.0000 to 500.0000
Cut Level : 0.00 %
31/182
100
90
80
70
60
50
40
30
20
10
0
41
43
91
115
149
175
207
219
269
287
305
345
361
387
437
471
100
200
300
400
500
m/z

Figure S8 ^{1}H NMR spectrum of 2 in $CDCl_3$

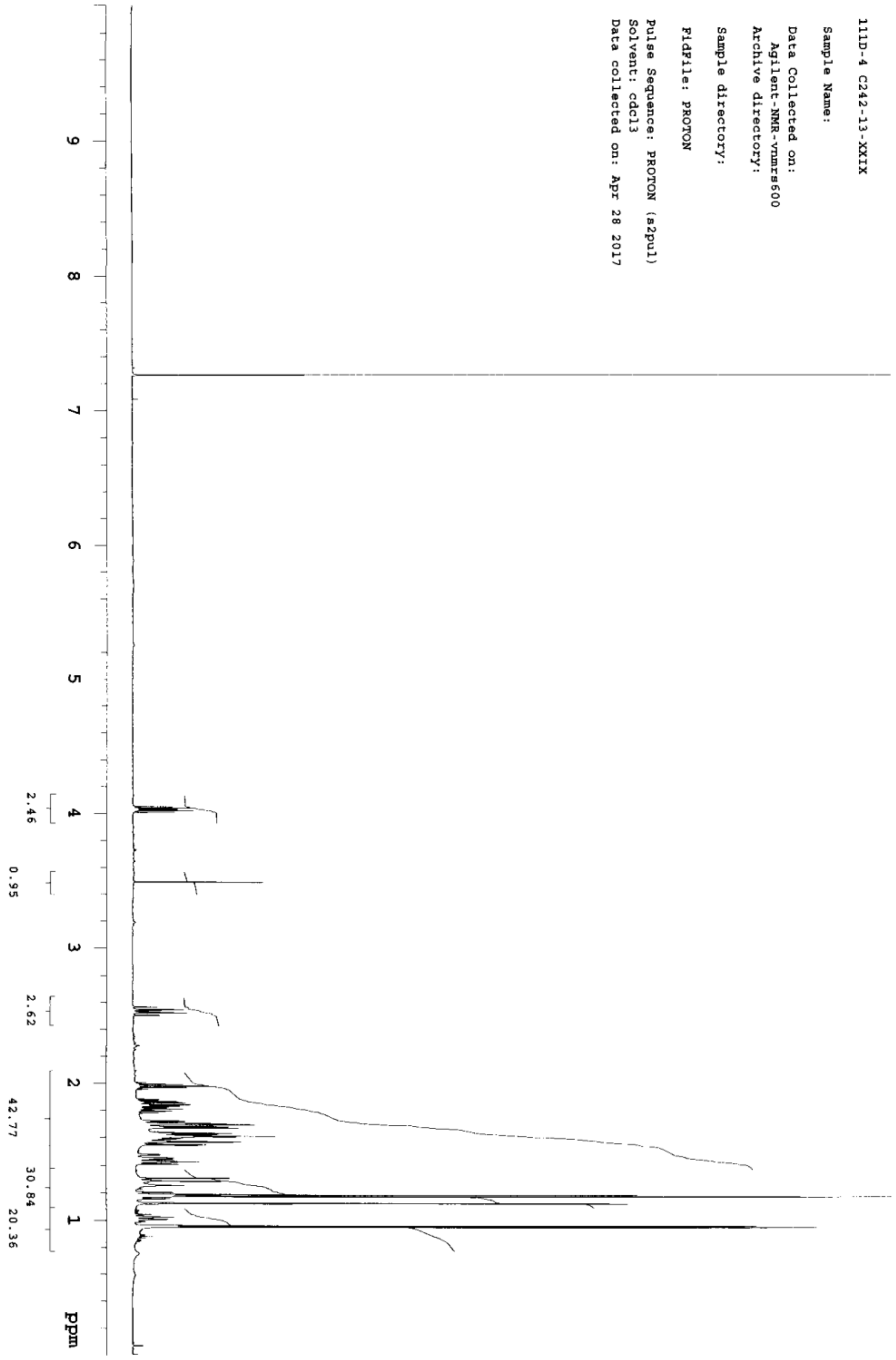

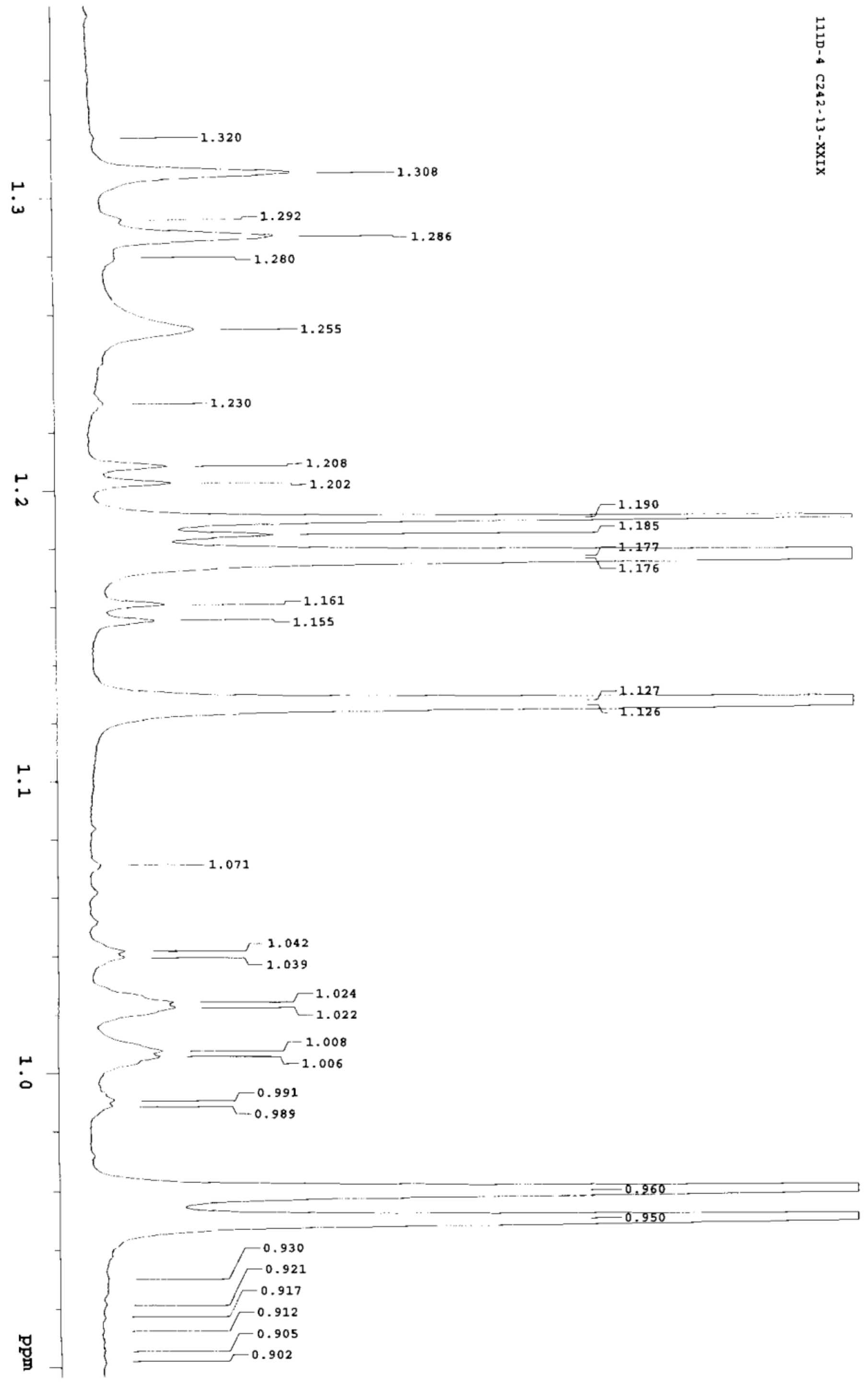
111D-4 C242-13-XXIX
1.320
1.308
1.292
1.286
1.280
1.255
1.230
1.208
1.202
1.190
1.185
1.177
1.176
1.161
1.155
1.127
1.126
1.071
1.042
1.039
1.024
1.022
1.008
1.006
0.991
0.989
0.960
0.950
0.930
0.921
0.917
0.912
0.905
0.902
1.3
1.2
1.1
1.0
ppm

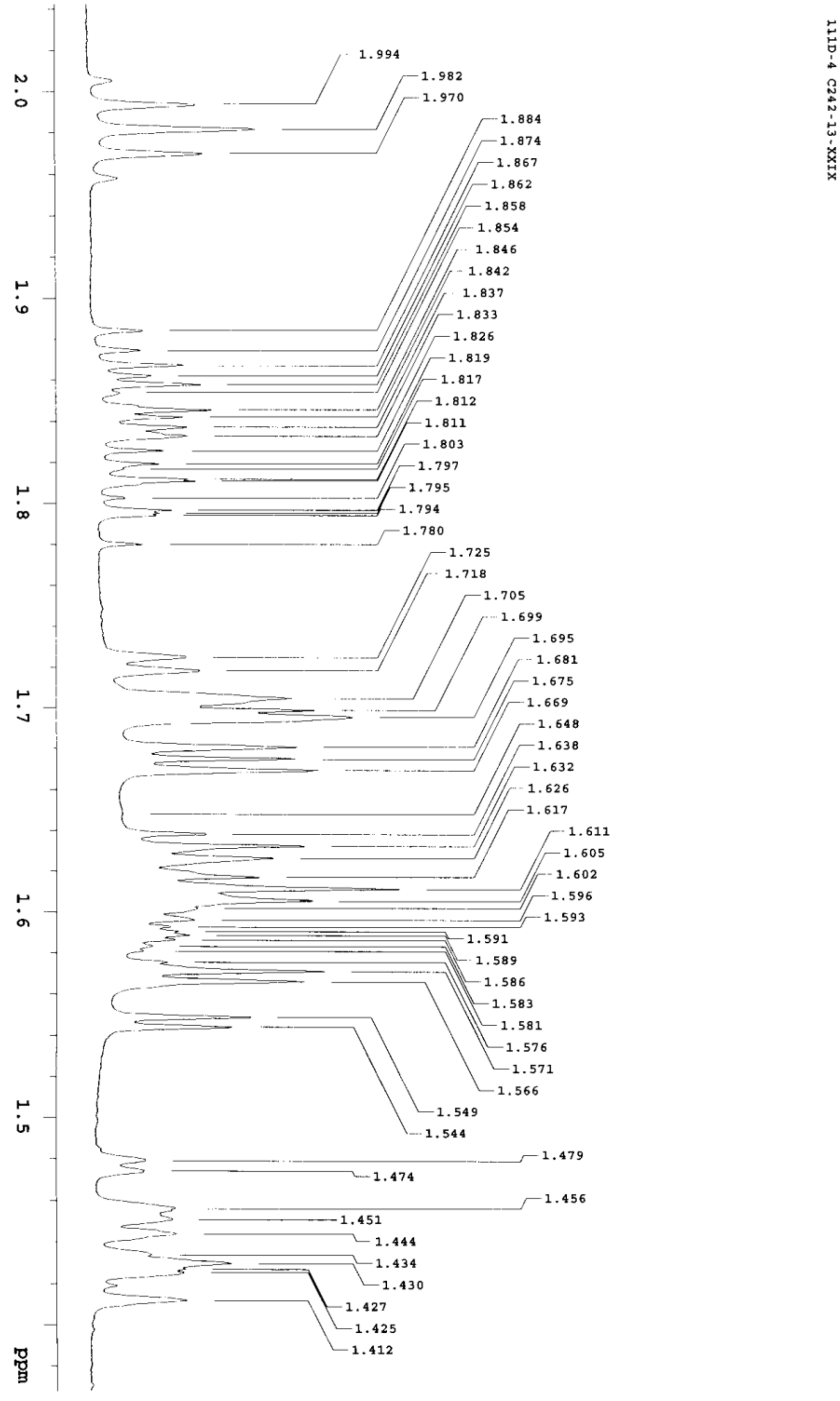
111D-4 C242-13-XXIX
2.0
1.9
1.8
1.7
1.6
1.5
ppm
1.994
1.982
1.970
1.884
1.874
1.867
1.862
1.858
1.854
1.846
1.842
1.837
1.833
1.826
1.819
1.817
1.812
1.811
1.803
1.797
1.795
1.794
1.780
1.725
1.718
1.705
1.699
1.695
1.681
1.675
1.669
1.648
1.638
1.632
1.626
1.617
1.611
1.605
1.602
1.596
1.593
1.591
1.589
1.586
1.583
1.581
1.576
1.571
1.566
1.549
1.544
1.479
1.474
1.456
1.451
1.444
1.434
1.430
1.427
1.425
1.412

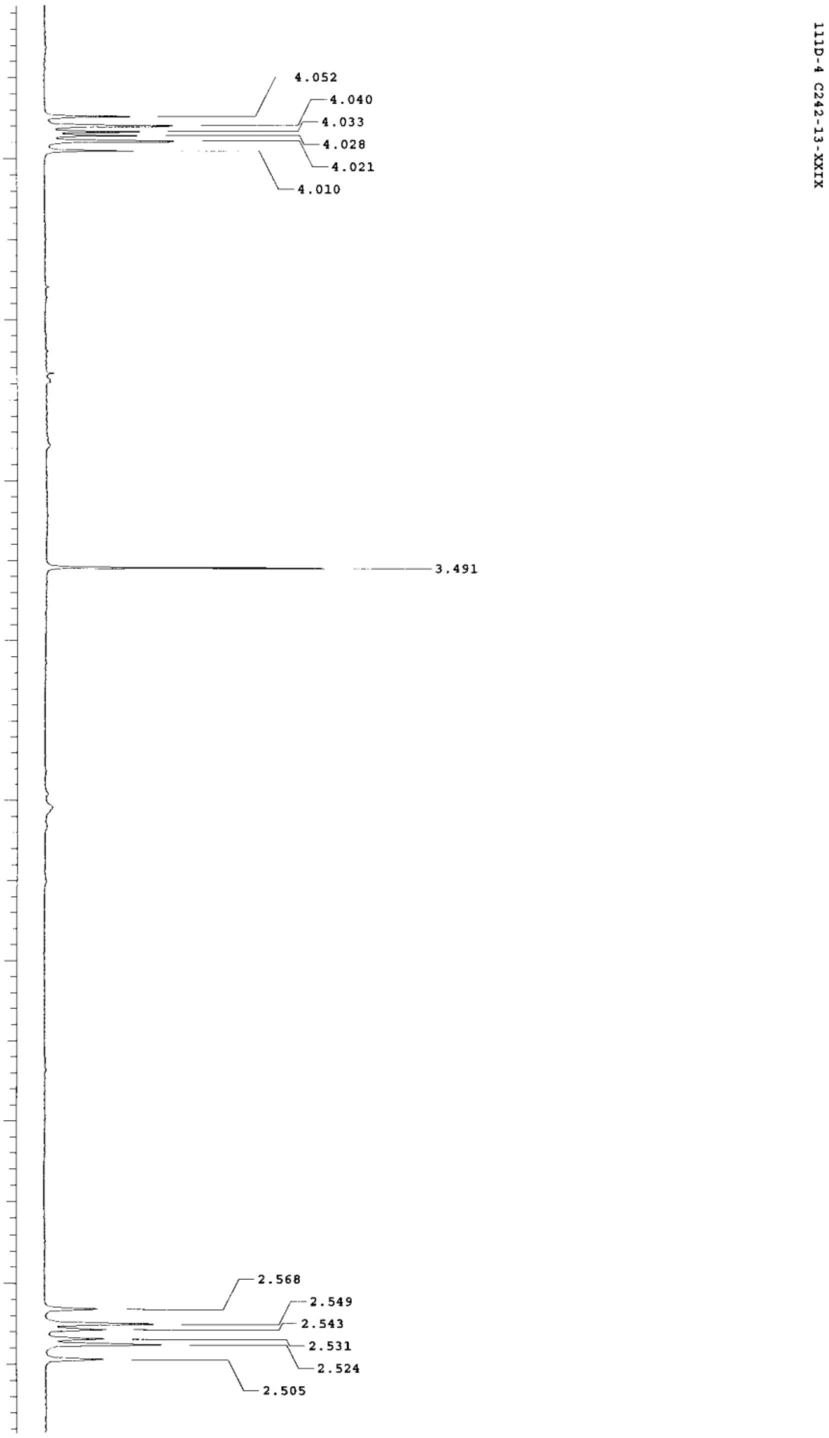
111D-4 C242-13-XXIX
4.052
4.040
4.033
4.028
4.021
4.010
3.491
2.568
2.549
2.543
2.531
2.524
2.505
4.0
3.8
3.6
3.4
3.2
3.0
2.8
2.6
ppm

Figure S9 ^{13}C NMR spectrum of 2 in $CDCl_3$

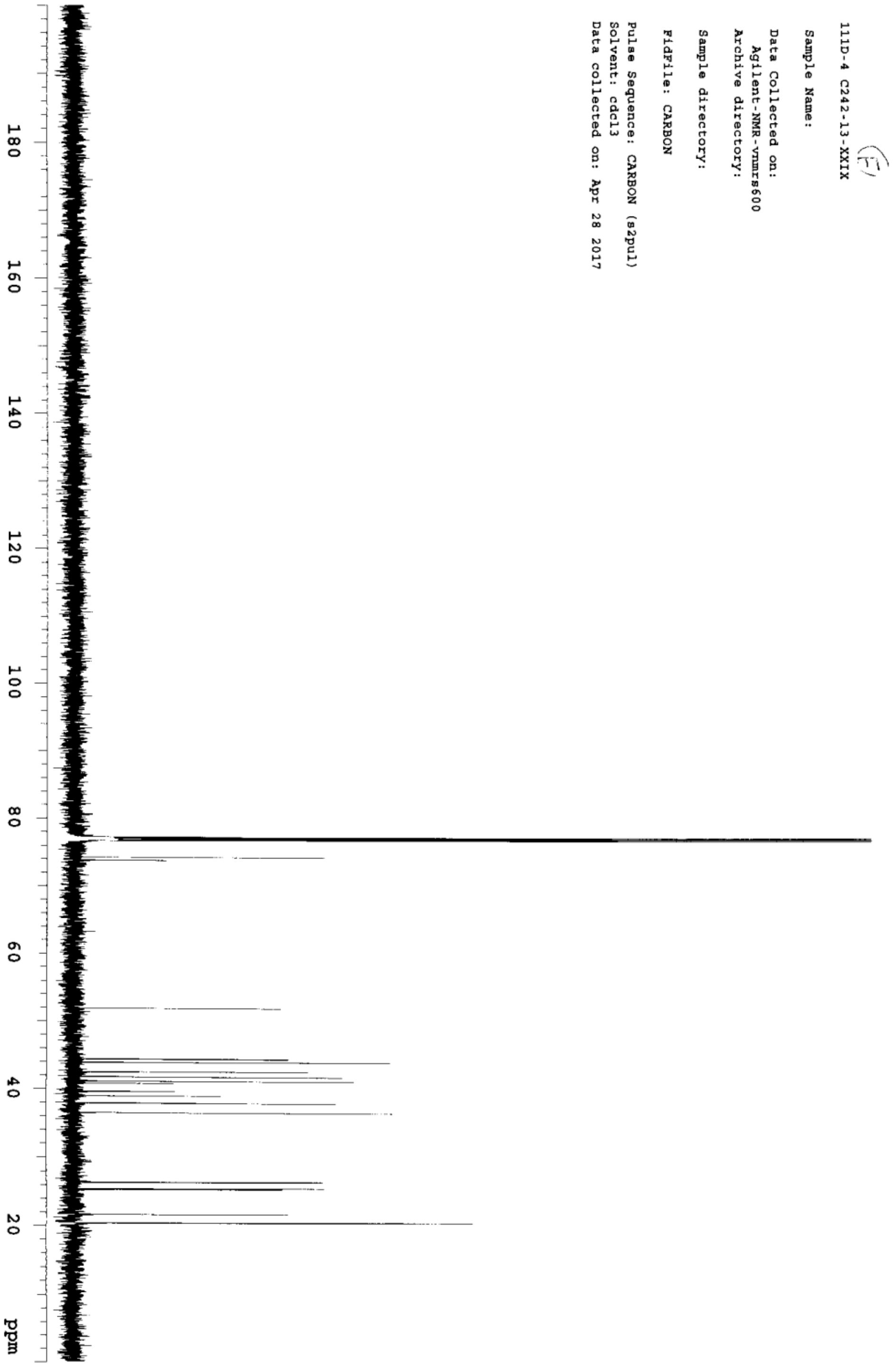

111D-4 C242-13-XXIX

INDEX	FREQUENCY	PPM	HEIGHT
1	11647.3	77.215	654.5
2	11615.0	77.000	676.4
3	11582.6	76.785	593.0
4	11197.7	74.234	48.8
5	11132.9	73.804	18.2
6	7824.6	51.872	40.5
7	6698.6	44.408	41.9
8	6623.5	43.910	61.6
9	6412.0	42.507	45.6
10	6304.5	41.795	52.3
11	6207.4	41.151	54.9
12	6157.6	40.821	19.5
13	5980.8	39.649	19.8
14	5881.4	38.990	28.7
15	5719.5	37.917	51.1
16	5503.4	36.484	62.5
17	3976.3	26.361	48.4
18	3836.5	25.433	49.1
19	3815.7	25.295	40.6
20	3265.4	21.648	41.8
21	3080.5	20.422	77.7
22	3071.2	20.360	66.4

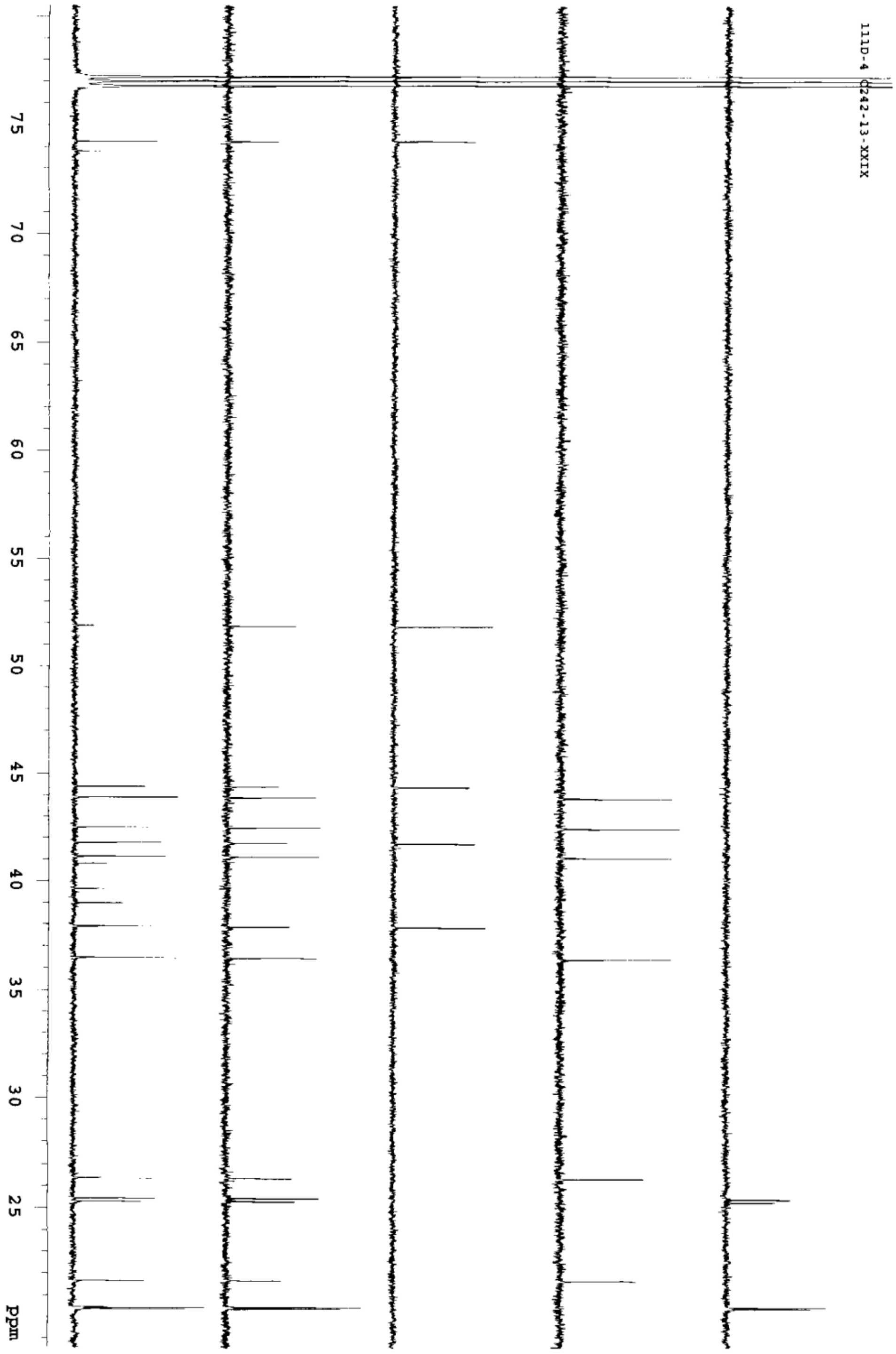
111D-4 C242-13-XXIX
75
70
65
60
55
50
45
40
35
30
25
ppm

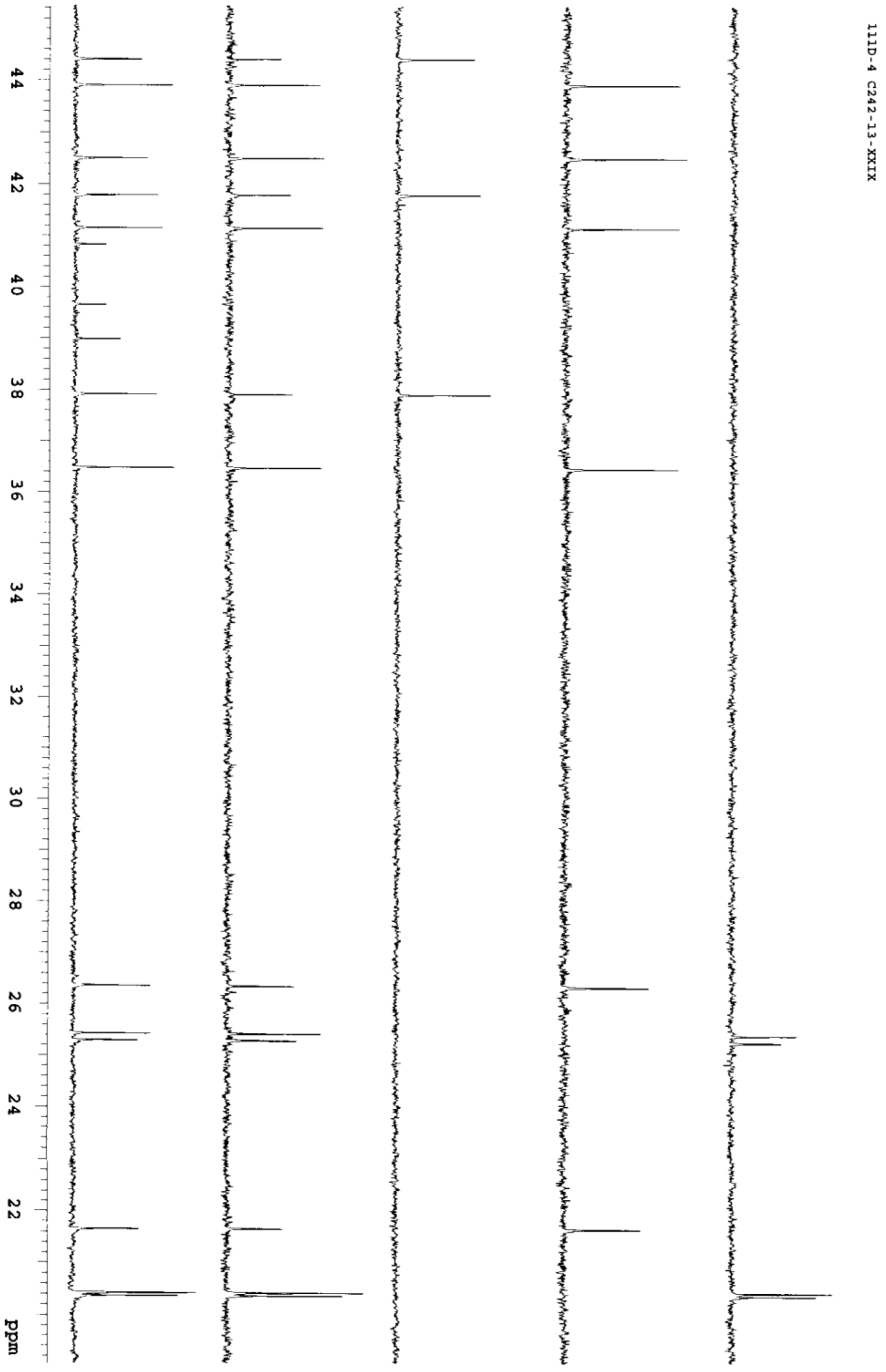
111D-4 C242-13-XXIX
44
42
40
38
36
34
32
30
28
26
24
22
ppm

Figure S10 ^{1}H-^{1}H COSY of 2

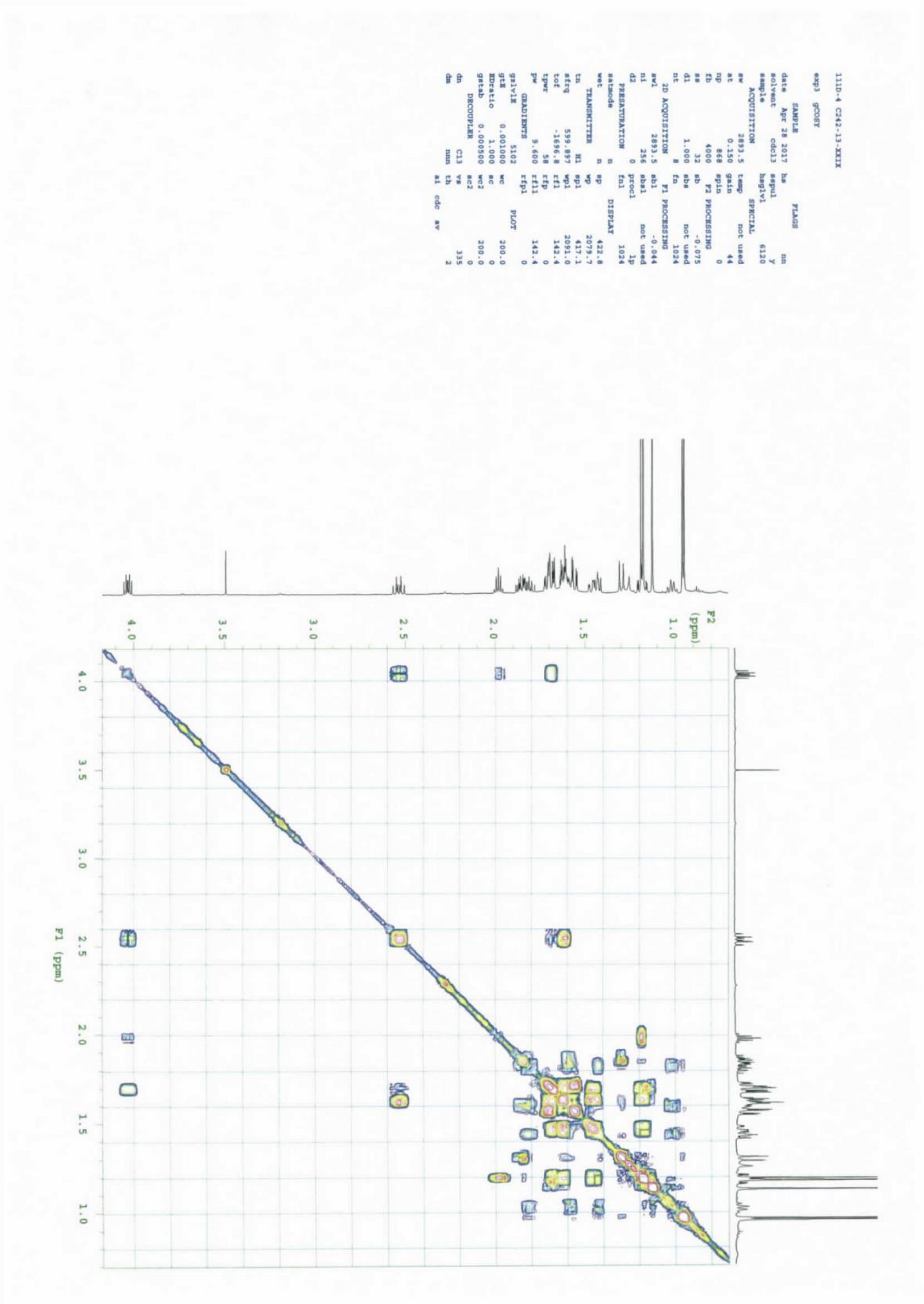

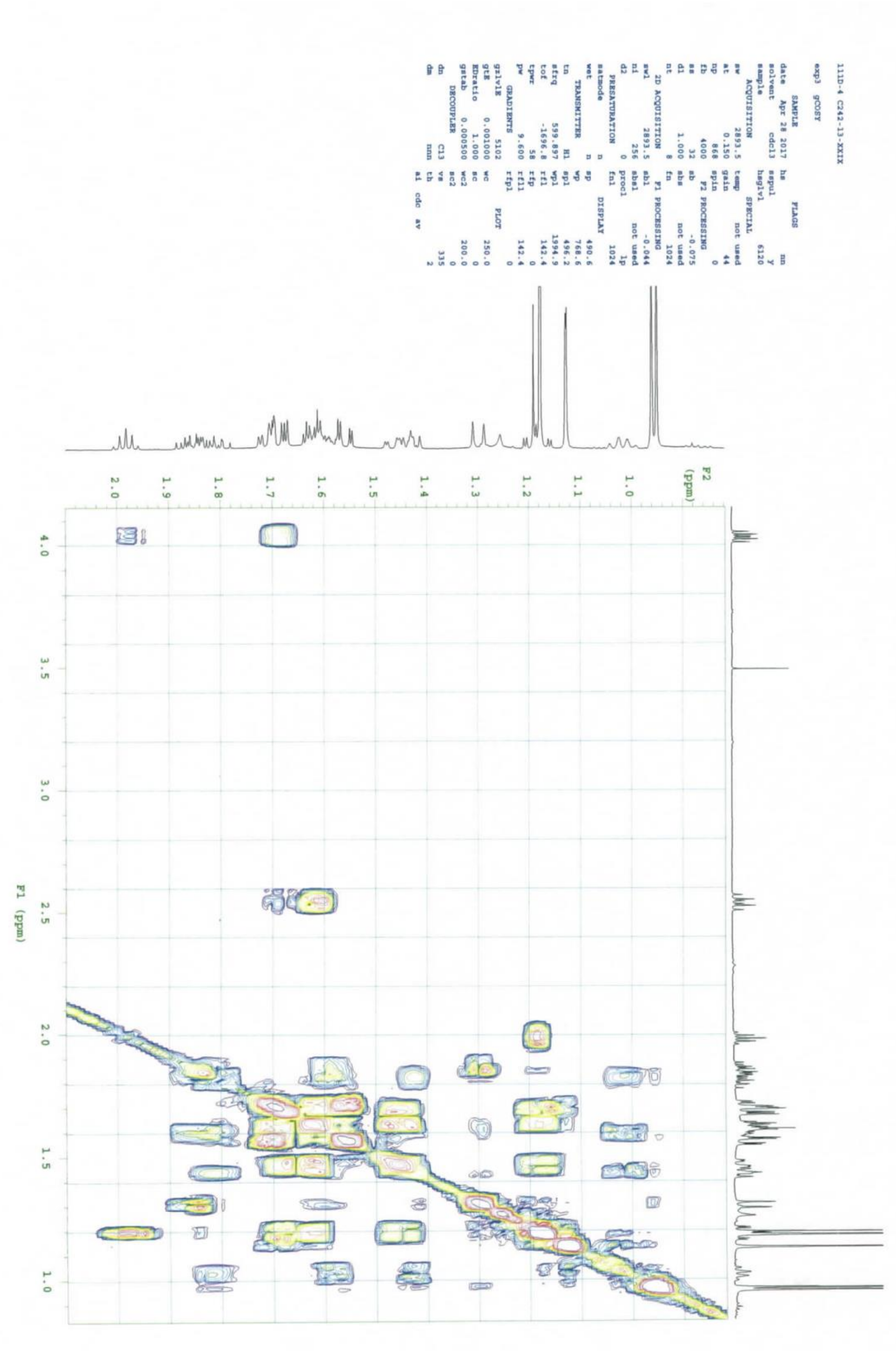
111D-4 C242-13-XXIX
exp3 gCOSY
SAMPLE
date Apr 28 2017
solvent cdcl3
sample
ACQUISITION
sw 2893.5
at 0.150
np 868
fb 4000
ss 32
d1 1.000
nt 8
2D ACQUISITION
sw1 2893.5
ni 256
d2 0
PRESATURATION
satmode n
wet n
TRANSMITTER
tn H1
sfrq 599.897
tof -1696.8
tpwr 58
pw 9.600
GRADIENTS
gzlvlE 5102
gtE 0.001000
EDratio 1.000
gstab 0.000500
DECOUPLER
dn C13
dm nnn
FLAGS
hs nn
sspul y
hsglvl 6120
SPECIAL
temp not used
gain 44
spin 0
F2 PROCESSING
sb -0.075
sbs not used
fn 1024
F1 PROCESSING
sb1 -0.044
sbs1 not used
proc1 lp
fn1 1024
DISPLAY
sp 490.6
wp 768.6
sp1 496.2
wp1 1994.9
rfl 142.4
rfp 0
rfl1 142.4
rfp1 0
PLOT
wc 250.0
sc 0
wc2 200.0
sc2 0
vs 335
th 2
ai cdc av
F2 (ppm)
1.0
1.1
1.2
1.3
1.4
1.5
1.6
1.7
1.8
1.9
2.0
4.0
3.5
3.0
2.5
2.0
1.5
1.0
F1 (ppm)

Figure S11 NOESY of 2

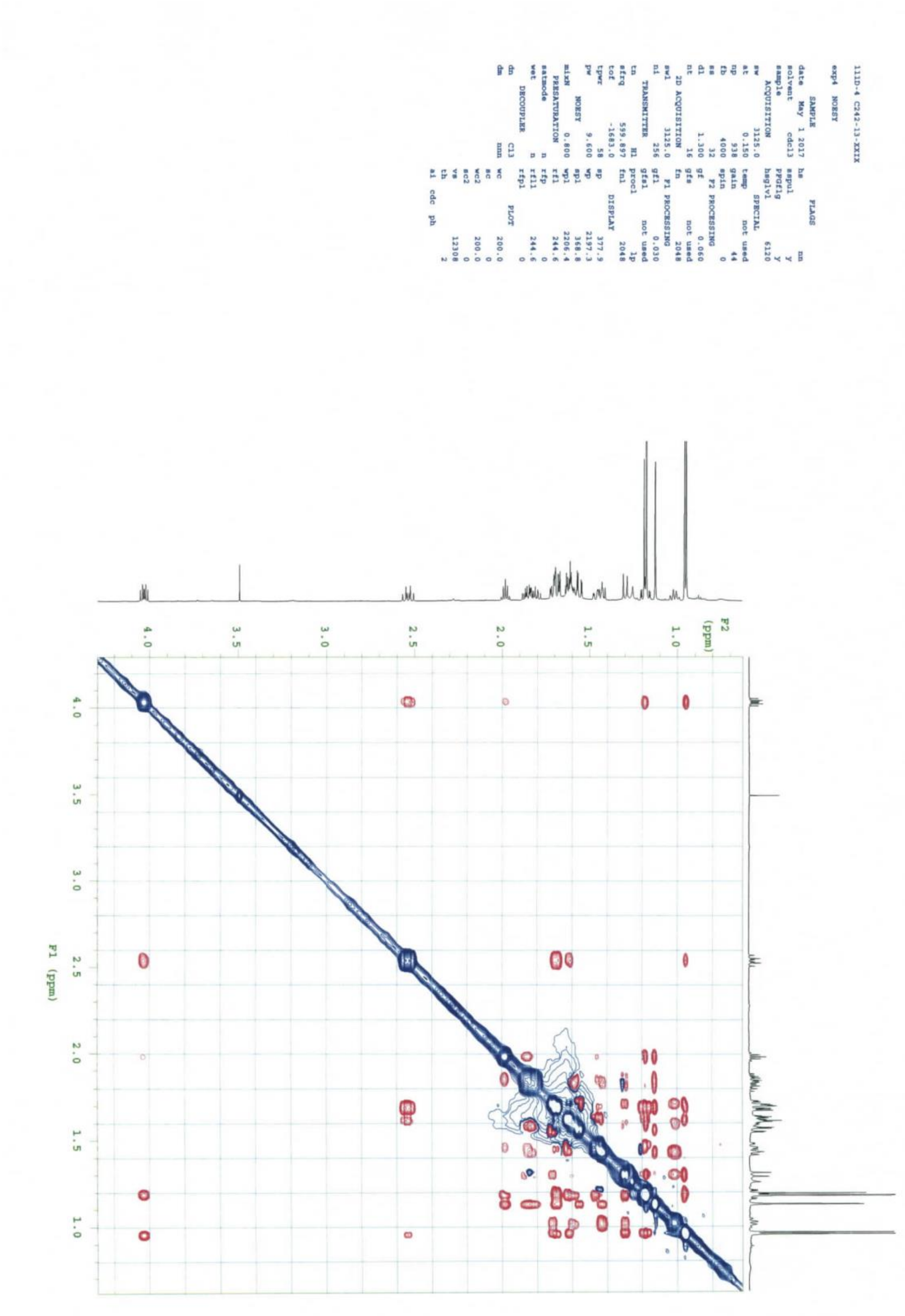

111D-4 C242-13-XXIX

exp4 NOESY

SAMPLE		FLAGS	
date	May 1 2017	hs	nn
solvent	cdcl3	sspul	y
sample		PFGflg	y
ACQUISITION		hsglvl	6120
sw	3125.0	SPECIAL	
at	0.150	temp	not used
np	938	gain	44
fb	4000	spin	0
ss	32	F2 PROCESSING	
d1	1.300	gf	0.060
nt	16	gfs	not used
2D ACQUISITION		fn	2048
sw1	3125.0	F1 PROCESSING	
ni	256	gf1	0.030
TRANSMITTER		gfs1	not used
tn	H1	proc1	lp
sfrq	599.897	fn1	2048
tof	-1683.0	DISPLAY	
tpwr	58	sp	490.9
pw	9.600	wp	787.4
NOESY		sp1	487.8
mixN	0.800	wp1	790.4
PRESATURATION		rfl	244.6
satmode	n	rfp	0
wet	n	rfl1	244.6
DECOUPLER		rfp1	0
dn	C13	PLOT	
dm	nnn	wc	200.0
		sc	0
		wc2	200.0
		sc2	0
		vs	12308
		th	2
		ai cdc ph	

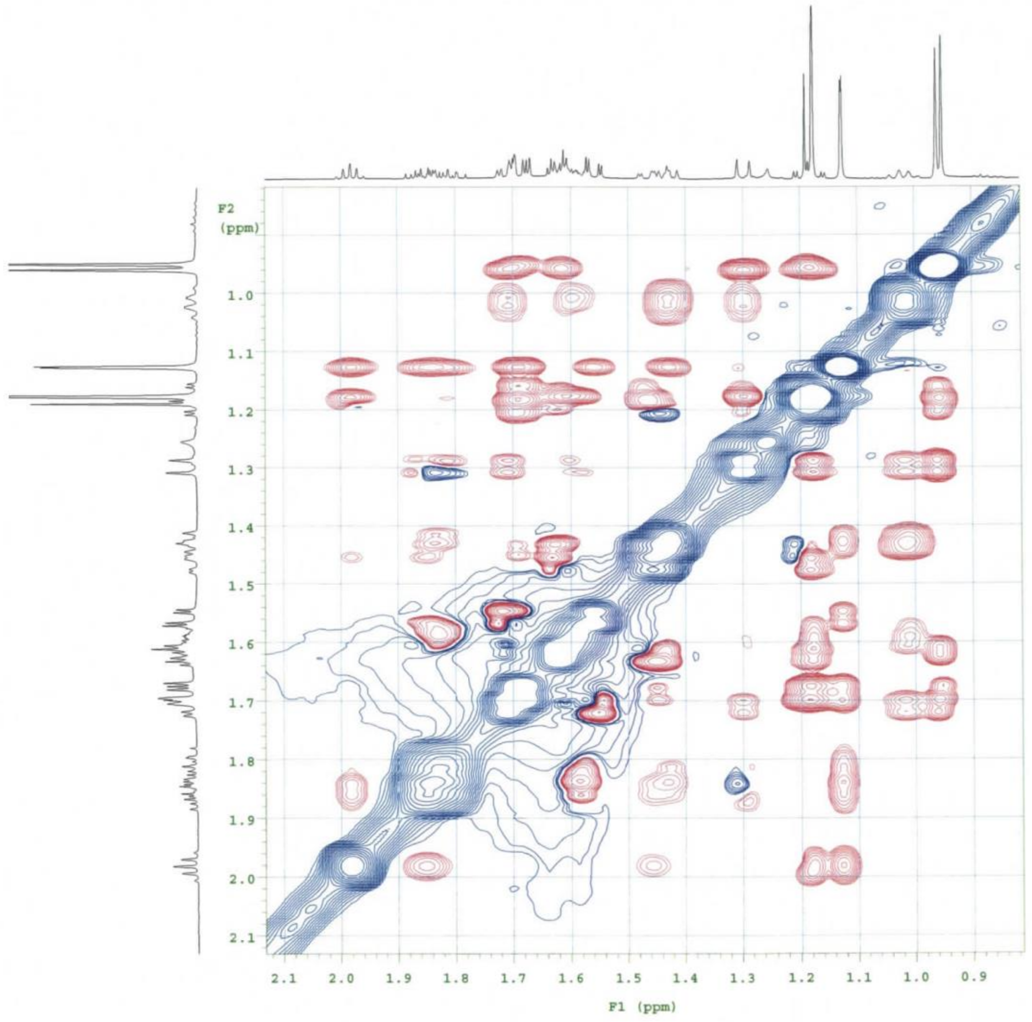

Figure S12 HMQC of 2

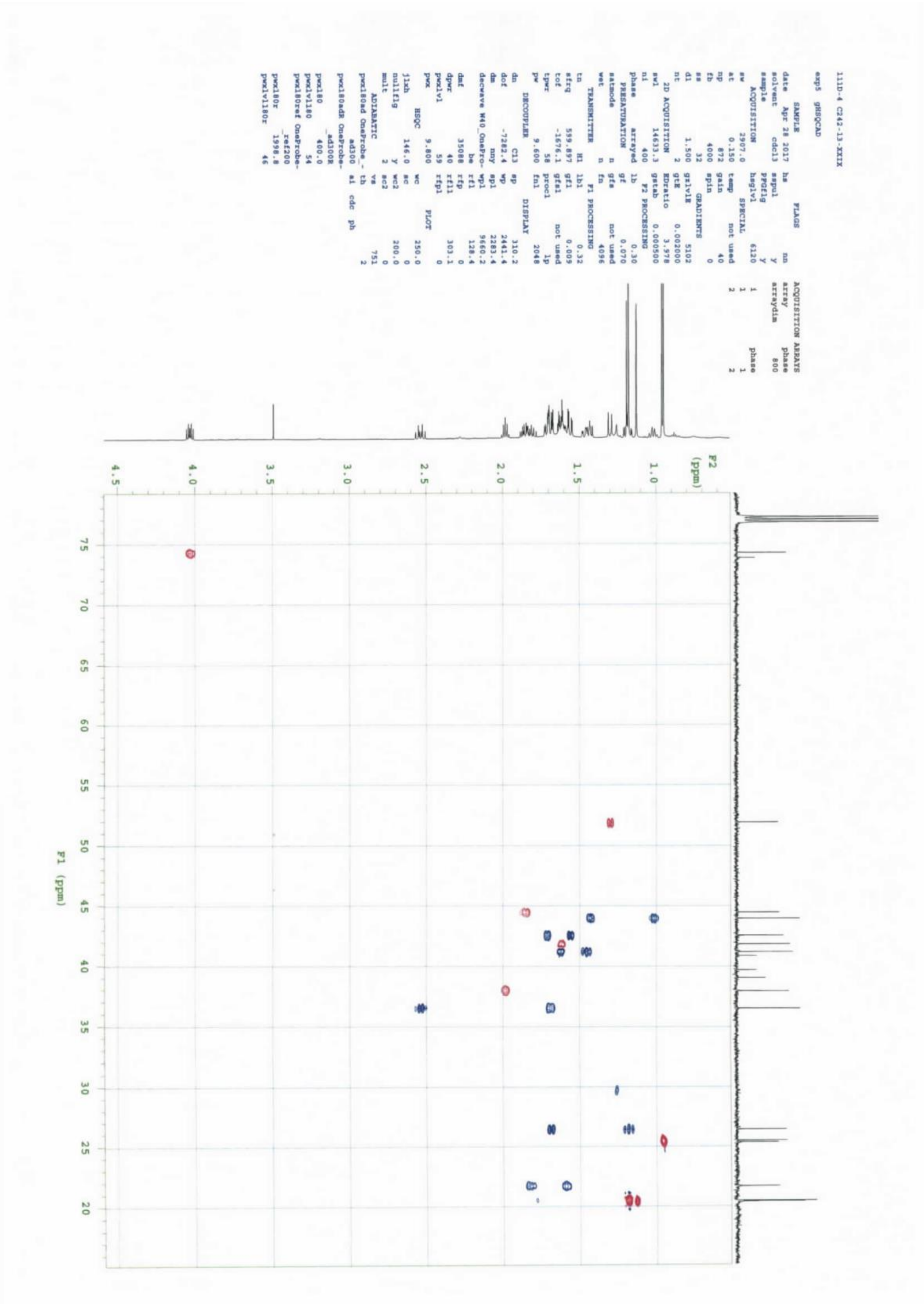

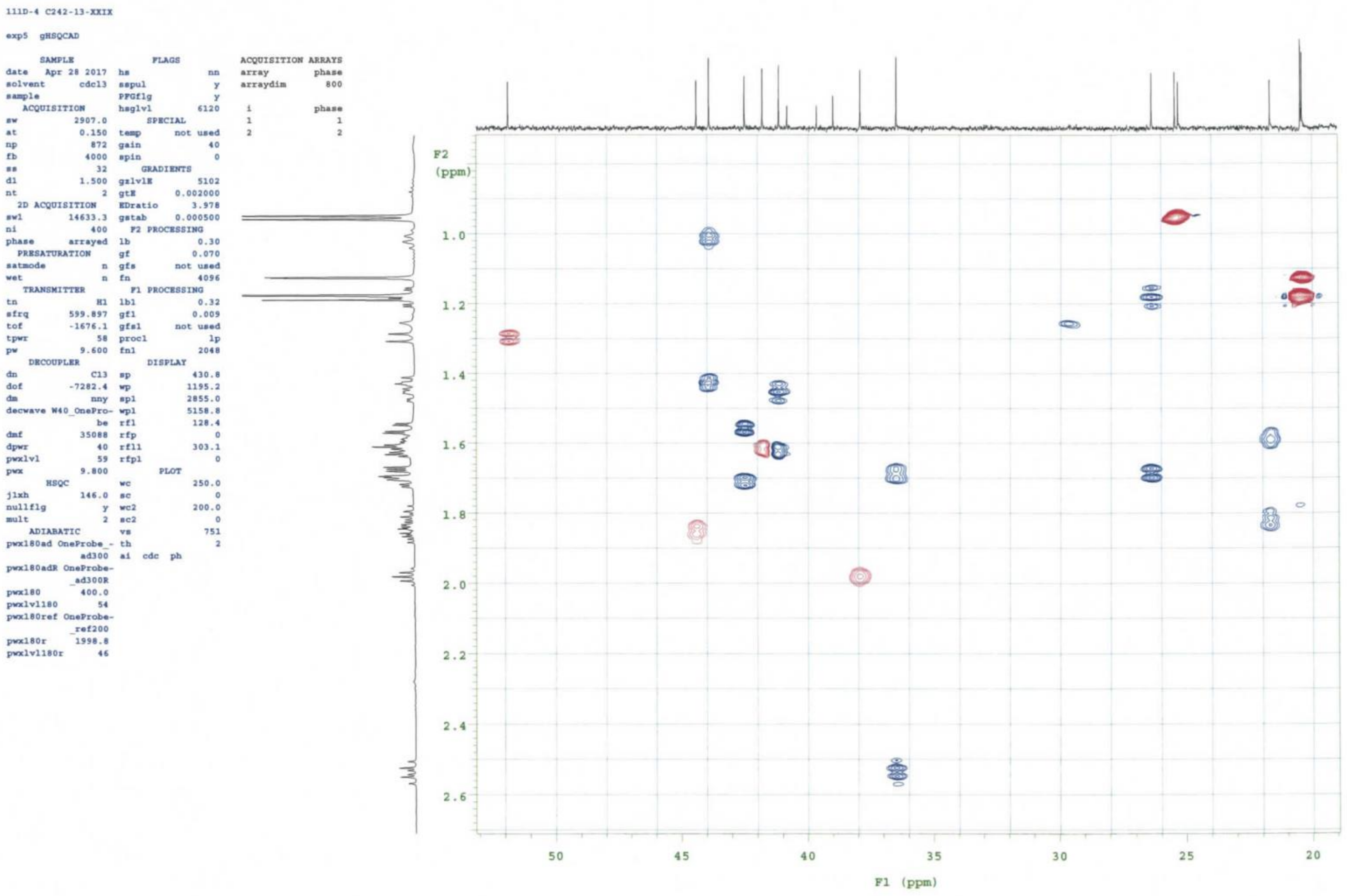

111D-4 C242-13-XXIX
exp5 gHSQCAD
SAMPLE
date Apr 28 2017
solvent cdcl3
sample
ACQUISITION
sw 2907.0
at 0.150
np 872
fb 4000
ss 32
d1 1.500
nt 2
2D ACQUISITION
sw1 14633.3
ni 400
phase arrayed
PRESATURATION
satmode n
wet n
TRANSMITTER
tn H1
sfrq 599.897
tof -1676.1
tpwr 58
pw 9.600
DECOUPLER
dn C13
dof -7282.4
dm nny
decwave W40_OnePro-be
dmf 35088
dpwr 40
pwxlvl 59
pwx 9.800
HSQC
j1xh 146.0
nullflg y
mult 2
ADIABATIC
pwx180ad OneProbe_-ad300
pwx180adR OneProbe-_ad300R
pwx180 400.0
pwxlvl180 54
pwx180ref OneProbe-_ref200
pwx180r 1998.8
pwxlvl180r 46
FLAGS
hs nn
sspul y
PFGflg y
hsglvl 6120
SPECIAL
temp not used
gain 40
spin 0
GRADIENTS
gzlvlE 5102
gtE 0.002000
EDratio 3.978
gstab 0.000500
F2 PROCESSING
lb 0.30
gf 0.070
gfs not used
fn 4096
F1 PROCESSING
lb1 0.32
gf1 0.009
gfs1 not used
procl lp
fn1 2048
DISPLAY
sp 430.8
wp 1195.2
sp1 2855.0
wp1 5158.8
rfl 128.4
rfp 0
rfl1 303.1
rfp1 0
PLOT
wc 250.0
sc 0
wc2 200.0
sc2 0
vs 751
th 2
ai cdc ph
ACQUISITION ARRAYS
array phase
arraydim 800
i phase
1 1
2 2
F2 (ppm)
1.0
1.2
1.4
1.6
1.8
2.0
2.2
2.4
2.6
50
45
40
35
30
25
20
F1 (ppm)

Figure S13 HMBC of 2

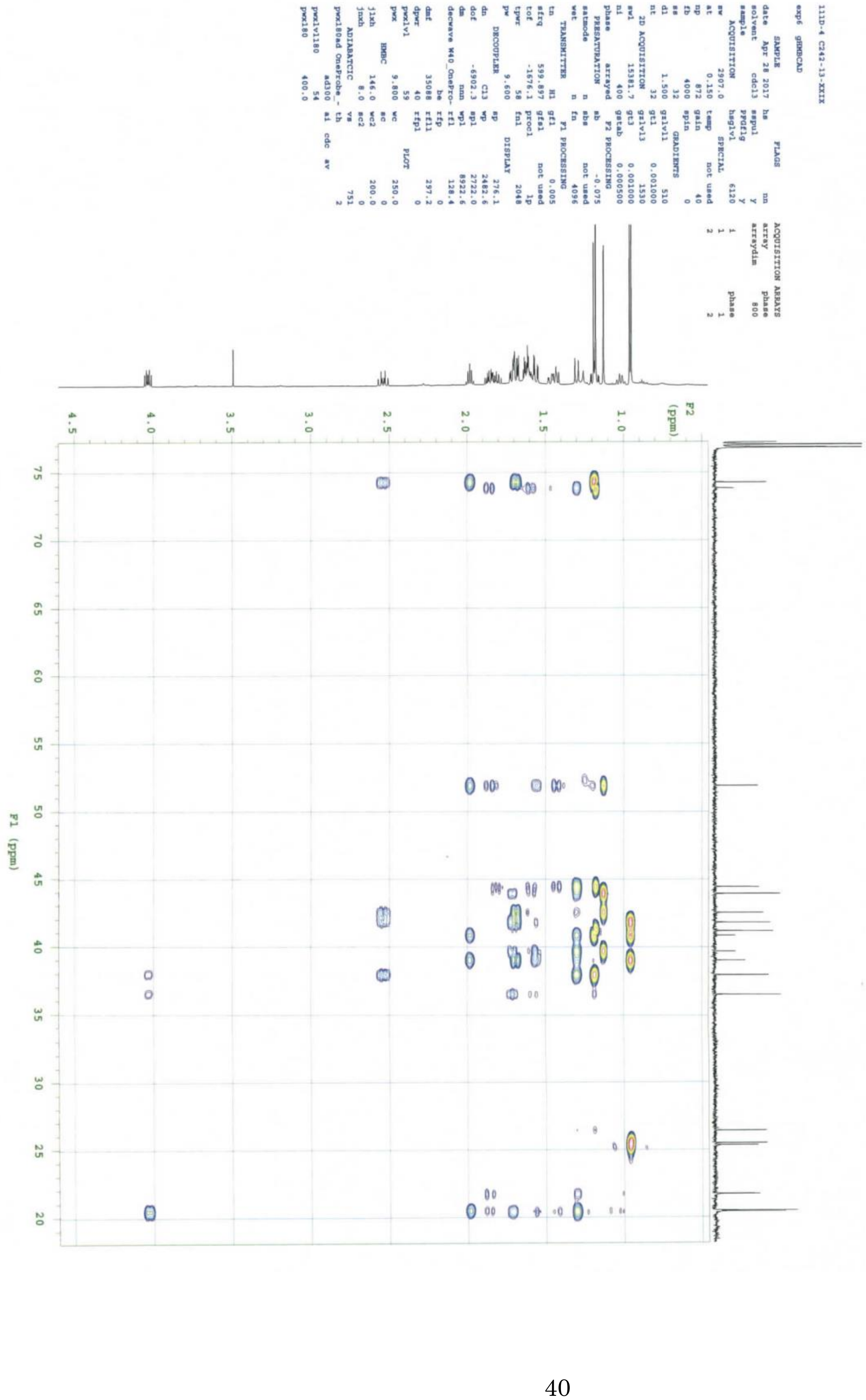

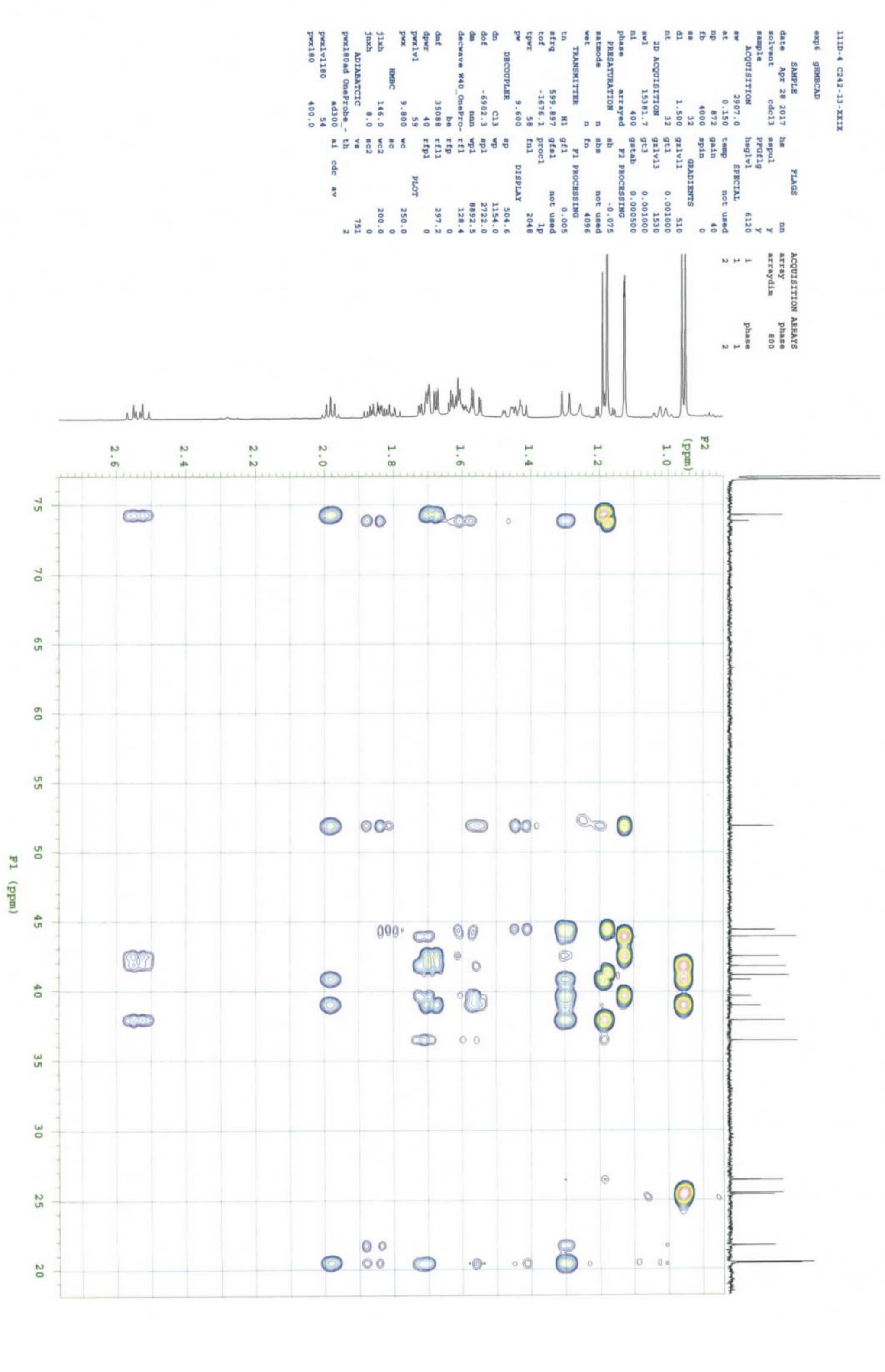
111D-4 C242-13-XXIX
exp6 gHMBCAD
F2 (ppm)
F1 (ppm)

Figure S14 FABMS of 2

```
[ Elemental Composition ]                                              Page: 1
Data  : 1705110                     Date : 17-May-2017 18:16
Sample: 111D-4 C242-13-XXIX
Note : Matrix; G
Inlet : Direct                      Ion Mode : FAB+
RT : 4.70 min                       Scan#: (26,31)
Elements : C 25/15, H 40/30, O 10/0
Mass Tolerance      : 20ppm, 1mmu if m/z > 50
Unsaturation (U.S.) : -1.0 - 30.0

Observed m/z Int%   Err[ppm / mmu]    U.S.  Composition
  289.2534    88.4     +0.9 /  +0.2    4.5  C 20 H 33 O
```
