# Supplementary material for: New Diterpenes with a Fused 6-5-6-6 Ring System Isolated from the Marine Sponge-Derived Fungus Trichoderma harzianum
Source: Mar Drugs. 2019 Aug 19;17(8):480. doi: 10.3390/md17080480 (PMC6722853; doi:10.3390/md17080480)

**Figure S15 $^{1}$H NMR spectrum of 3 in $CDCl_3$**

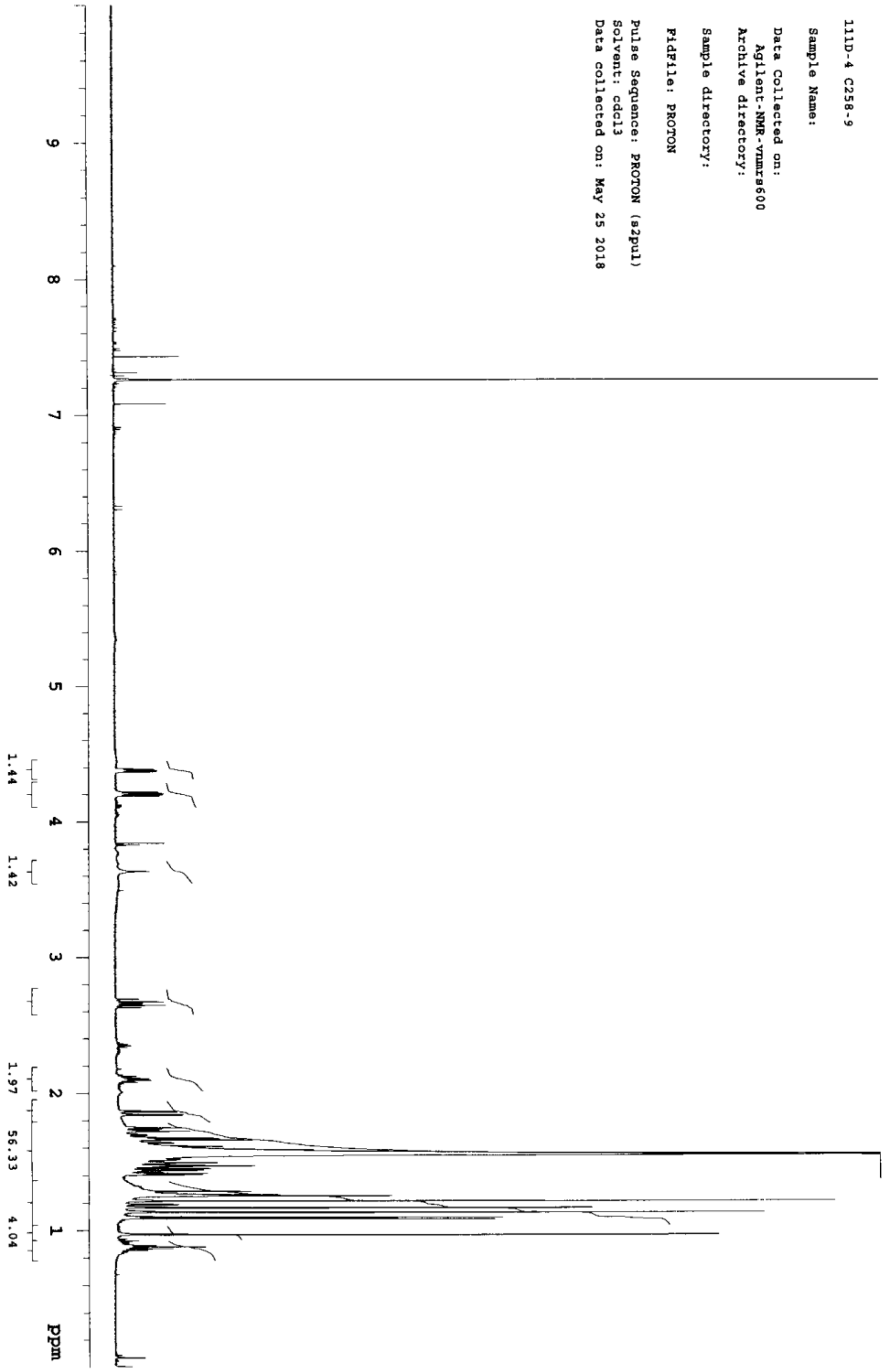

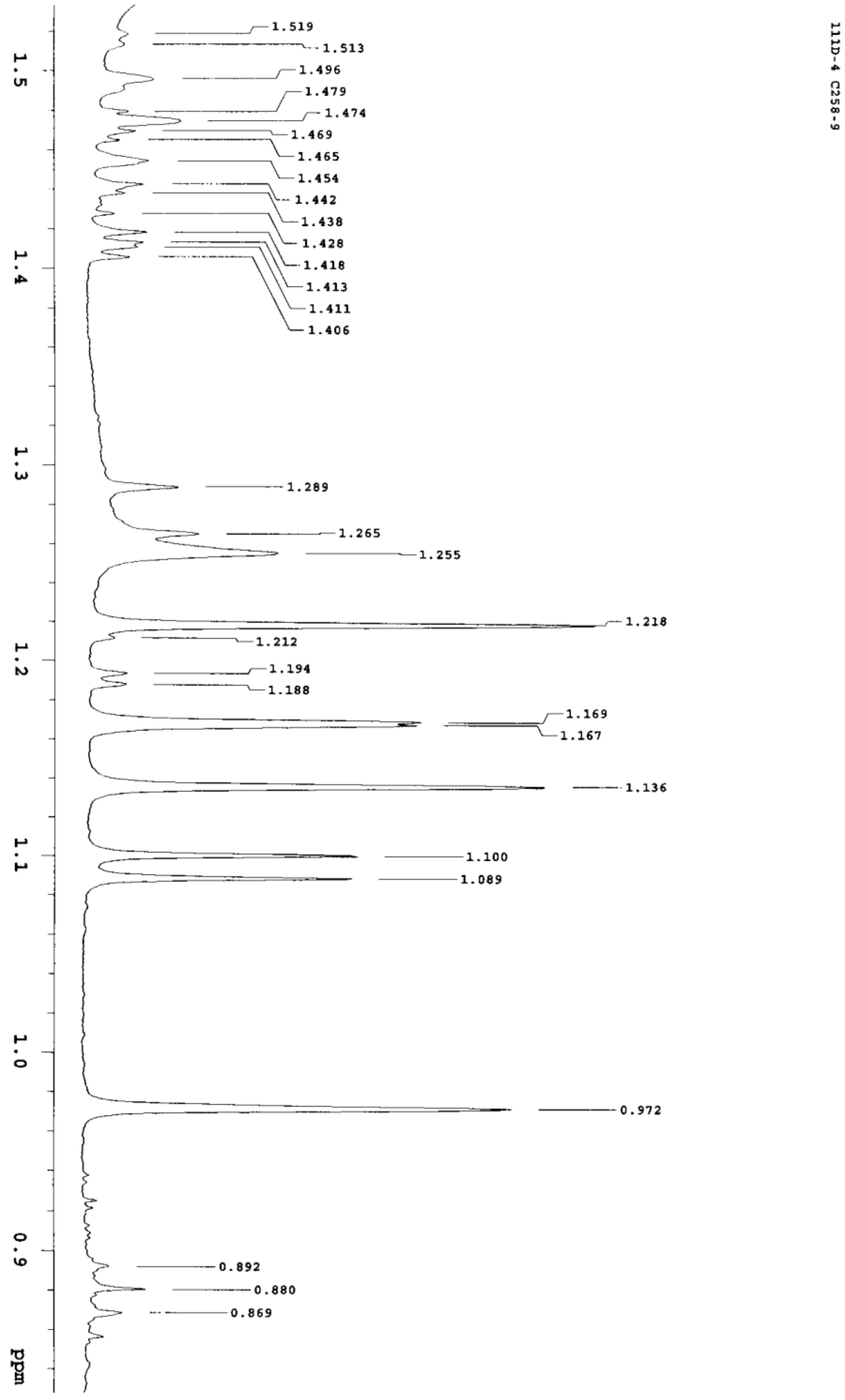
111D-4 C258-9
1.519
1.513
1.496
1.479
1.474
1.469
1.465
1.454
1.442
1.438
1.428
1.418
1.413
1.411
1.406
1.289
1.265
1.255
1.218
1.212
1.194
1.188
1.169
1.167
1.136
1.100
1.089
0.972
0.892
0.880
0.869
1.5
1.4
1.3
1.2
1.1
1.0
0.9
ppm

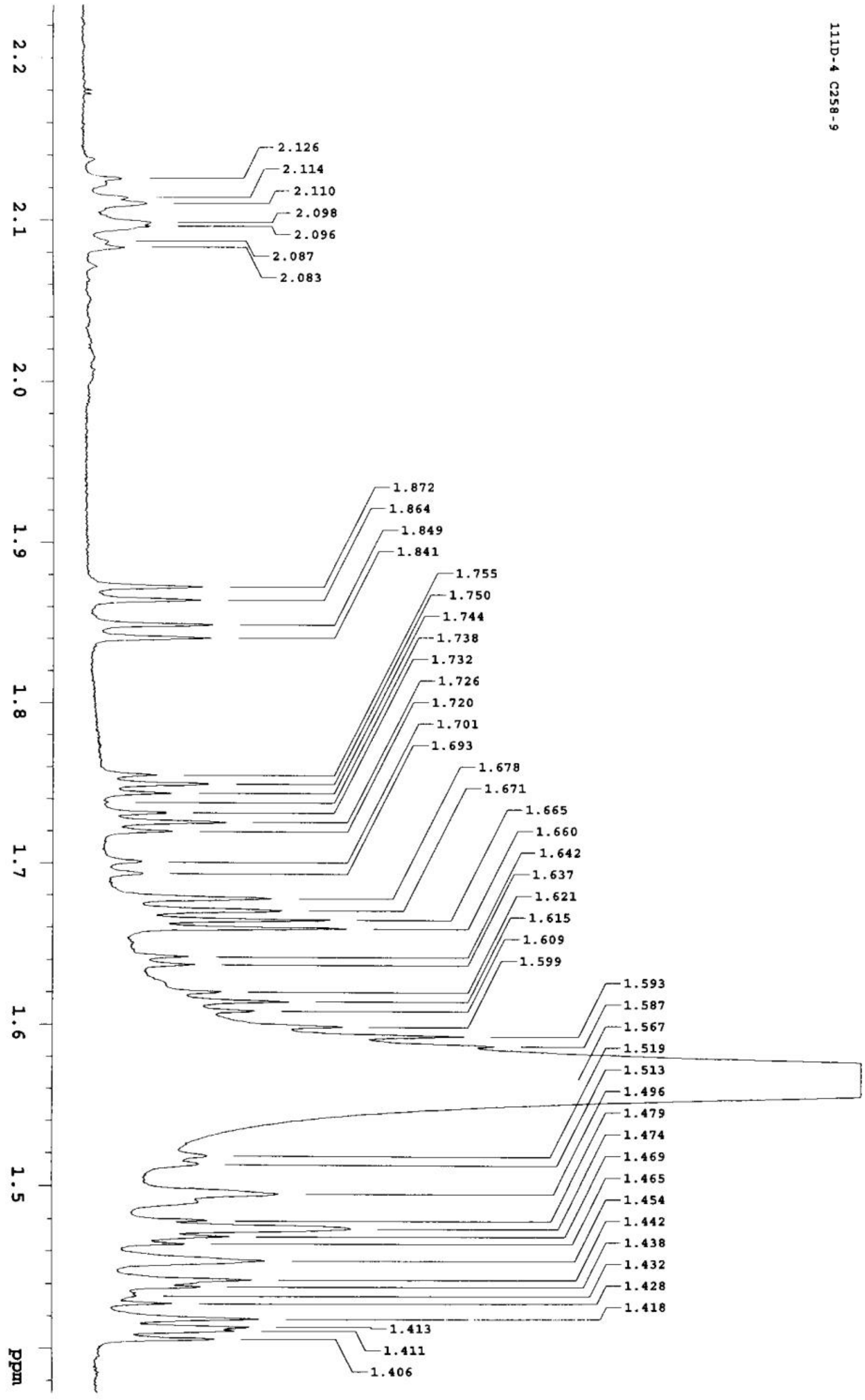
111D-4 C258-9
2.2
2.1
2.0
1.9
1.8
1.7
1.6
1.5
ppm
2.126
2.114
2.110
2.098
2.096
2.087
2.083
1.872
1.864
1.849
1.841
1.755
1.750
1.744
1.738
1.732
1.726
1.720
1.701
1.693
1.678
1.671
1.665
1.660
1.642
1.637
1.621
1.615
1.609
1.599
1.593
1.587
1.567
1.519
1.513
1.496
1.479
1.474
1.469
1.465
1.454
1.442
1.438
1.432
1.428
1.418
1.413
1.411
1.406

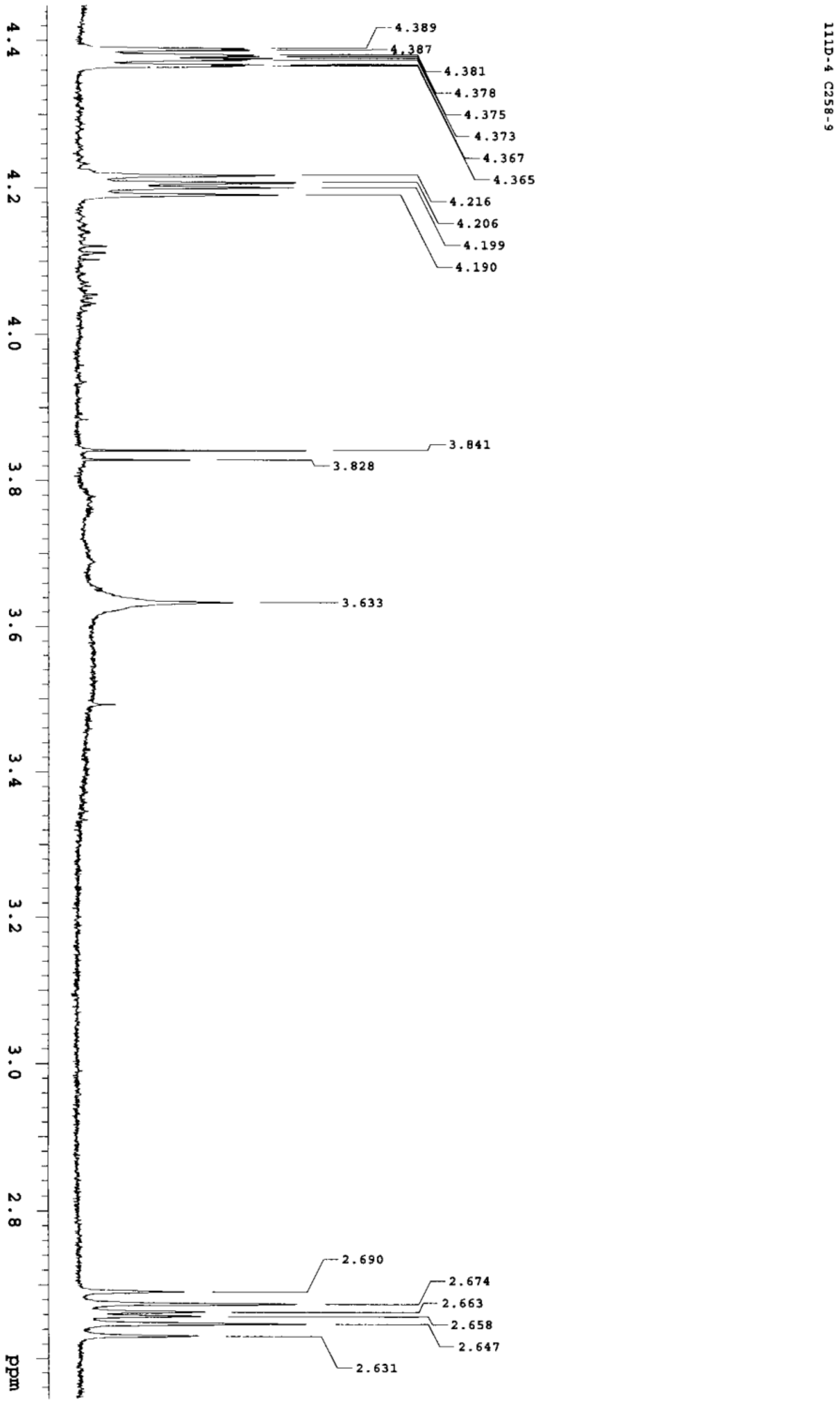
111D-4 C258-9
4.389
4.387
4.381
4.378
4.375
4.373
4.367
4.365
4.216
4.206
4.199
4.190
3.841
3.828
3.633
2.690
2.674
2.663
2.658
2.647
2.631
4.4
4.2
4.0
3.8
3.6
3.4
3.2
3.0
2.8
ppm

**Figure S16 $^{13}$C NMR spectrum of 3 in $CDCl_3$**

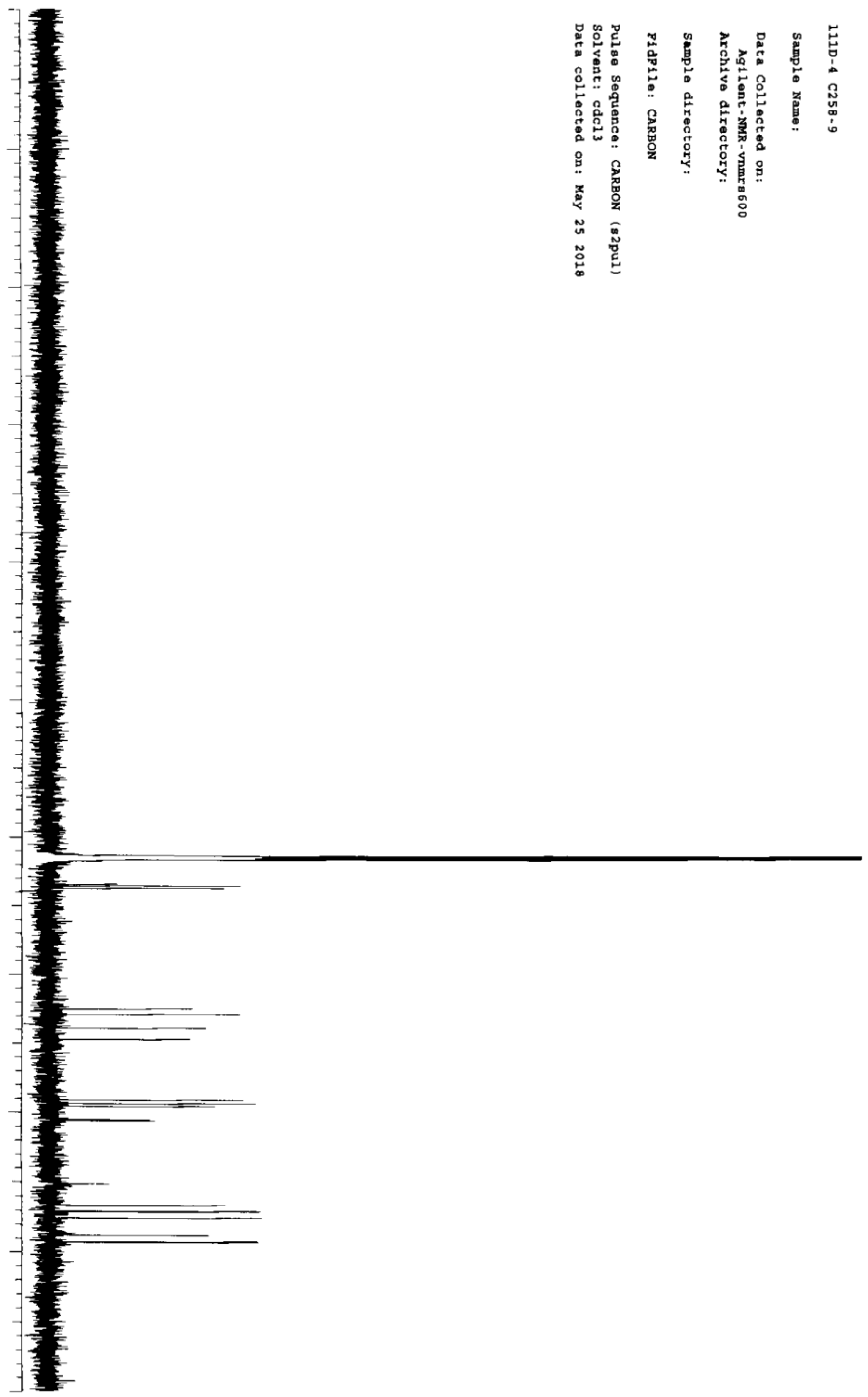

111D-4 C258-9

| INDEX | FREQUENCY | PPM | HEIGHT |
|---|---|---|---|
| 1 | 11647.3 | 77.215 | 3007.5 |
| 2 | 11615.0 | 77.000 | 3101.7 |
| 3 | 11583.8 | 76.793 | 2743.4 |
| 4 | 11031.2 | 73.130 | 13.2 |
| 5 | 10996.5 | 72.900 | 36.5 |
| 6 | 10950.3 | 72.594 | 33.4 |
| 7 | 8299.7 | 55.022 | 27.5 |
| 8 | 8179.4 | 54.225 | 36.5 |
| 9 | 7874.3 | 52.201 | 30.0 |
| 10 | 7638.4 | 50.638 | 26.9 |
| 11 | 6309.1 | 41.825 | 37.1 |
| 12 | 6230.5 | 41.304 | 39.4 |
| 13 | 6177.3 | 40.952 | 31.7 |
| 14 | 5886.0 | 39.020 | 19.2 |
| 15 | 5862.9 | 38.867 | 18.0 |
| 16 | 5858.3 | 38.837 | 20.5 |
| 17 | 4479.2 | 29.694 | 11.6 |
| 18 | 4026.0 | 26.690 | 33.6 |
| 19 | 3905.8 | 25.893 | 28.6 |
| 20 | 3890.8 | 25.794 | 40.3 |
| 21 | 3754.4 | 24.889 | 40.5 |
| 22 | 3379.9 | 22.406 | 30.6 |
| 23 | 3240.0 | 21.479 | 39.6 |
| 24 | 3221.5 | 21.356 | 40.0 |

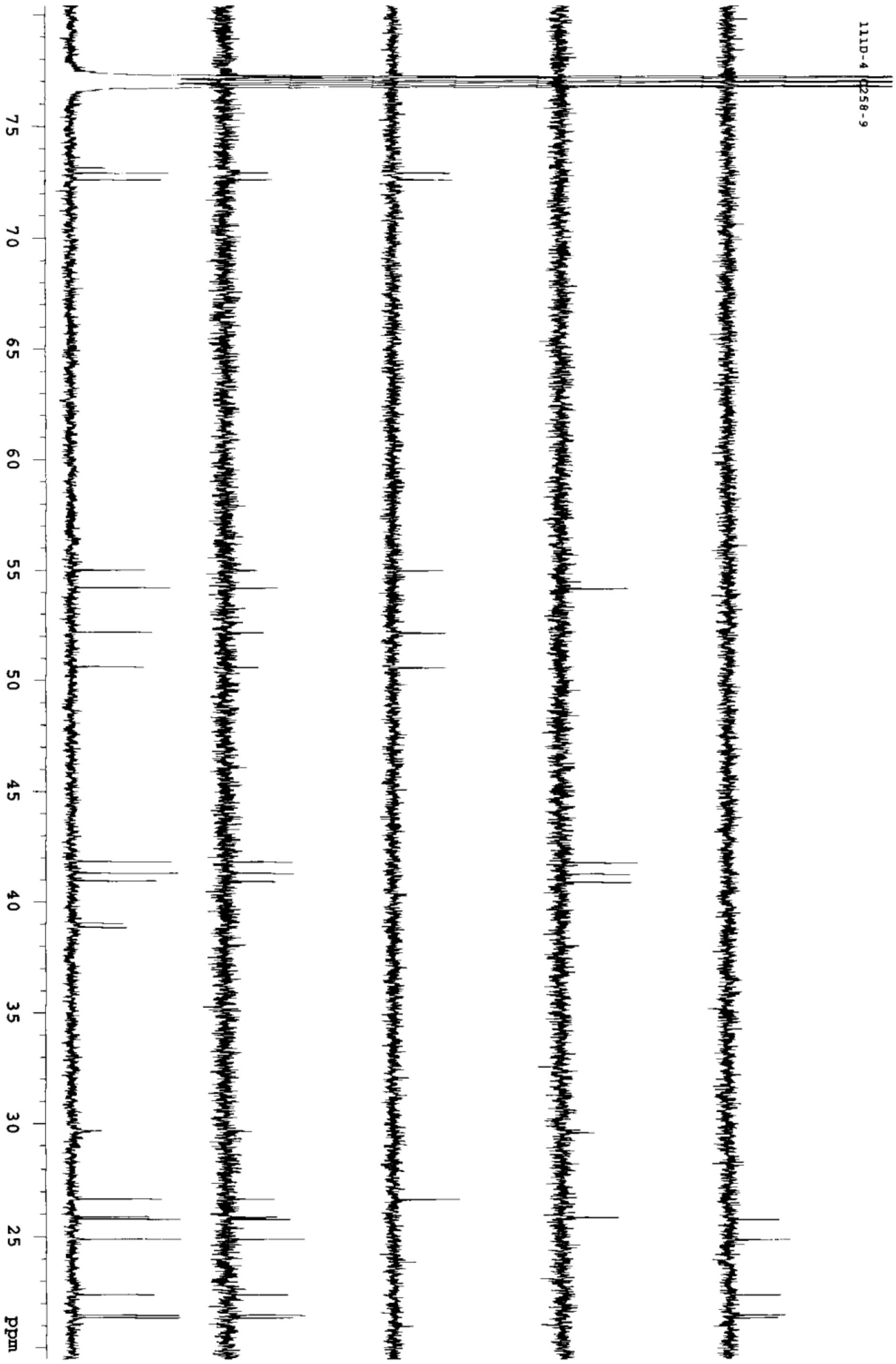
111D-4 C258-9
75
70
65
60
55
50
45
40
35
30
25
ppm

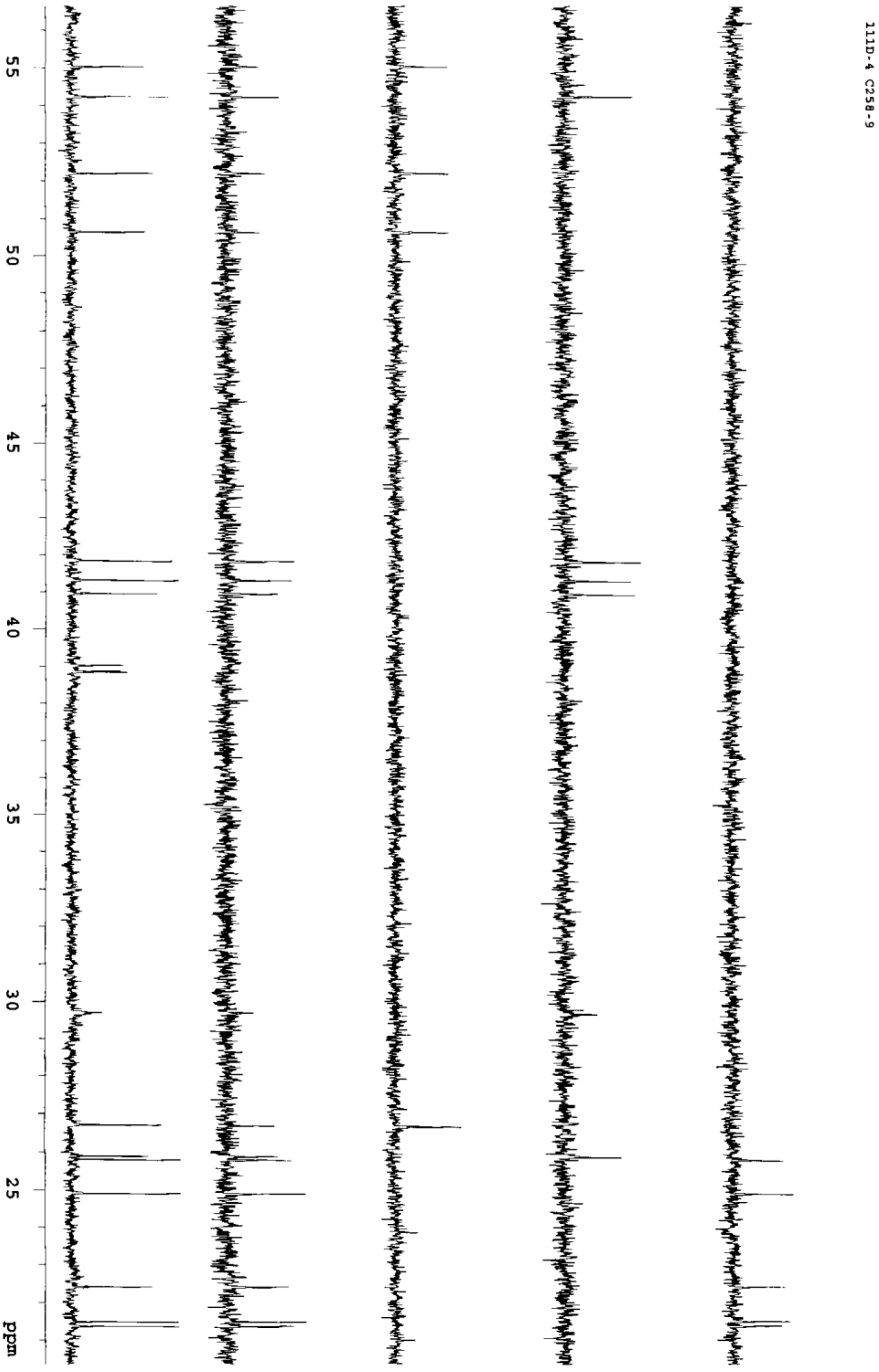
111D-4 C258-9
55
50
45
40
35
30
25
ppm

**Figure S17 $^{1}$H-$^{1}$H COSY of 3**

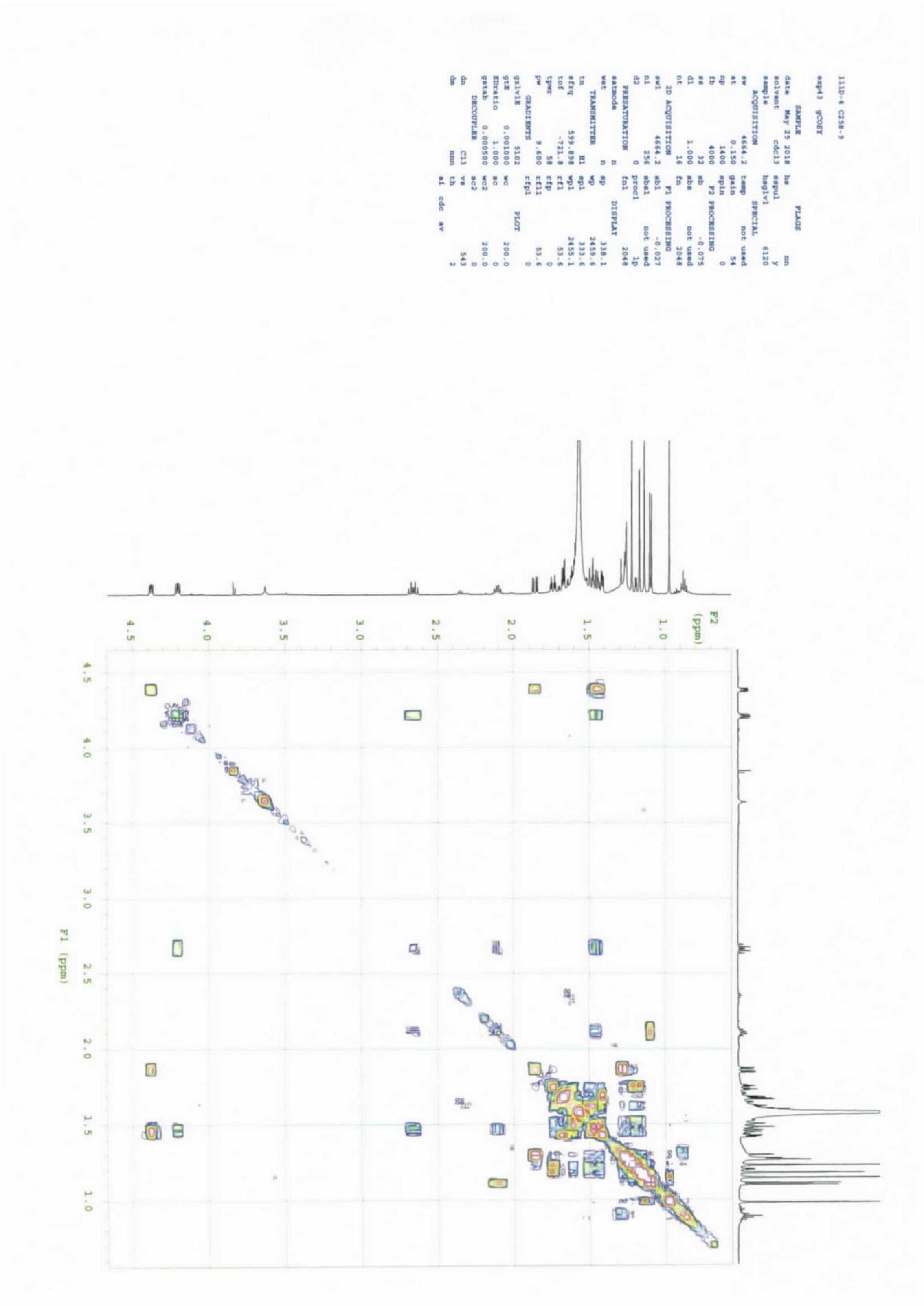

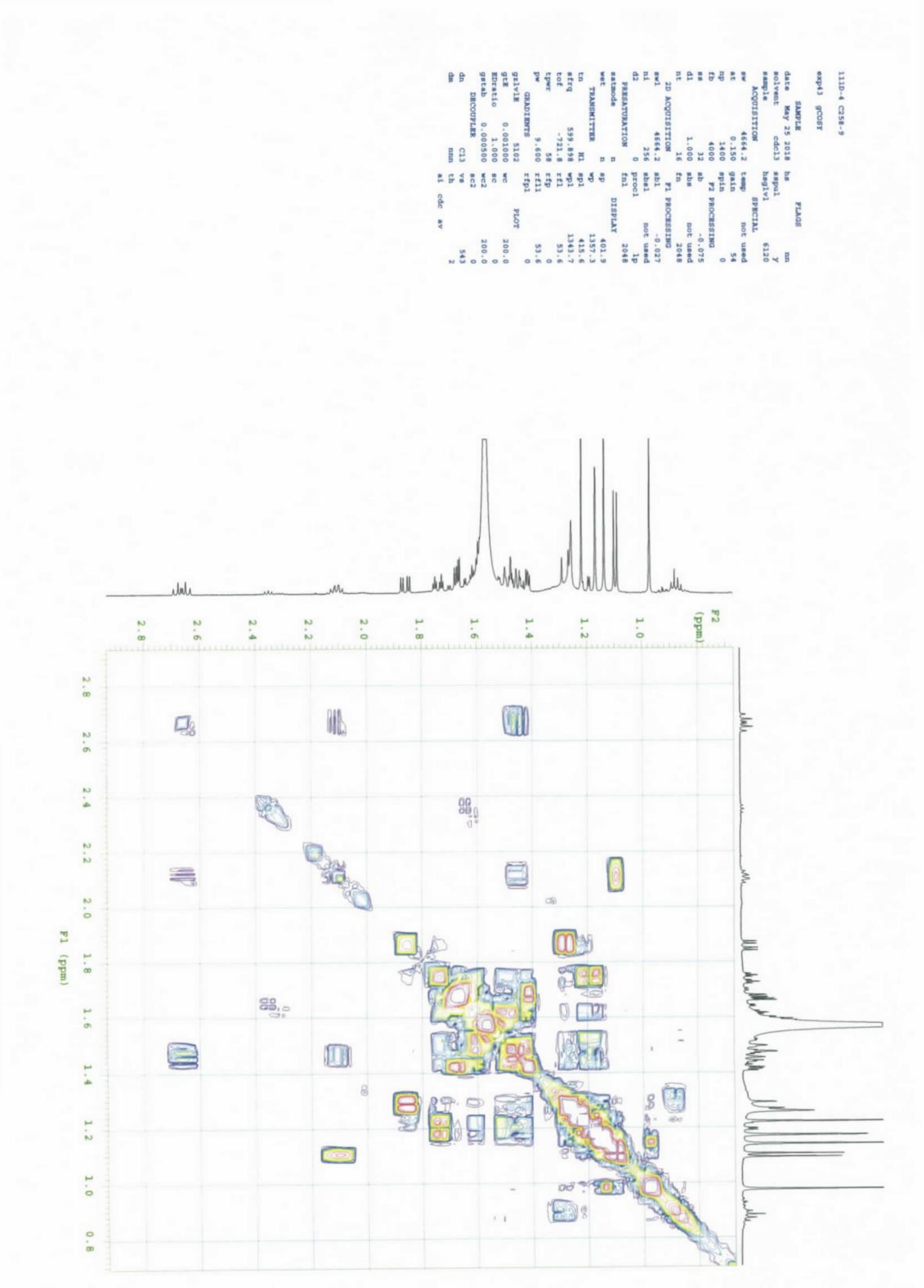

F2 (ppm)
F1 (ppm)

**Figure S18 NOESY of 3**

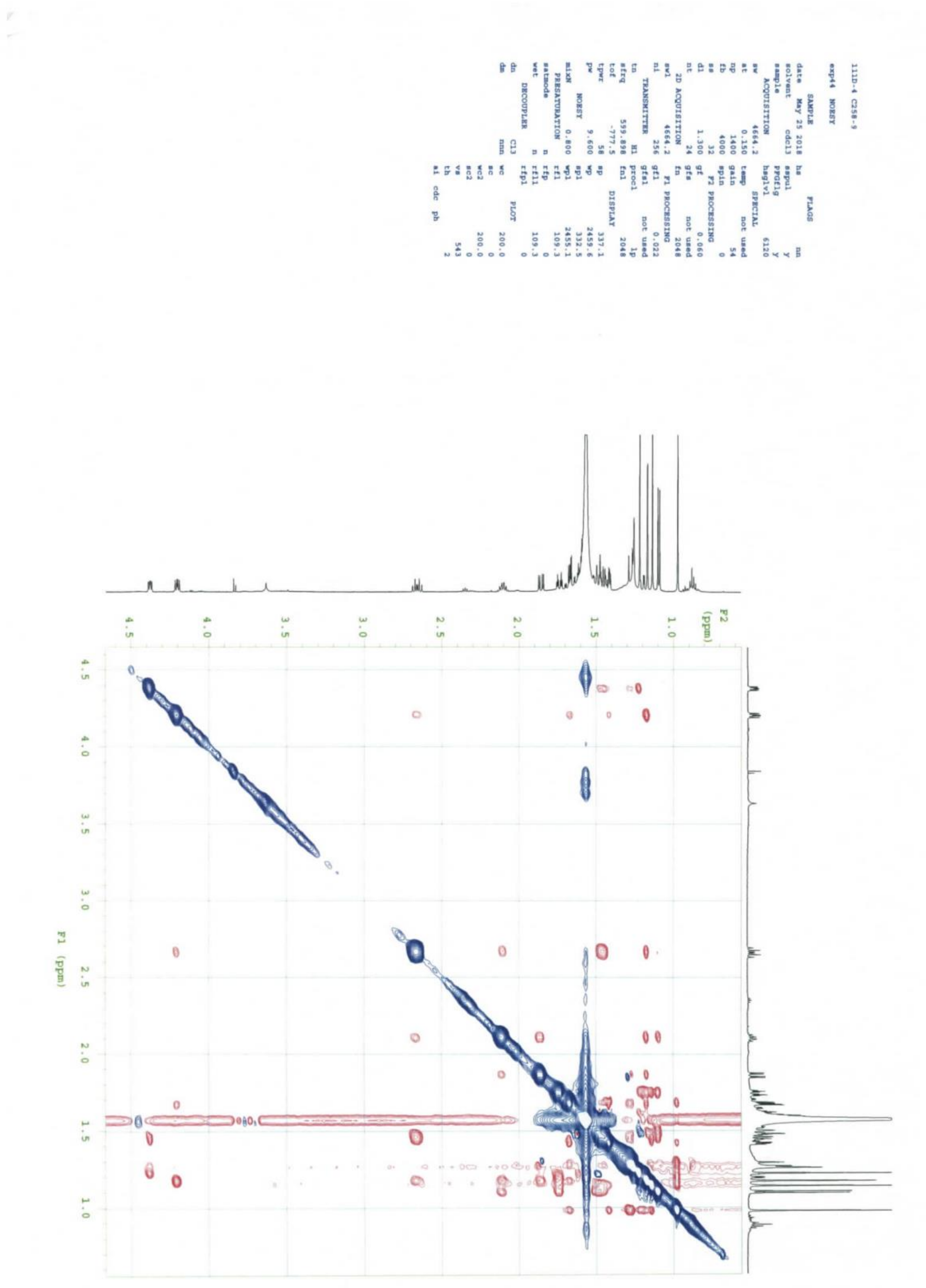

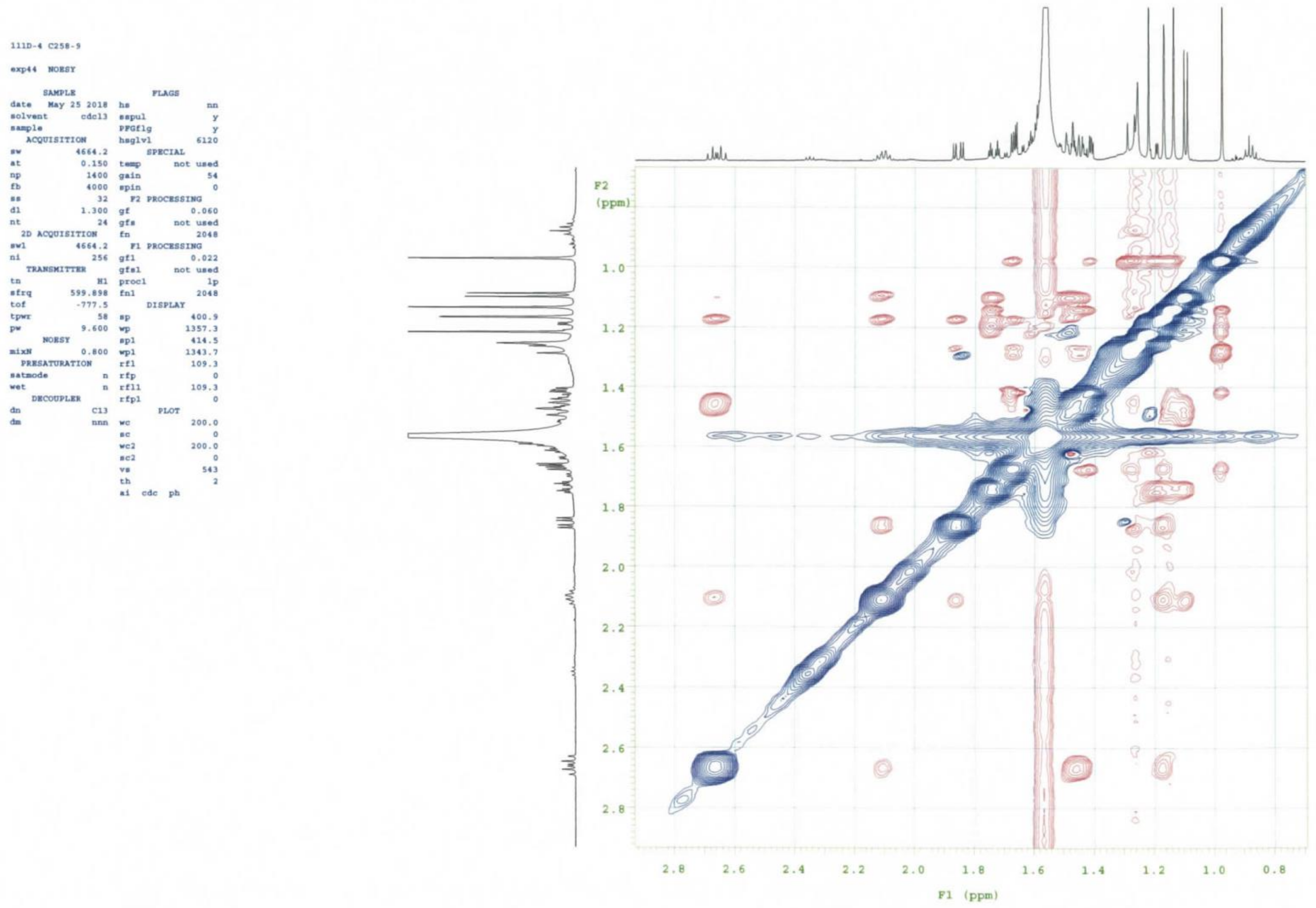
111D-4 C258-9
exp44 NOESY
SAMPLE
date May 25 2018
solvent cdcl3
sample
ACQUISITION
sw 4664.2
at 0.150
np 1400
fb 4000
ss 32
d1 1.300
nt 24
2D ACQUISITION
sw1 4664.2
ni 256
TRANSMITTER
tn H1
sfrq 599.898
tof -777.5
tpwr 58
pw 9.600
NOESY
mixN 0.800
PRESATURATION
satmode n
wet n
DECOUPLER
dn C13
dm nnn
FLAGS
hs nn
sspul y
PFGflg y
hsglvl 6120
SPECIAL
temp not used
gain 54
spin 0
F2 PROCESSING
gf 0.060
gfs not used
fn 2048
F1 PROCESSING
gf1 0.022
gfs1 not used
procl lp
fn1 2048
DISPLAY
sp 400.9
wp 1357.3
sp1 414.5
wp1 1343.7
rfl 109.3
rfp 0
rfl1 109.3
rfp1 0
PLOT
wc 200.0
sc 0
wc2 200.0
sc2 0
vs 543
th 2
ai cdc ph
F2 (ppm)
F1 (ppm)

**Figure S19 HMQC of 3**

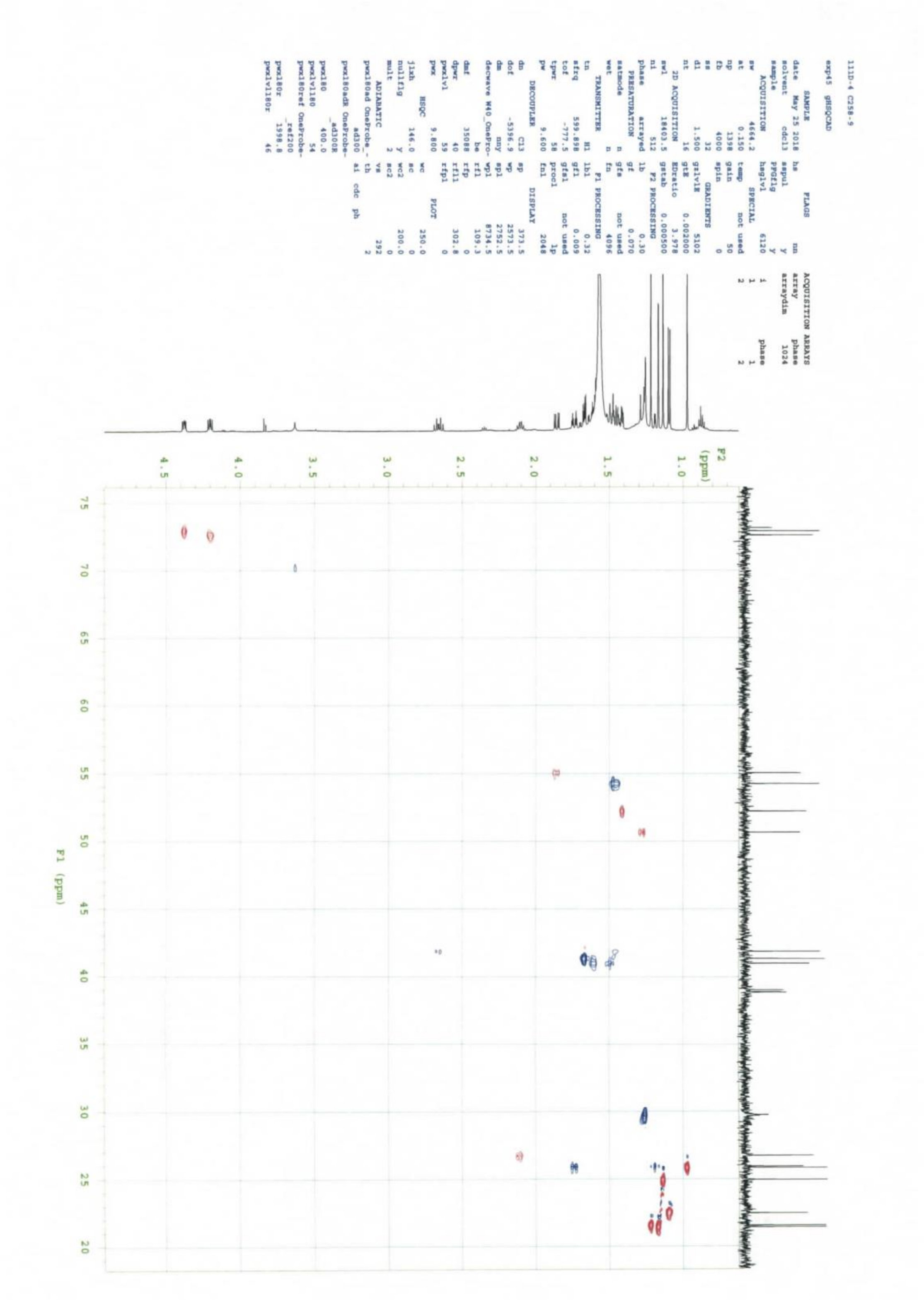

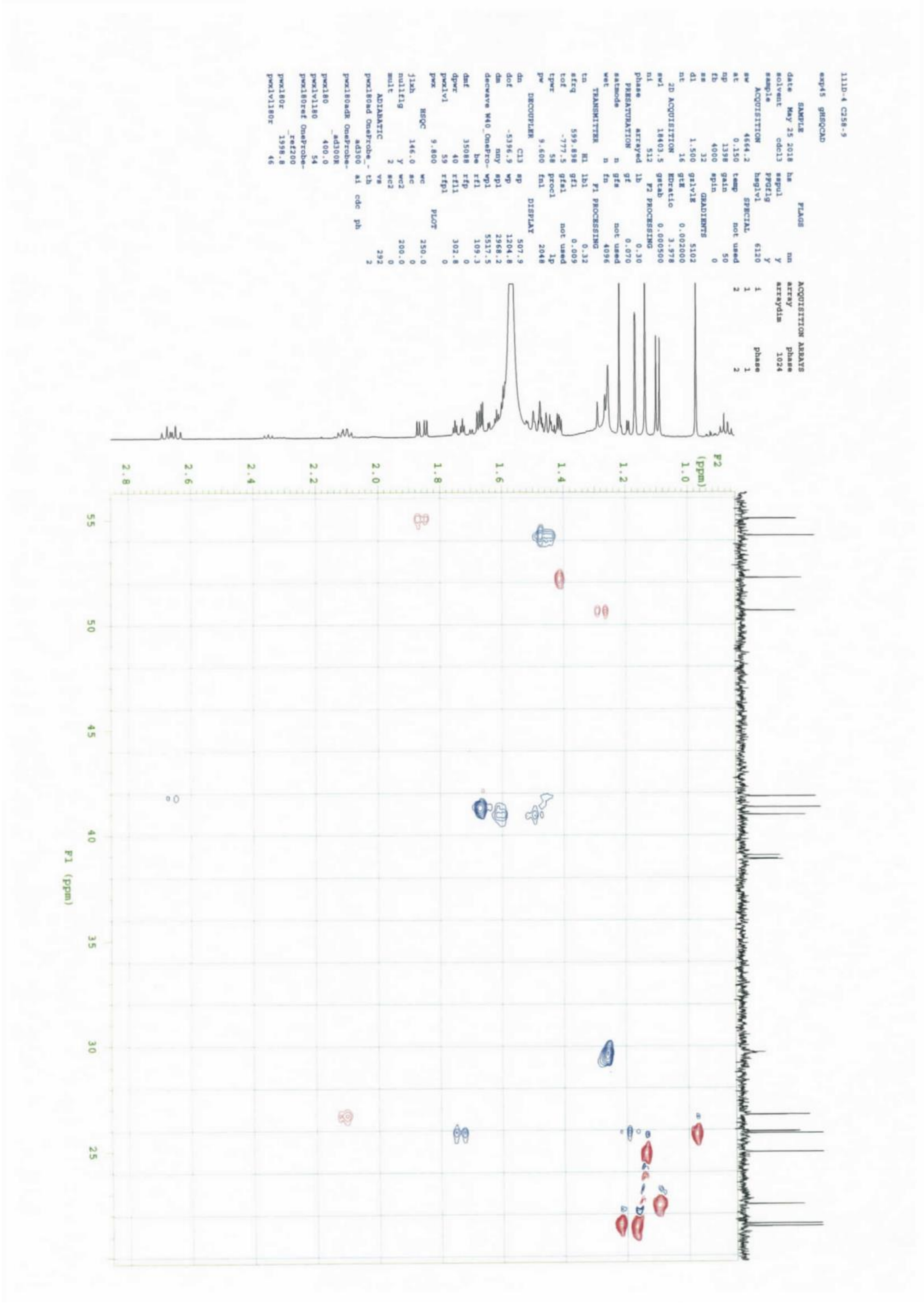
F2 (ppm)
F1 (ppm)

**Figure S20    HMBC of 3**

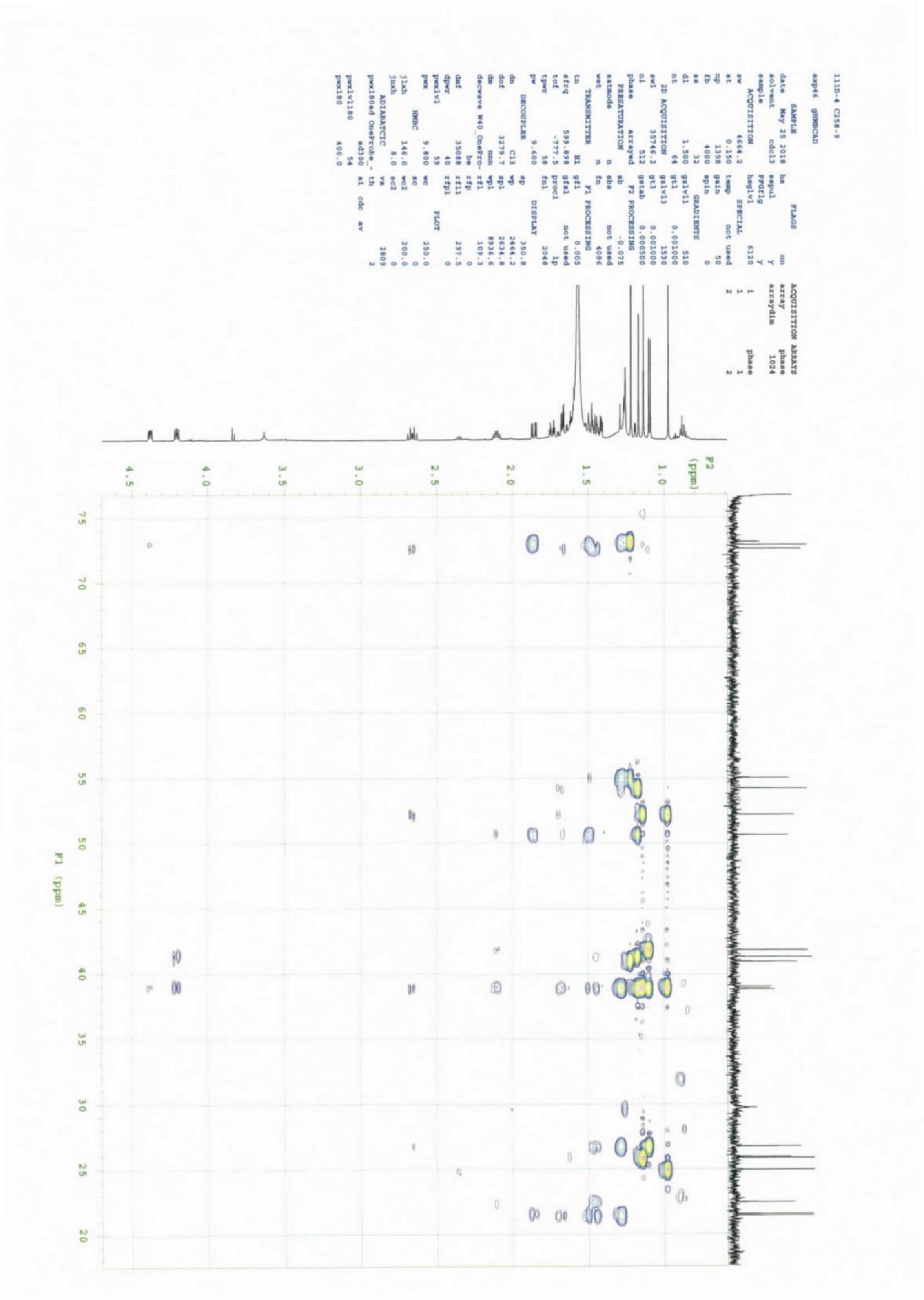

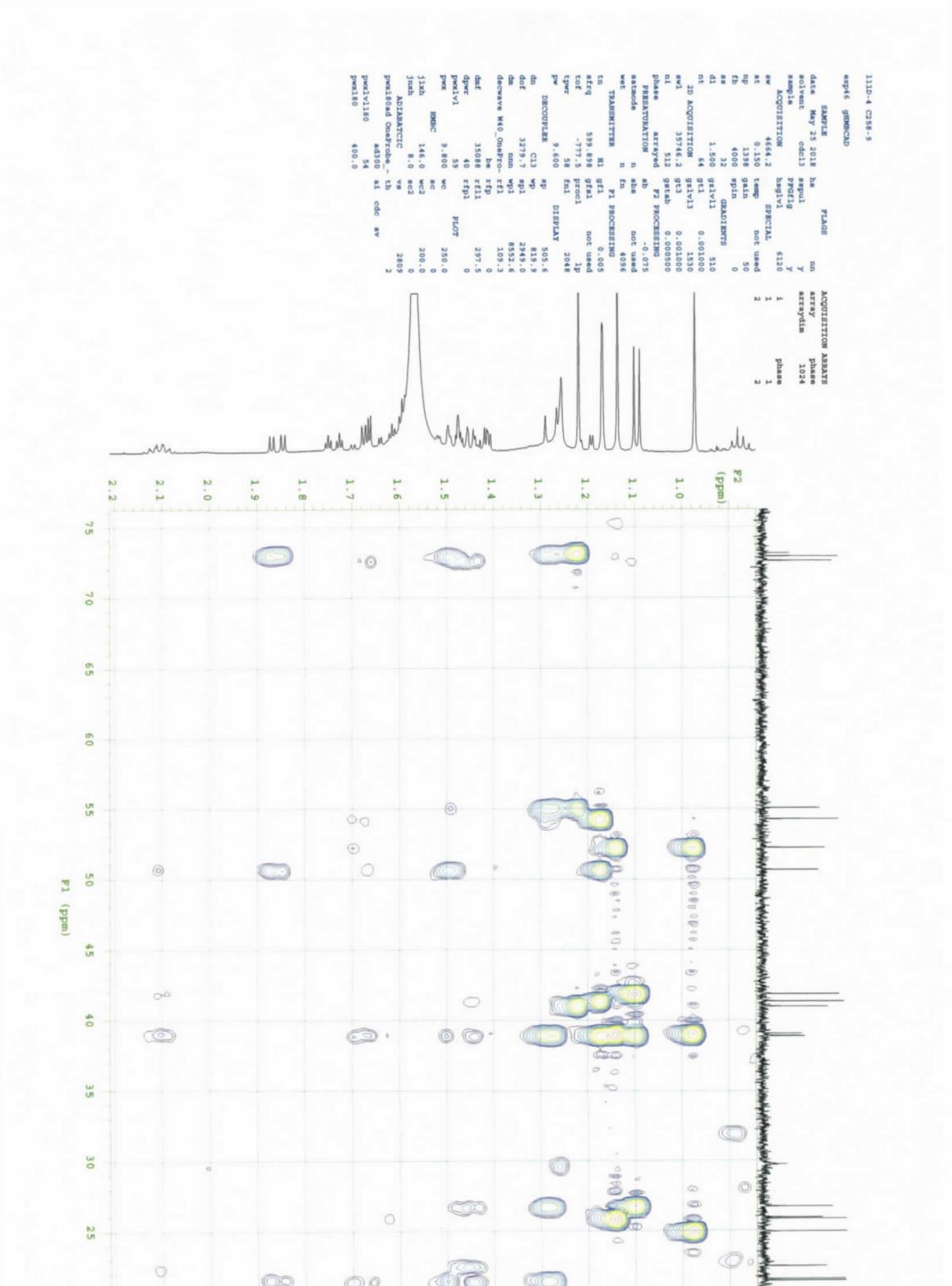
F2 (ppm)
F1 (ppm)

**igure S21 FABMS of 3**

[ Elemental Composition ] 高分解能 Page: 1

Data : 1807068 Date : 12-Jul-2018 14:41

Sample: 111D-4 C258-9

Note : Matrix; G

Inlet : Direct Ion Mode : FAB+

RT : 5.41 min Scan#: (35,40)

Elements : C 25/15, H 40/30, O 5/0, Na 1/0

Mass Tolerance : 20ppm, 2mmu if m/z > 100

Unsaturation (U.S.) : -1.0 - 40.0

| Observed m/z | Int% | Err[ppm / mmu] | U.S. | Composition |
|---|---|---|---|---|
| 345.2398 | 100.0 | -2.3 / -0.8 | 3.5 | C 20 H 34 O 3 Na |

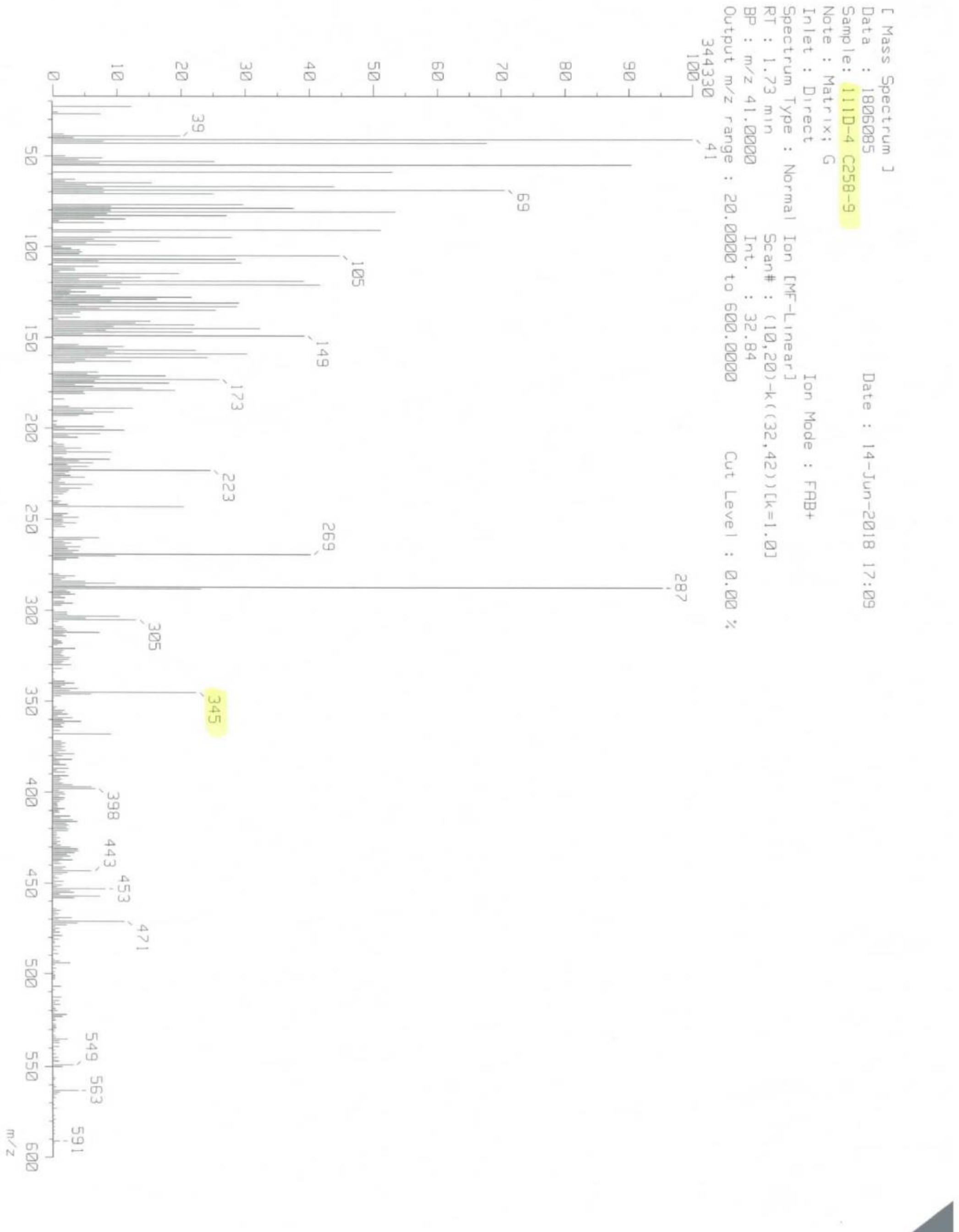
[ Mass Spectrum ]
Data : 1806085
Date : 14-Jun-2018 17:09
Sample: 111D-4 C258-9
Note : Matrix; G
Inlet : Direct
Ion Mode : FAB+
Spectrum Type : Normal Ion [MF-Linear]
RT : 1.73 min
Scan# : (10,20)-k(32,42))[k=1.0]
BP : m/z 41.0000
Int. : 32.84
Output m/z range : 20.0000 to 600.0000
Cut Level : 0.00 %
344330
39
41
69
105
149
173
223
269
287
305
345
398
443
453
471
549
563
591
m/z

**Figure S22 $^{1}$H NMR spectrum of 4 in CDCl$_3$**

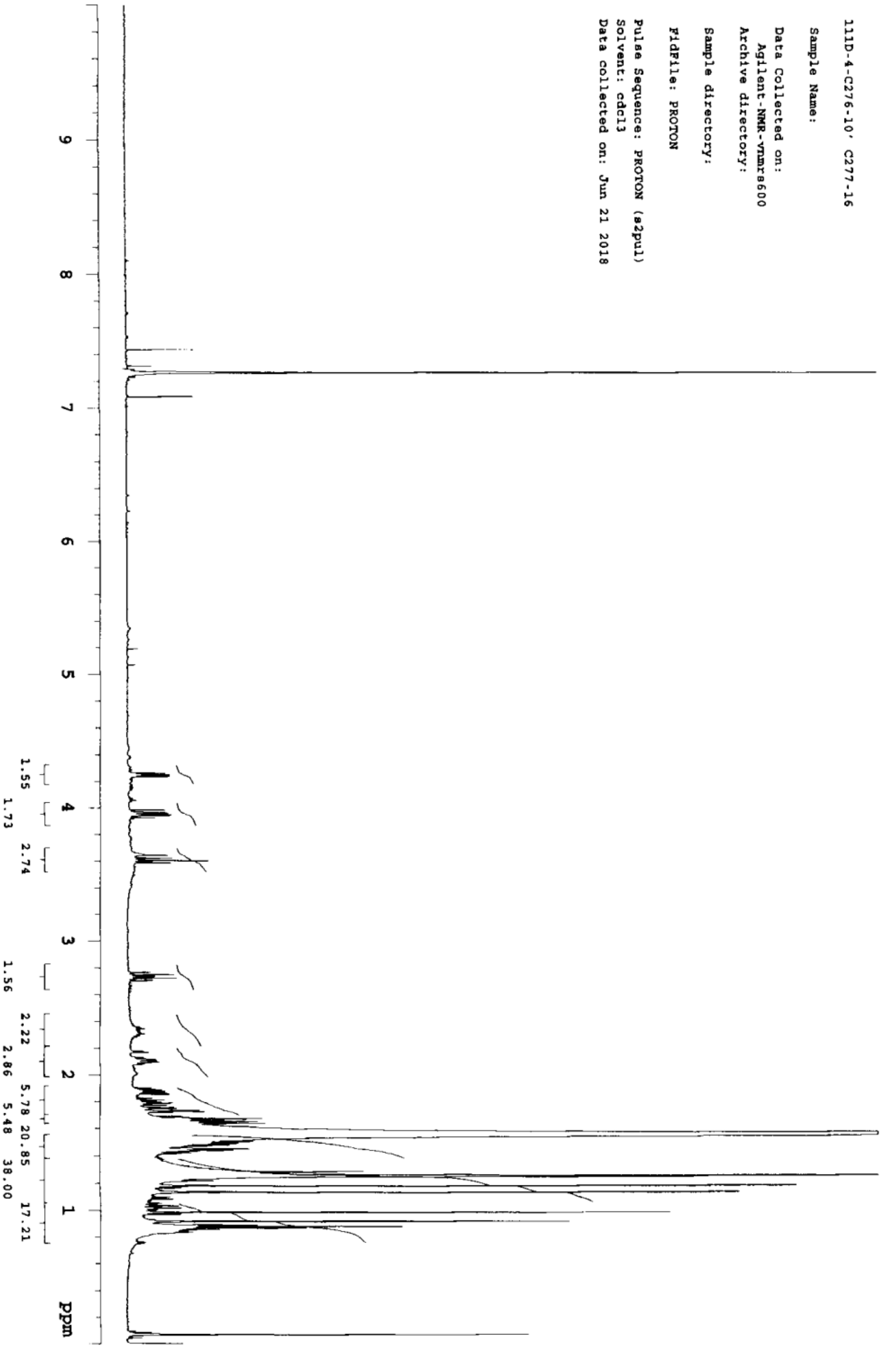

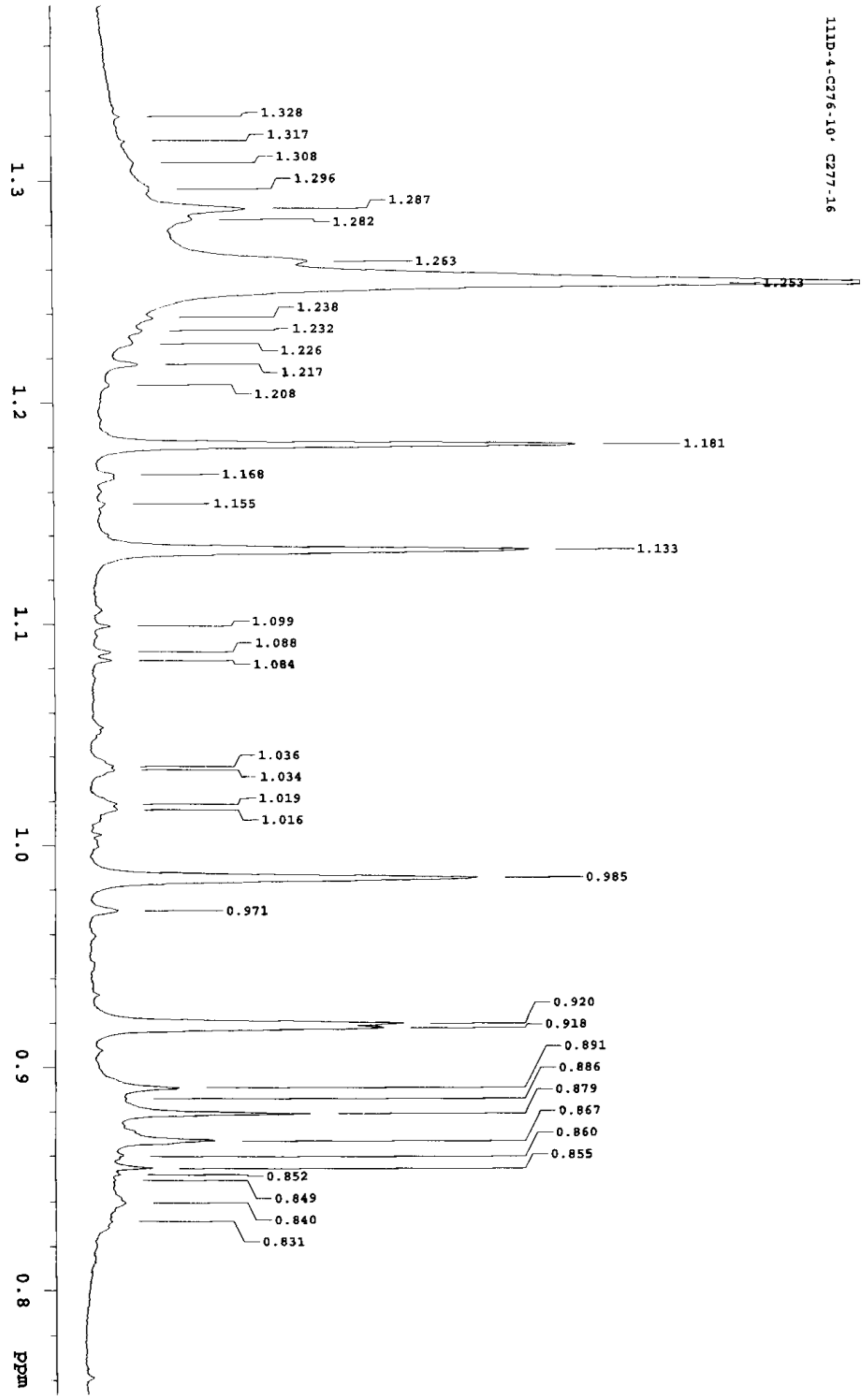
111D-4-C276-10' C277-16
1.328
1.317
1.308
1.296
1.287
1.282
1.263
1.253
1.238
1.232
1.226
1.217
1.208
1.181
1.168
1.155
1.133
1.099
1.088
1.084
1.036
1.034
1.019
1.016
0.985
0.971
0.920
0.918
0.891
0.886
0.879
0.867
0.860
0.855
0.852
0.849
0.840
0.831
1.3
1.2
1.1
1.0
0.9
0.8
ppm

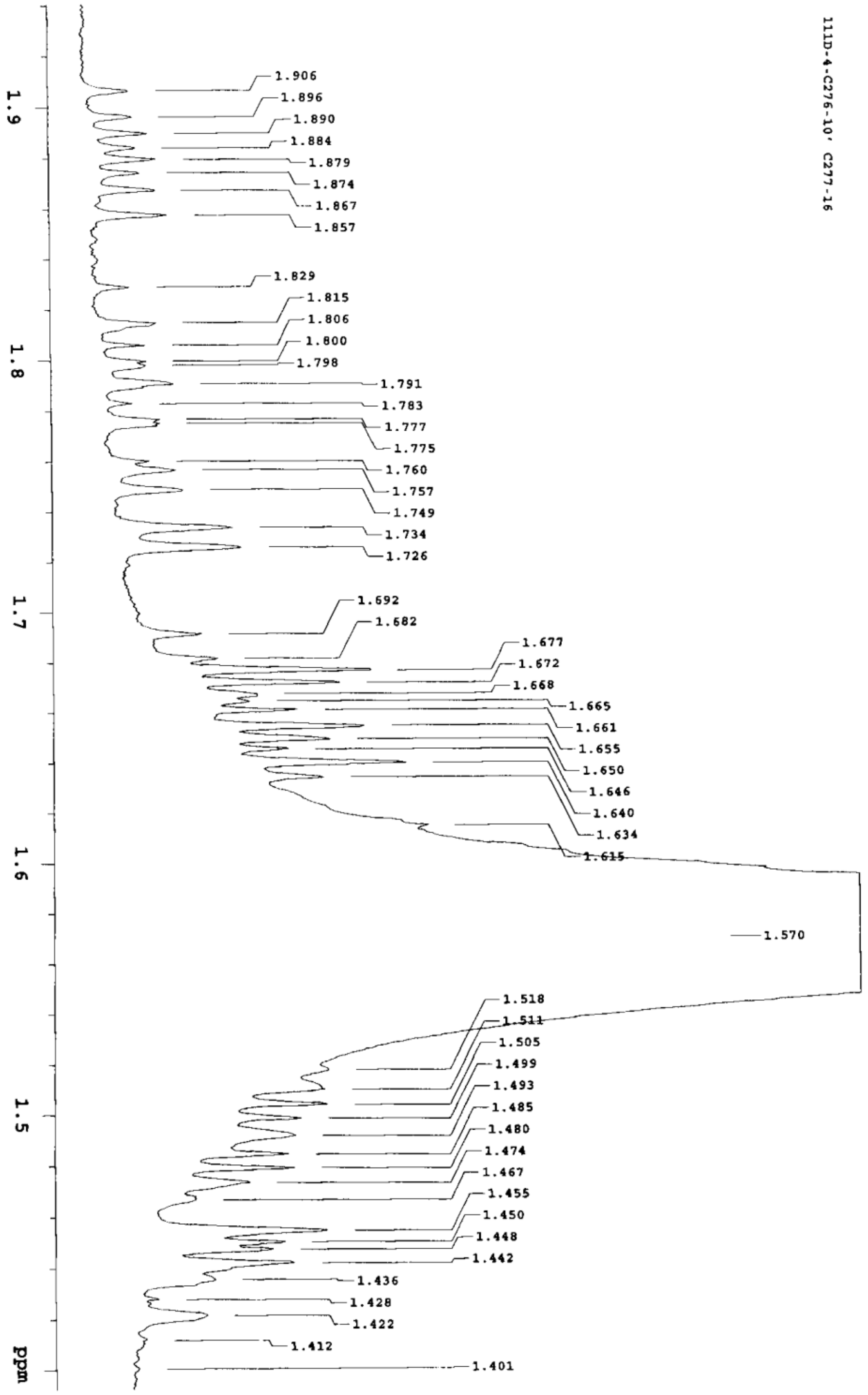
111D-4-C276-10' C277-16
1.906
1.896
1.890
1.884
1.879
1.874
1.867
1.857
1.829
1.815
1.806
1.800
1.798
1.791
1.783
1.777
1.775
1.760
1.757
1.749
1.734
1.726
1.692
1.682
1.677
1.672
1.668
1.665
1.661
1.655
1.650
1.646
1.640
1.634
1.615
1.570
1.518
1.511
1.505
1.499
1.493
1.485
1.480
1.474
1.467
1.455
1.450
1.448
1.442
1.436
1.428
1.422
1.412
1.401
1.9
1.8
1.7
1.6
1.5
ppm

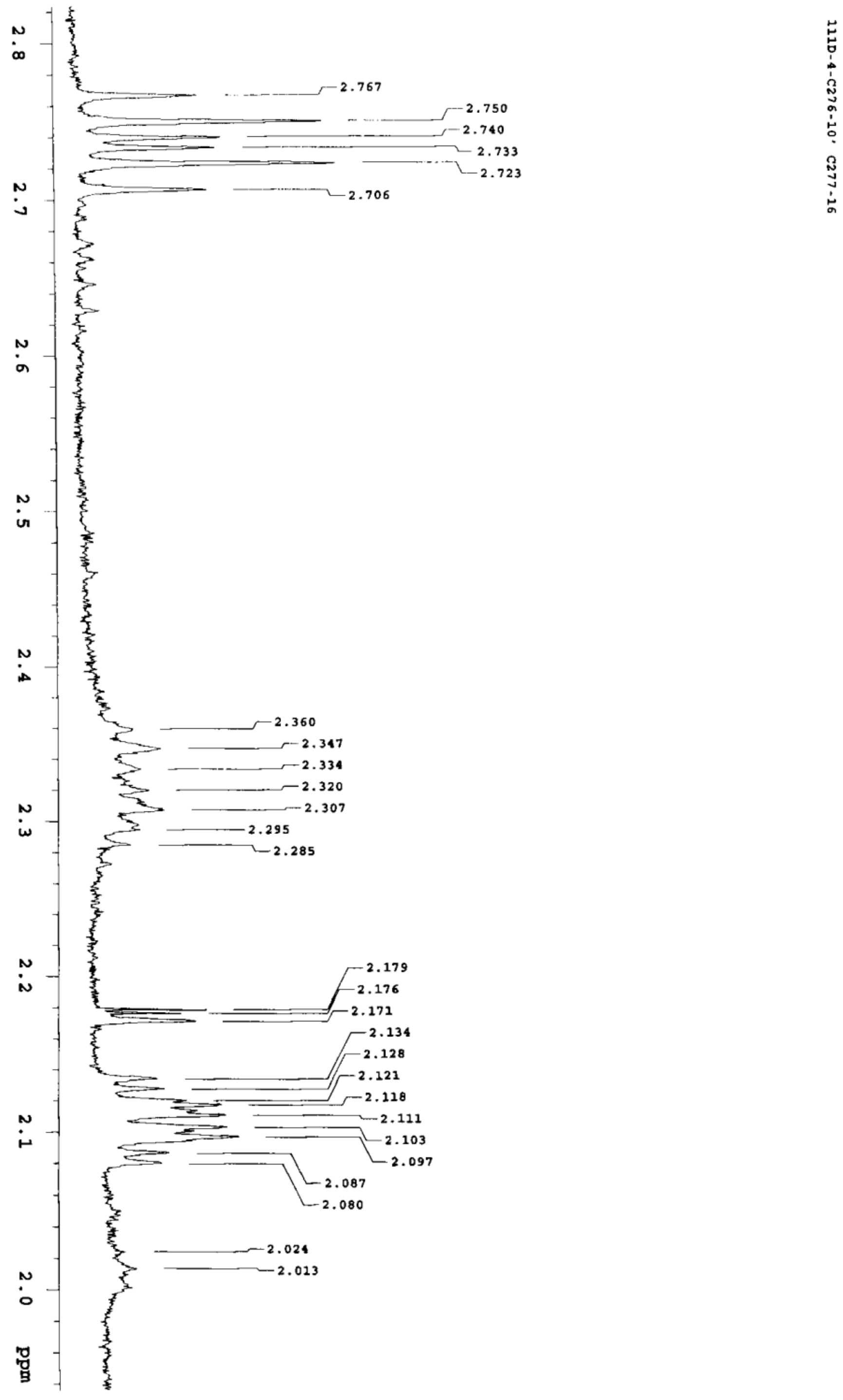
111D-4-C276-10' C277-16
2.767
2.750
2.740
2.733
2.723
2.706
2.360
2.347
2.334
2.320
2.307
2.295
2.285
2.179
2.176
2.171
2.134
2.128
2.121
2.118
2.111
2.103
2.097
2.087
2.080
2.024
2.013
2.8
2.7
2.6
2.5
2.4
2.3
2.2
2.1
2.0
ppm

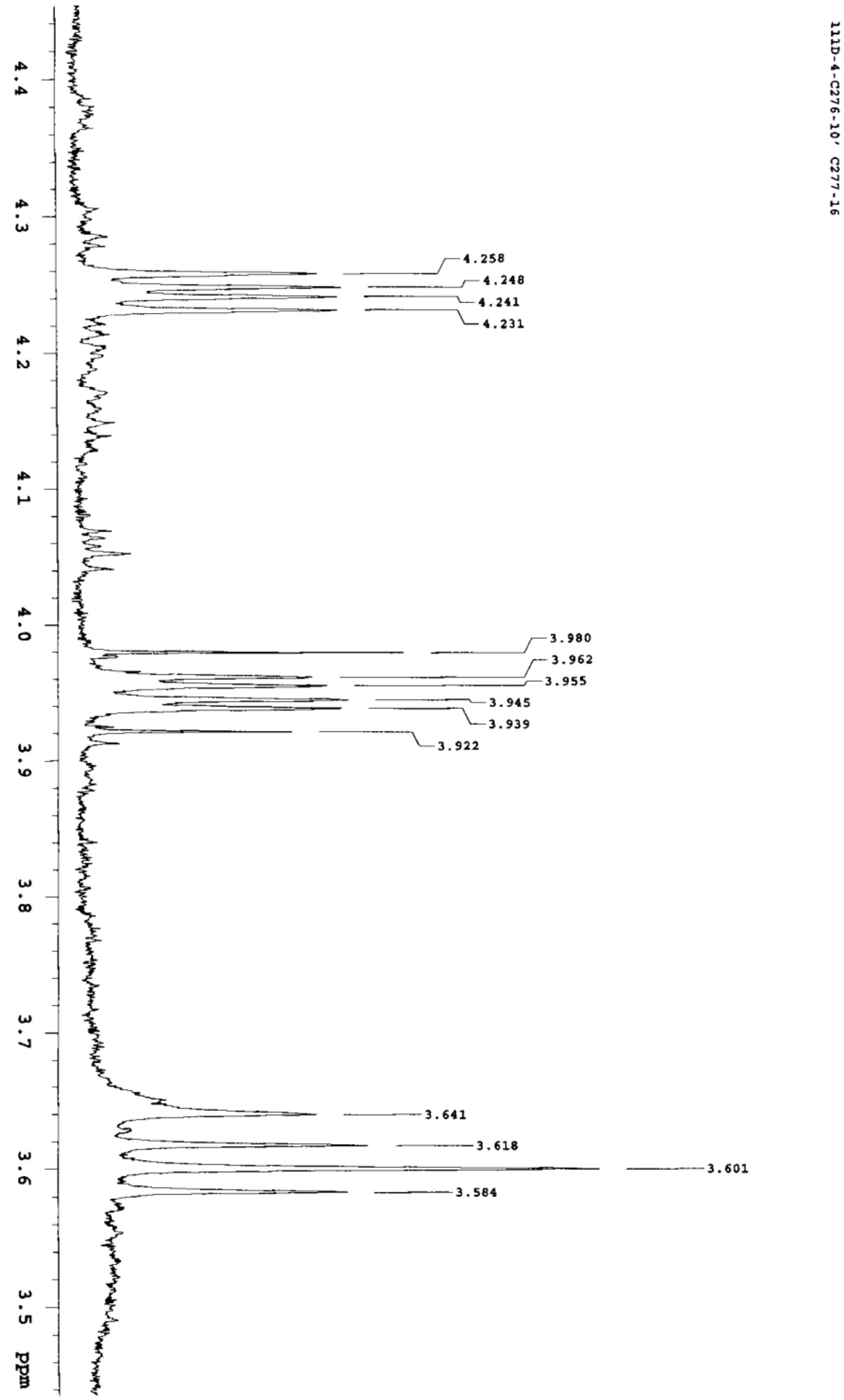
111D-4-C276-10' C277-16
4.258
4.248
4.241
4.231
3.980
3.962
3.955
3.945
3.939
3.922
3.641
3.618
3.601
3.584
4.4
4.3
4.2
4.1
4.0
3.9
3.8
3.7
3.6
3.5
ppm

**Figure S23** $^{13}$C NMR spectrum of 4 in $CDCl_3$

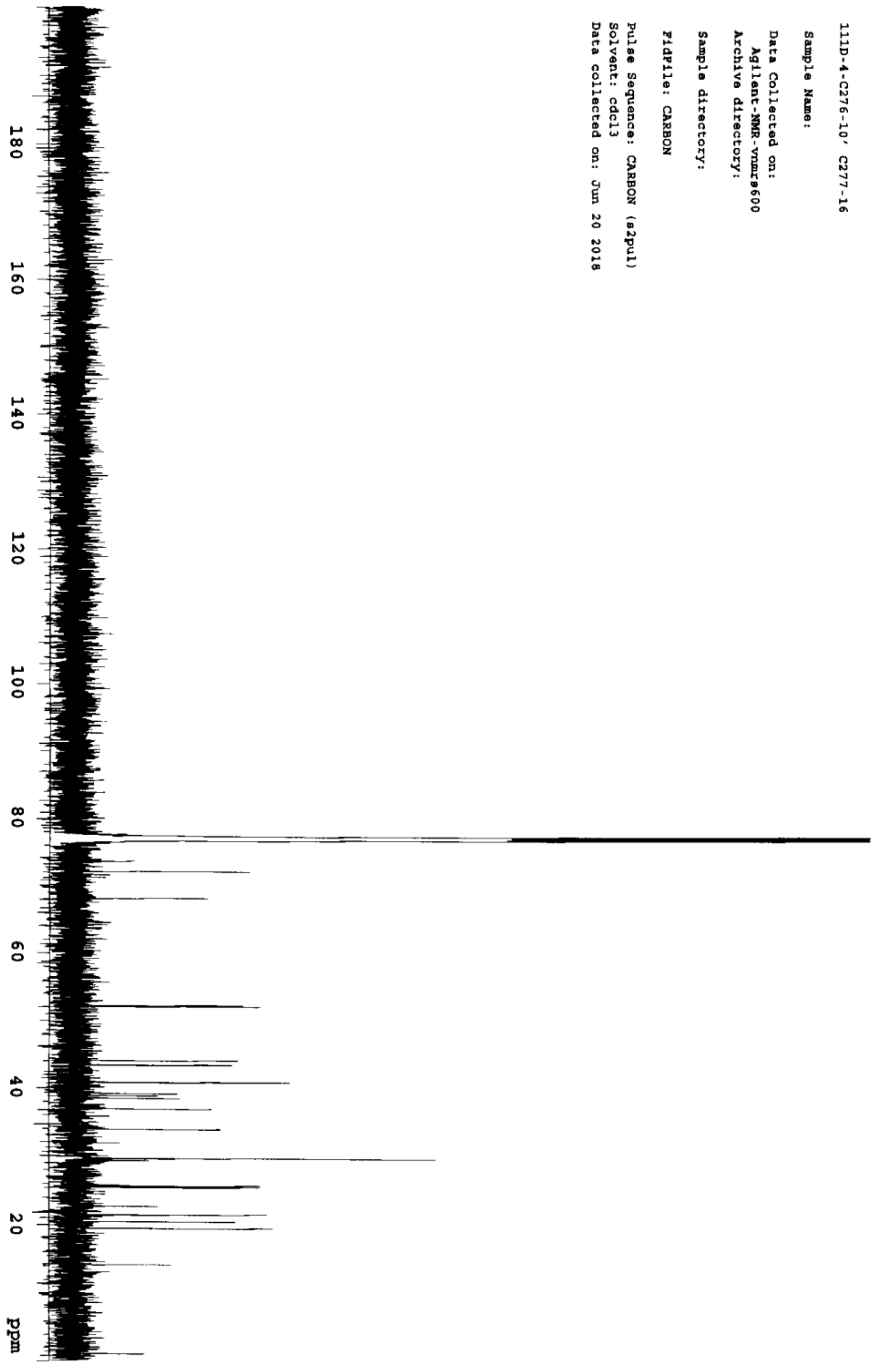

111D-4-C276-10' C277-16

| INDEX | FREQUENCY | PPM | HEIGHT |
|---|---|---|---|
| 1 | 28196.2 | 186.923 | -8.6 |
| 2 | 11647.3 | 77.215 | 6137.4 |
| 3 | 11615.0 | 77.000 | 6539.4 |
| 4 | 11582.6 | 76.785 | 5889.2 |
| 5 | 11105.2 | 73.620 | 11.5 |
| 6 | 10872.8 | 72.080 | 33.8 |
| 7 | 10266.0 | 68.057 | 25.7 |
| 8 | 7877.7 | 52.224 | 32.6 |
| 9 | 7851.1 | 52.048 | 36.1 |
| 10 | 6658.2 | 44.140 | 31.6 |
| 11 | 6554.1 | 43.450 | 30.5 |
| 12 | 6176.1 | 40.944 | 41.7 |
| 13 | 6164.6 | 40.867 | 37.6 |
| 14 | 5913.7 | 39.204 | 19.9 |
| 15 | 5872.1 | 38.928 | 16.0 |
| 16 | 5814.3 | 38.545 | 20.4 |
| 17 | 5572.7 | 36.944 | 26.6 |
| 18 | 5217.8 | 34.591 | -8.2 |
| 19 | 5124.2 | 33.970 | 28.5 |
| 20 | 4815.6 | 31.924 | 8.7 |
| 21 | 4479.2 | 29.694 | 70.3 |
| 22 | 4428.3 | 29.357 | 14.2 |
| 23 | 3905.8 | 25.893 | 8.5 |
| 24 | 3879.2 | 25.717 | 36.0 |
| 25 | 3853.8 | 25.548 | 32.5 |
| 26 | 3839.9 | 25.456 | 36.0 |
| 27 | 3421.5 | 22.682 | 16.0 |
| 28 | 3282.8 | 21.763 | -8.4 |
| 29 | 3240.0 | 21.479 | 37.4 |
| 30 | 3088.6 | 20.475 | 31.2 |
| 31 | 2940.6 | 19.494 | 38.6 |
| 32 | 2127.9 | 14.107 | 18.7 |
| 33 | 152.4 | 1.010 | 13.3 |

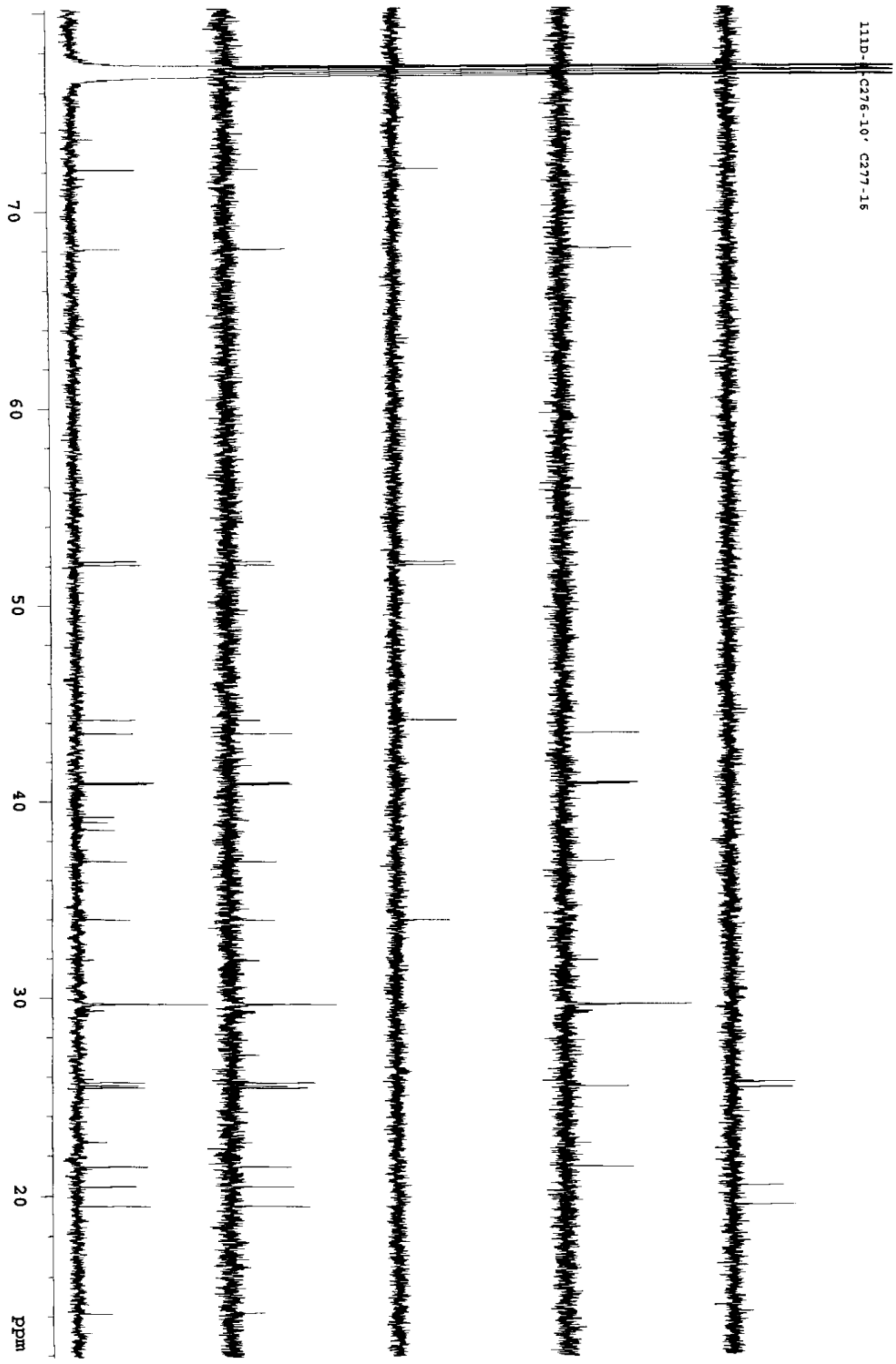

C276-10' C277-16
70
60
50
40
30
20
ppm

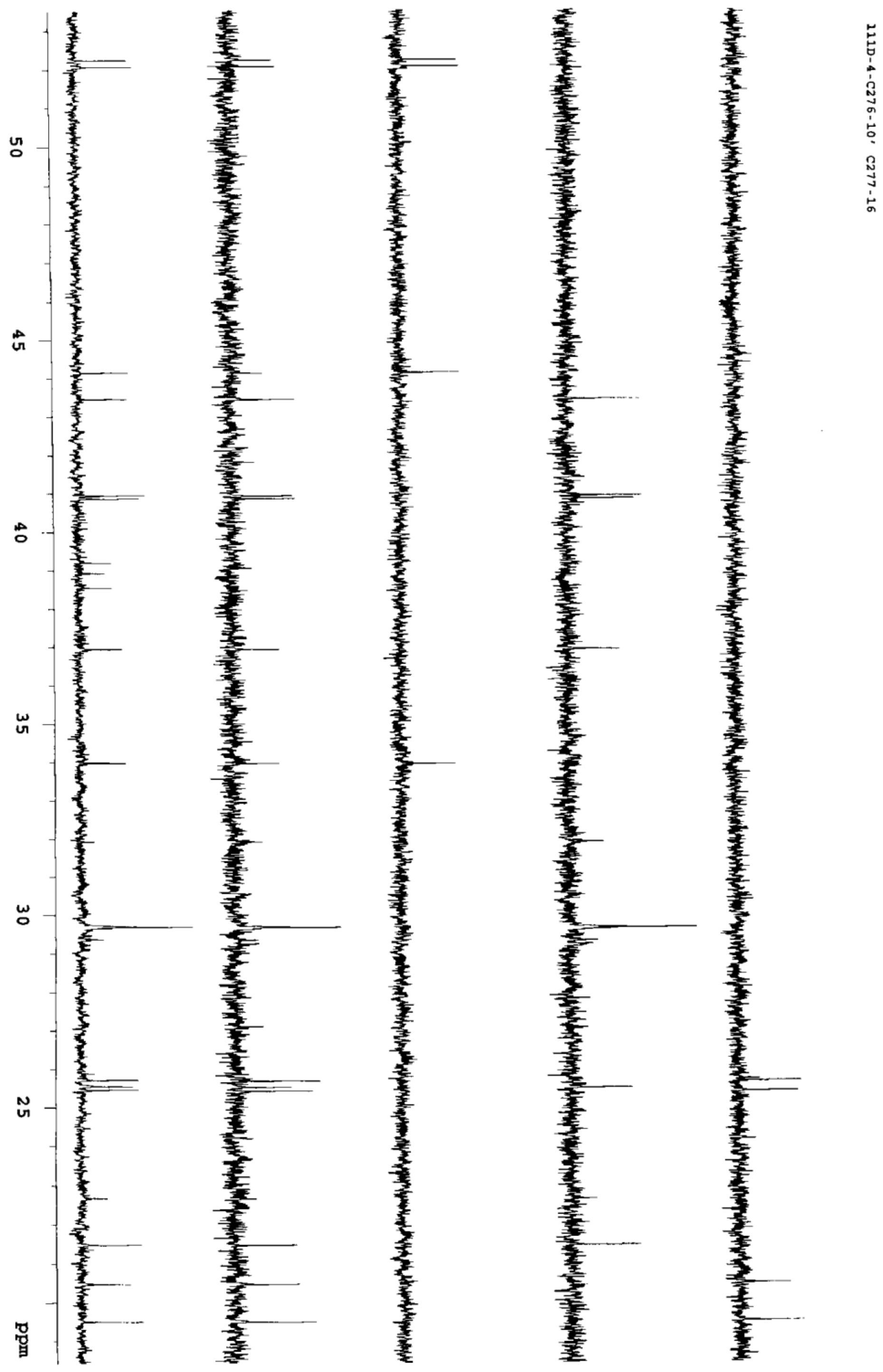
111D-4-C276-10' C277-16
50
45
40
35
30
25
ppm

**Figure S24 $^{1}$H-$^{1}$H COSY of 4**

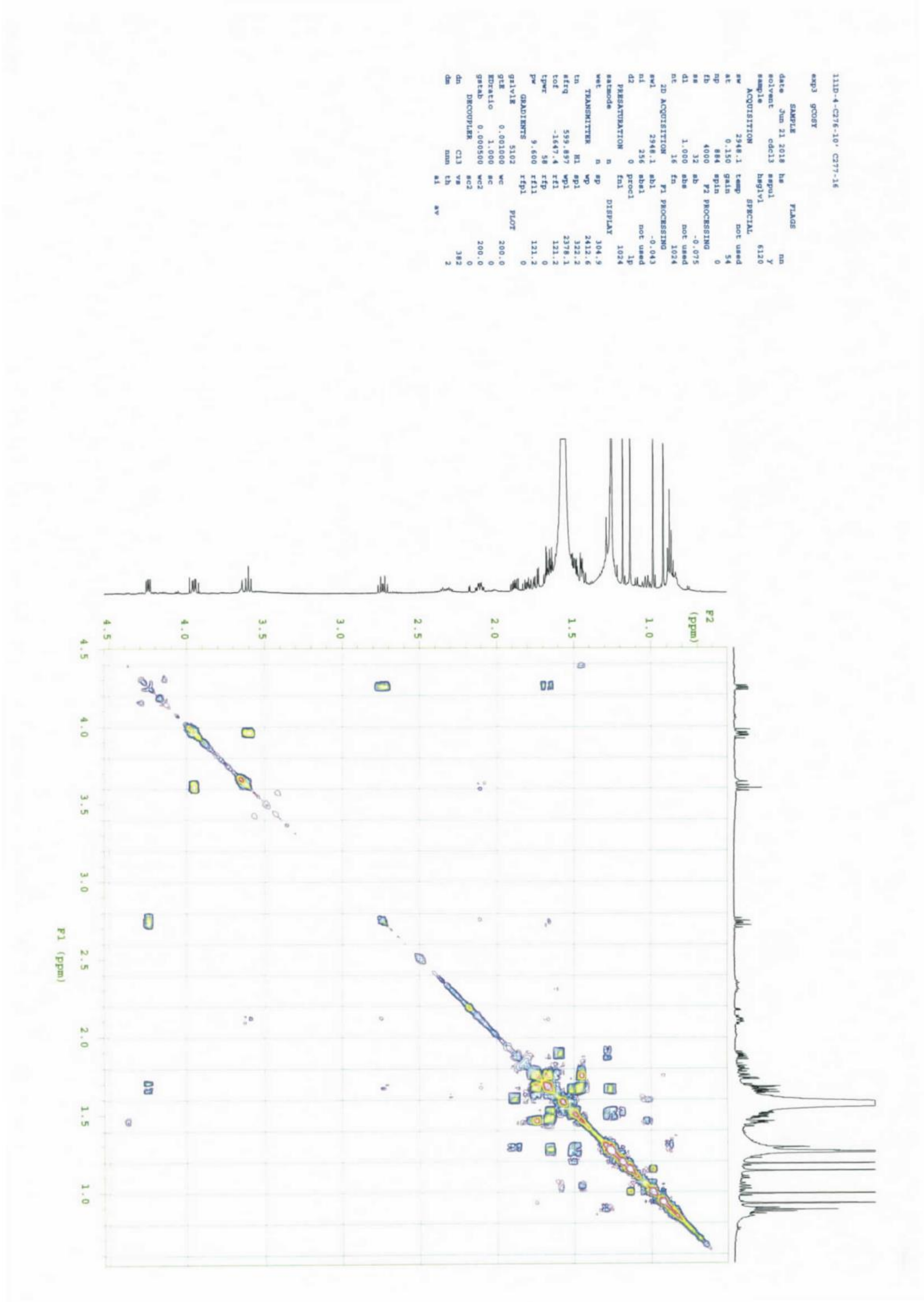

111D-4-C276-10' C277-16

exp3 gCOSY

| | | | |
|---|---|---|---|
| SAMPLE | | FLAGS | |
| date | Jun 21 2018 | hs | nn |
| solvent | cdcl3 | sspul | y |
| sample | | hsglvl | 6120 |
| ACQUISITION | | SPECIAL | |
| sw | 2948.1 | temp | not used |
| at | 0.150 | gain | 54 |
| np | 884 | spin | 0 |
| fb | 4000 | F2 PROCESSING | |
| ss | 32 | sb | -0.075 |
| d1 | 1.000 | sbs | not used |
| nt | 16 | fn | 1024 |
| 2D ACQUISITION | | F1 PROCESSING | |
| sw1 | 2948.1 | sb1 | -0.043 |
| ni | 256 | sbs1 | not used |
| d2 | 0 | proc1 | lp |
| PRESATURATION | | fn1 | 1024 |
| satmode | n | DISPLAY | |
| wet | n | sp | 420.1 |
| TRANSMITTER | | wp | 1307.1 |
| tn | H1 | sp1 | 414.3 |
| sfrq | 599.897 | wp1 | 1312.8 |
| tof | -1647.4 | rfl | 121.2 |
| tpwr | 58 | rfp | 0 |
| pw | 9.600 | rfl1 | 121.2 |
| GRADIENTS | | rfp1 | 0 |
| gzlvlE | 5102 | PLOT | |
| gtE | 0.001000 | wc | 200.0 |
| EDratio | 1.000 | sc | 0 |
| gstab | 0.000500 | wc2 | 200.0 |
| DECOUPLER | | sc2 | 0 |
| dn | C13 | vs | 382 |
| dm | nnn | th | 2 |
| | | ai | av |

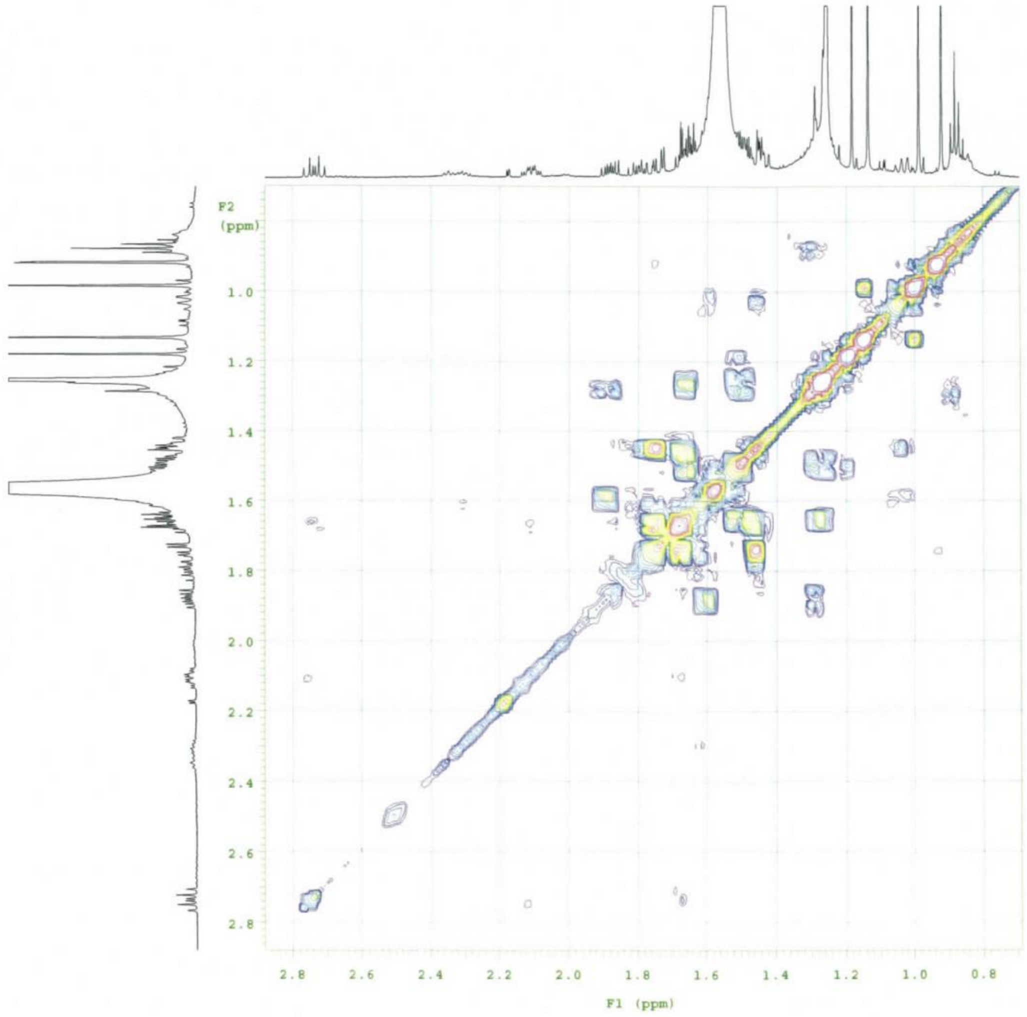

**Figure S25 NOESY of 4**

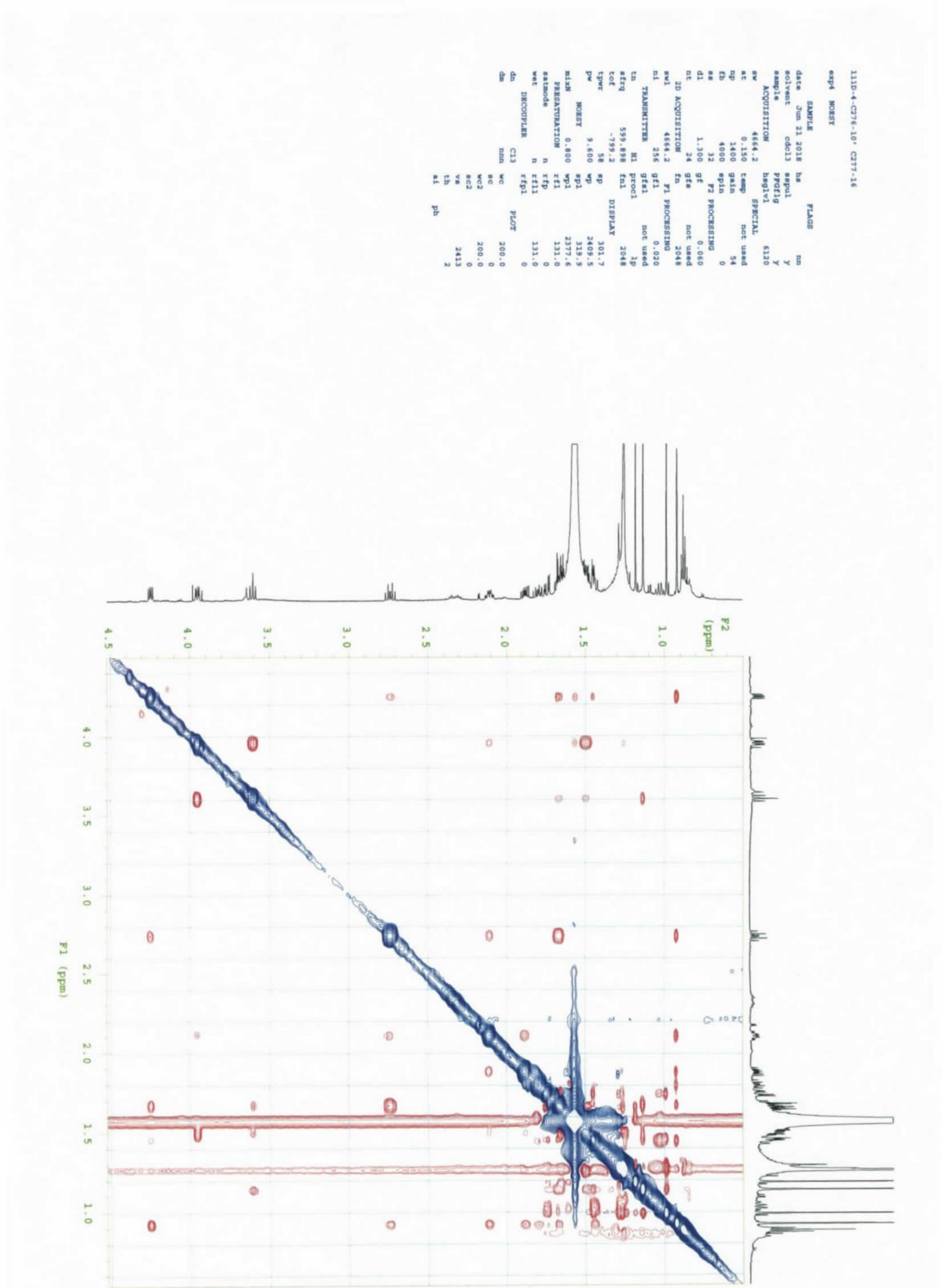

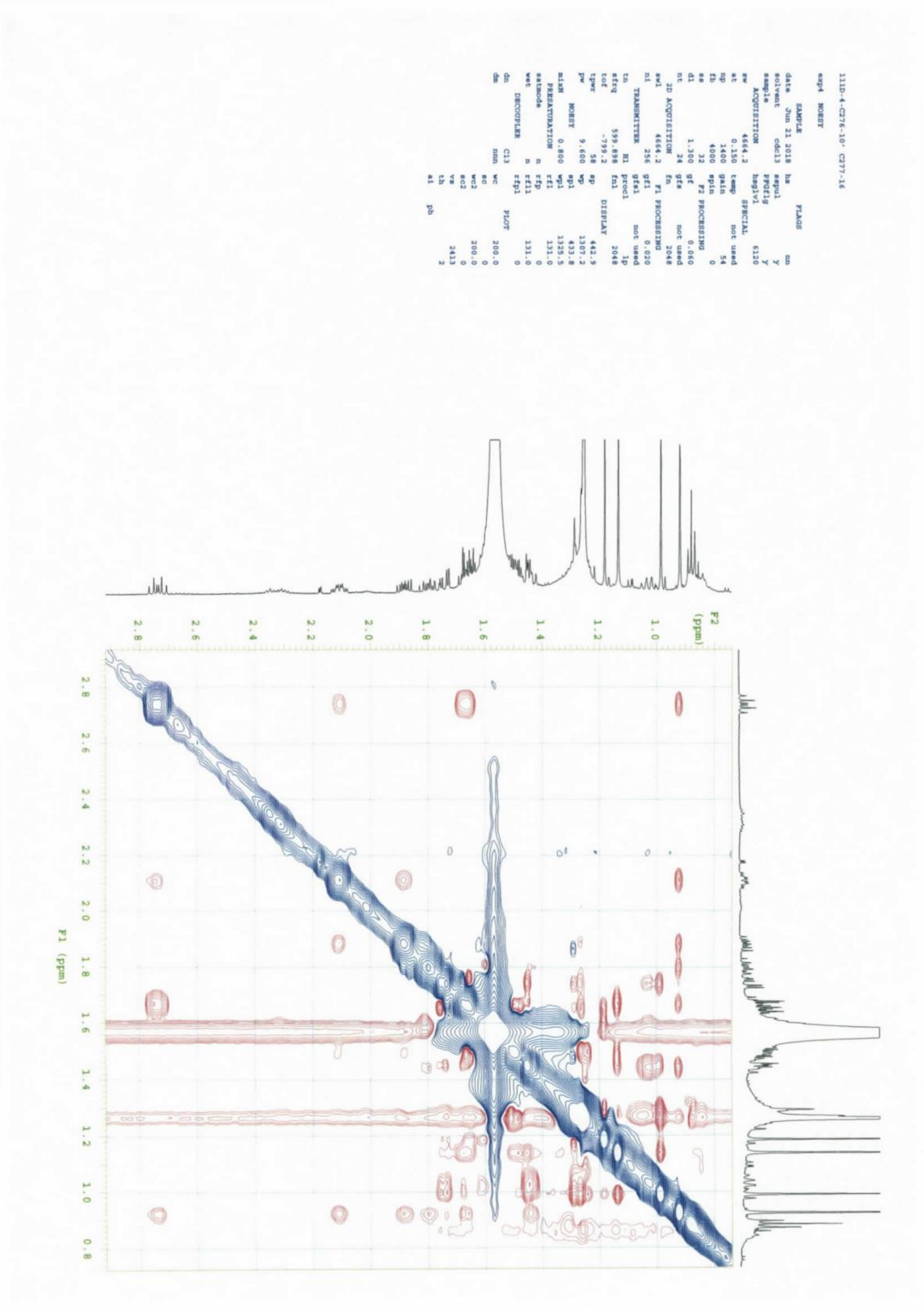
F2 (ppm)
F1 (ppm)

**Figure S26    HMQC of 4**

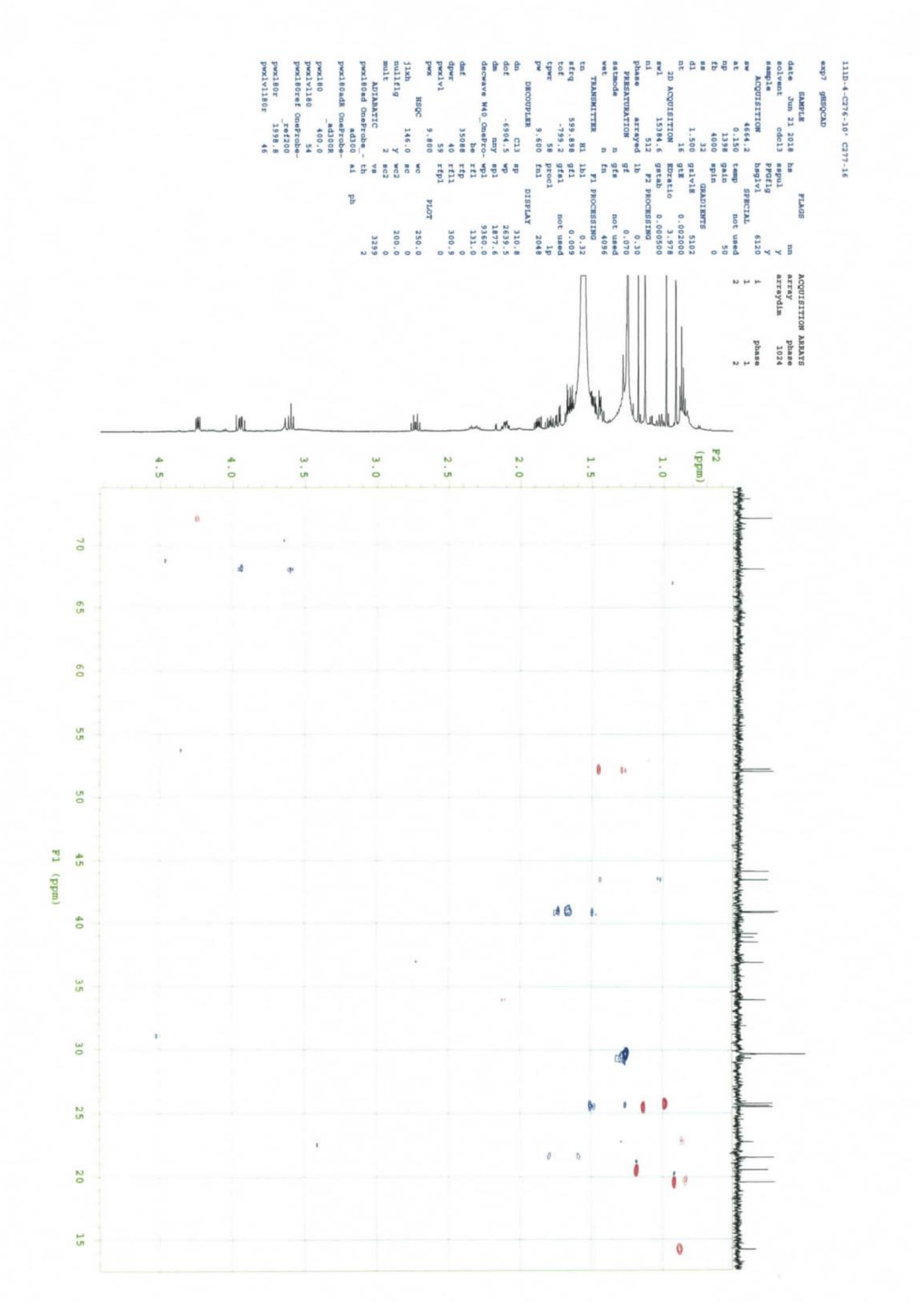

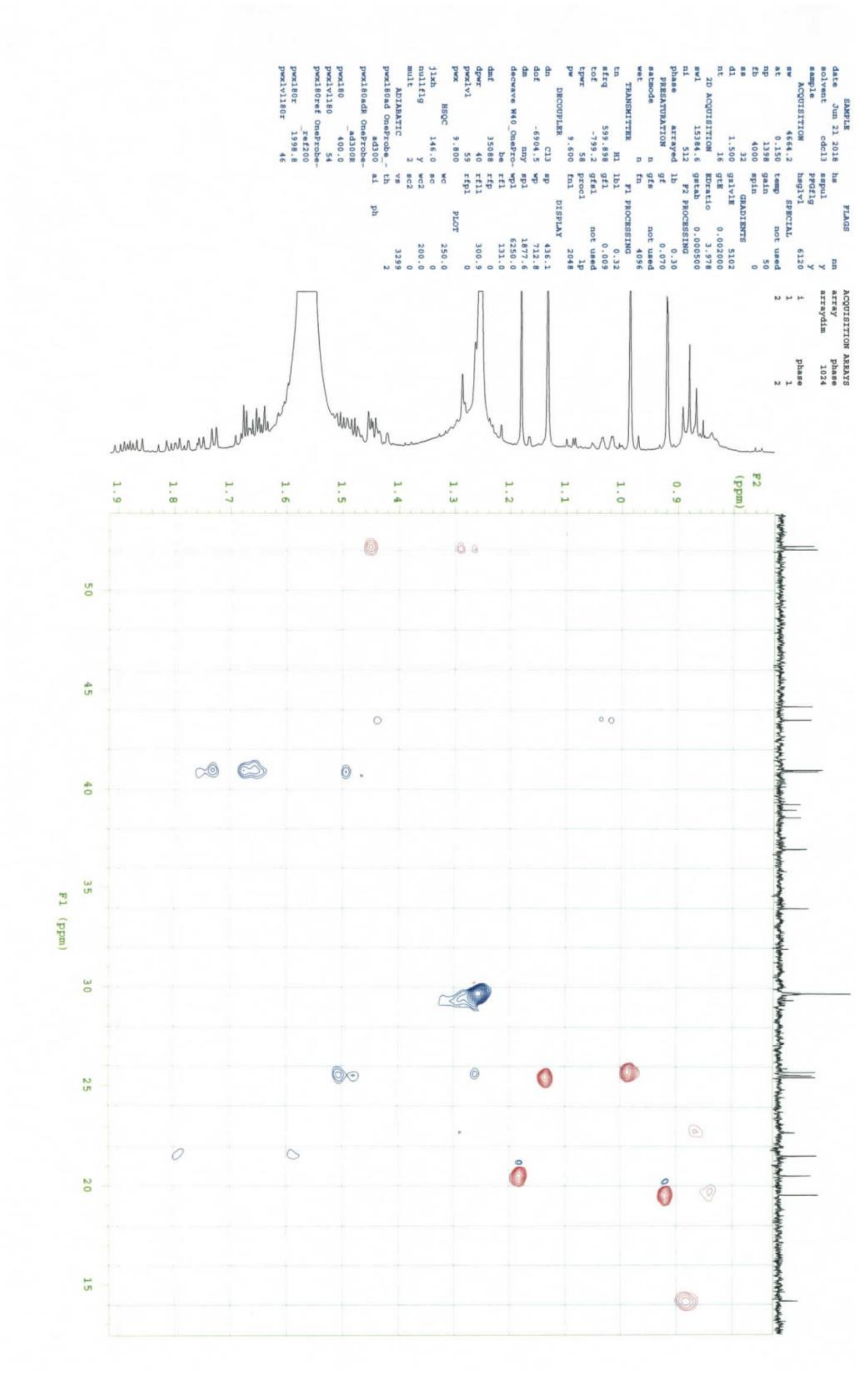
F2 (ppm)
F1 (ppm)

**Figure S27 HMBC of 4**

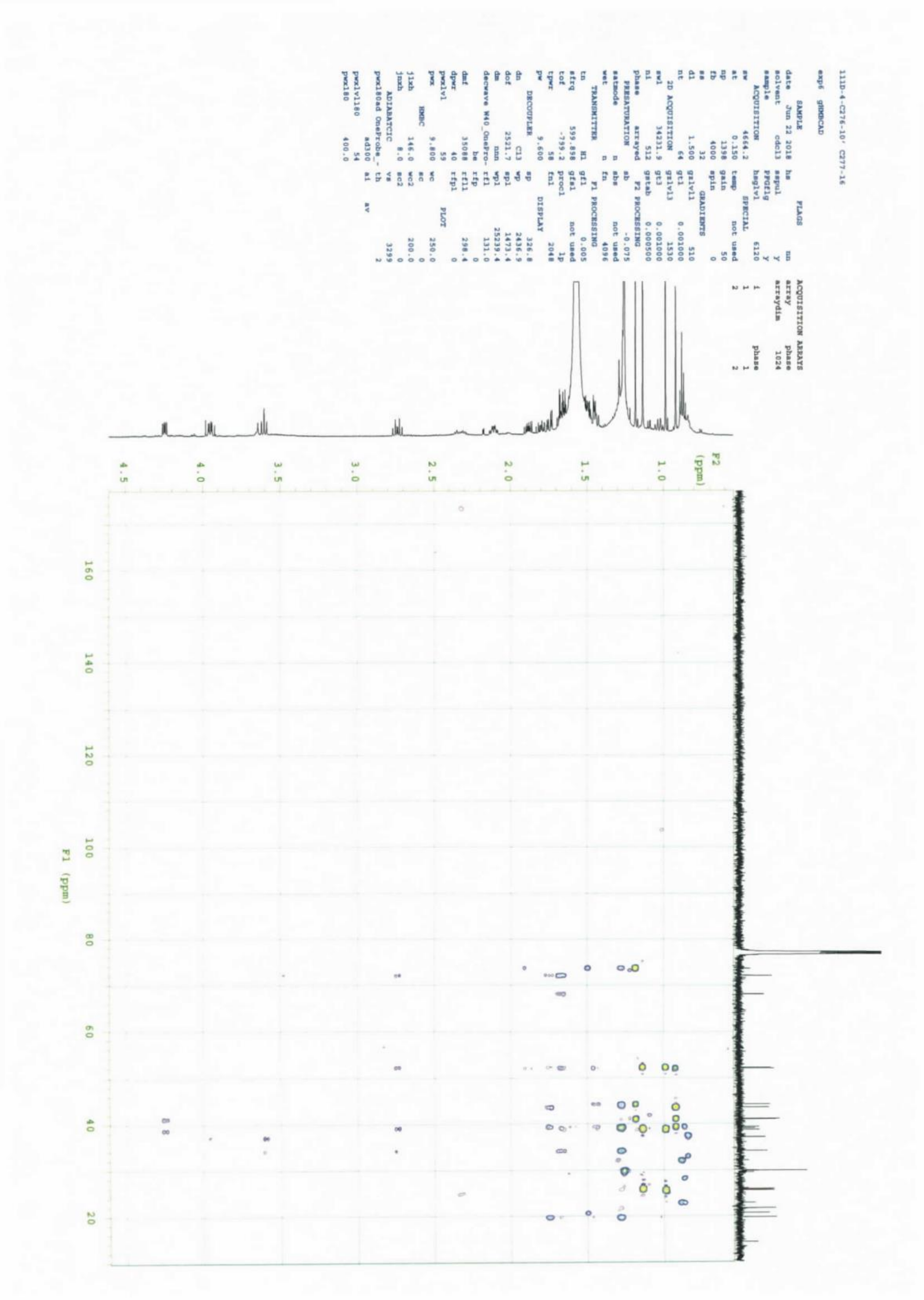

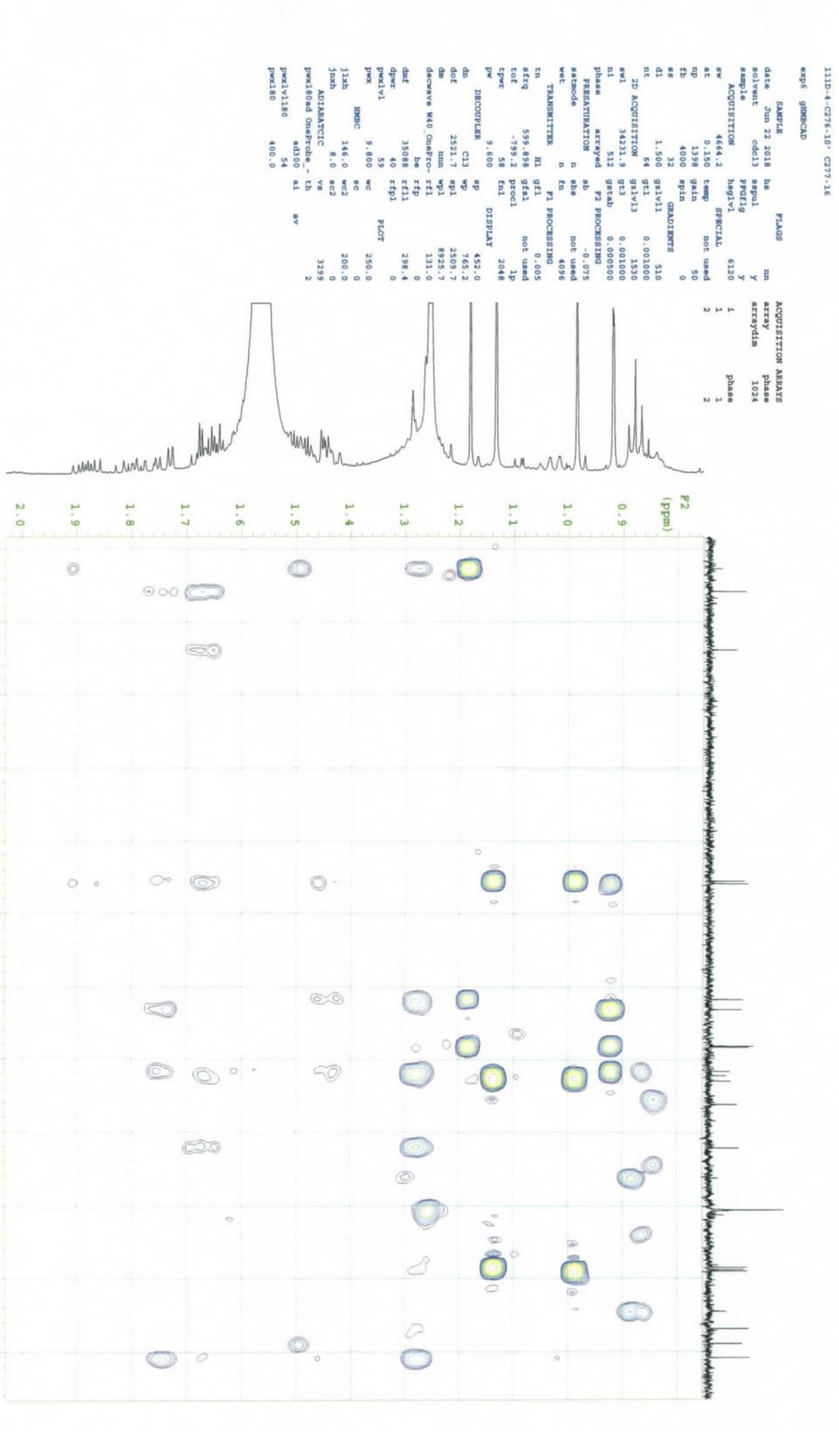
111D-4-C276-10' C277-16
exp6 gHMBCAD
F2 (ppm)
F1 (ppm)

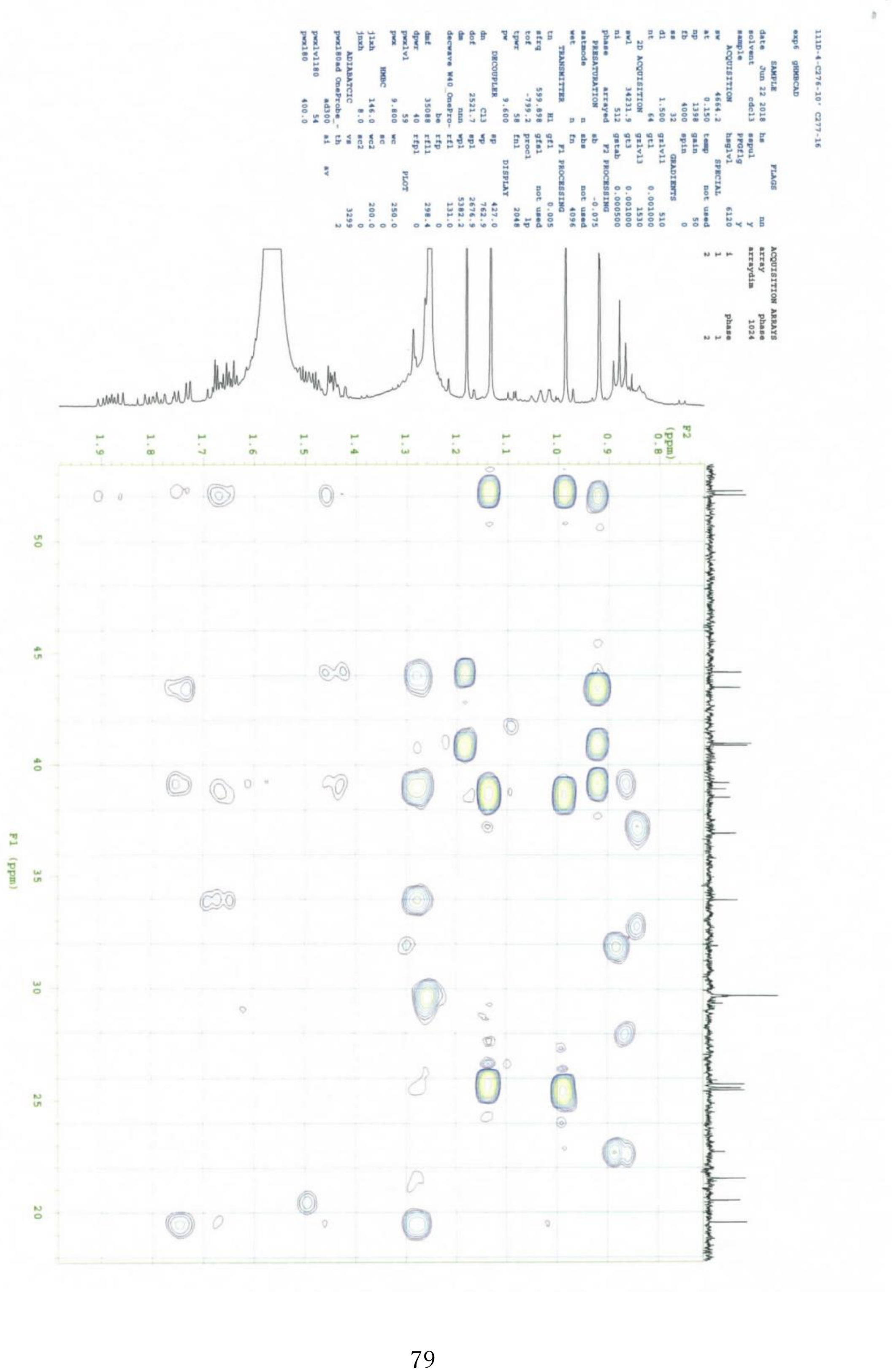

**Figure S28 FABMS of 4**

```
[ Elemental Composition ]                                    Page: 1
Data  : 1808049                     Date : 07-Aug-2018 20:44
Sample: 111D-4 C277-16
Note : Matrix; G
Inlet : Direct                      Ion Mode : FAB+
RT : 4.67 min                       Scan#: (30,35)
Elements : C 25/15, H 40/30, O 10/0, Na 1/0
Mass Tolerance      : 20ppm, 1mmu if m/z > 50
Unsaturation (U.S.) : -1.0 - 40.0

Observed m/z Int%   Err[ppm / mmu]    U.S.  Composition
  345.2415   100.0     +2.7 /  +0.9    3.5  C 20 H 34 O 3 Na
```

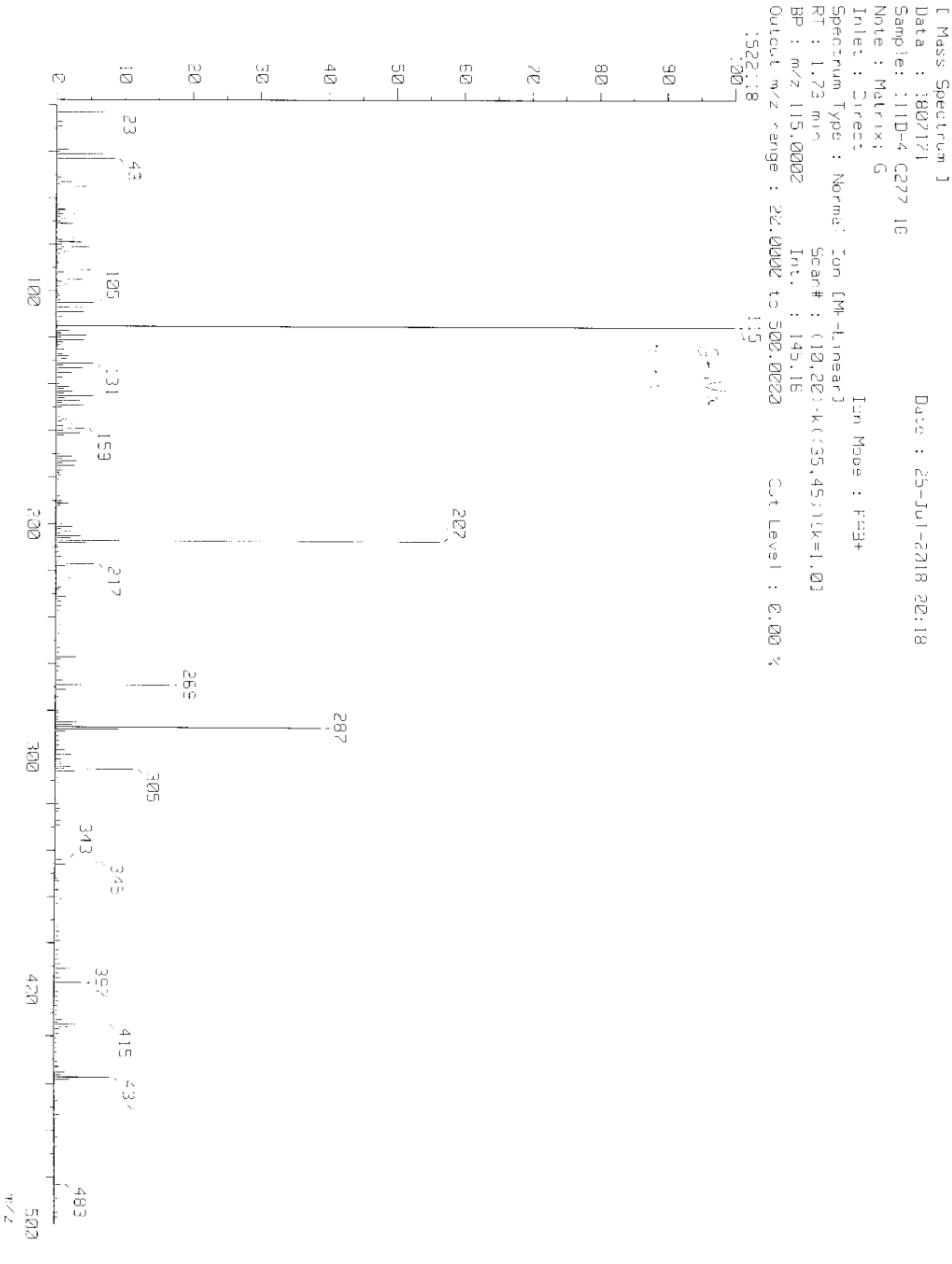
[ Mass Spectrum ]
Data : 1807171
Date : 25-Jul-2018 20:18
Note : Matrix: G
Inlet : Direct
Ion Mode : FAB+
Spectrum Type : Normal Ion [MF-Linear]
RT : 1.73 min
Scan# : (10,20)-k((35,45))(k=1.0)
BP : m/z 115.0002
Int. : 145.16
Output m/z range : 22.0000 to 500.0000
Cut Level : 0.00 %
23
43
105
131
159
207
217
269
287
305
343
345
397
415
437
483
m/z

**Figure S29 $^{1}$H NMR spectrum of 2a in $CDCl_3$**

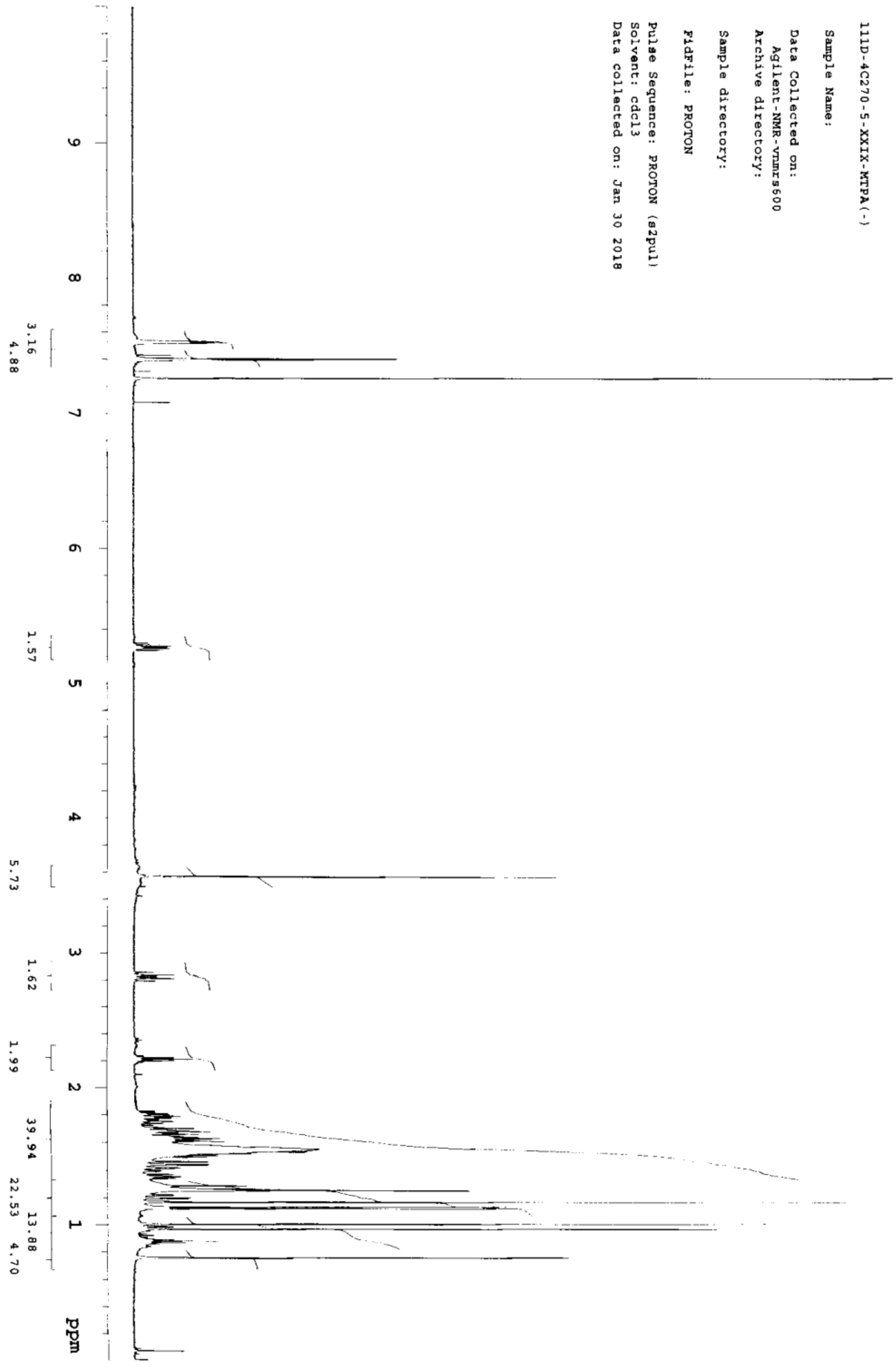

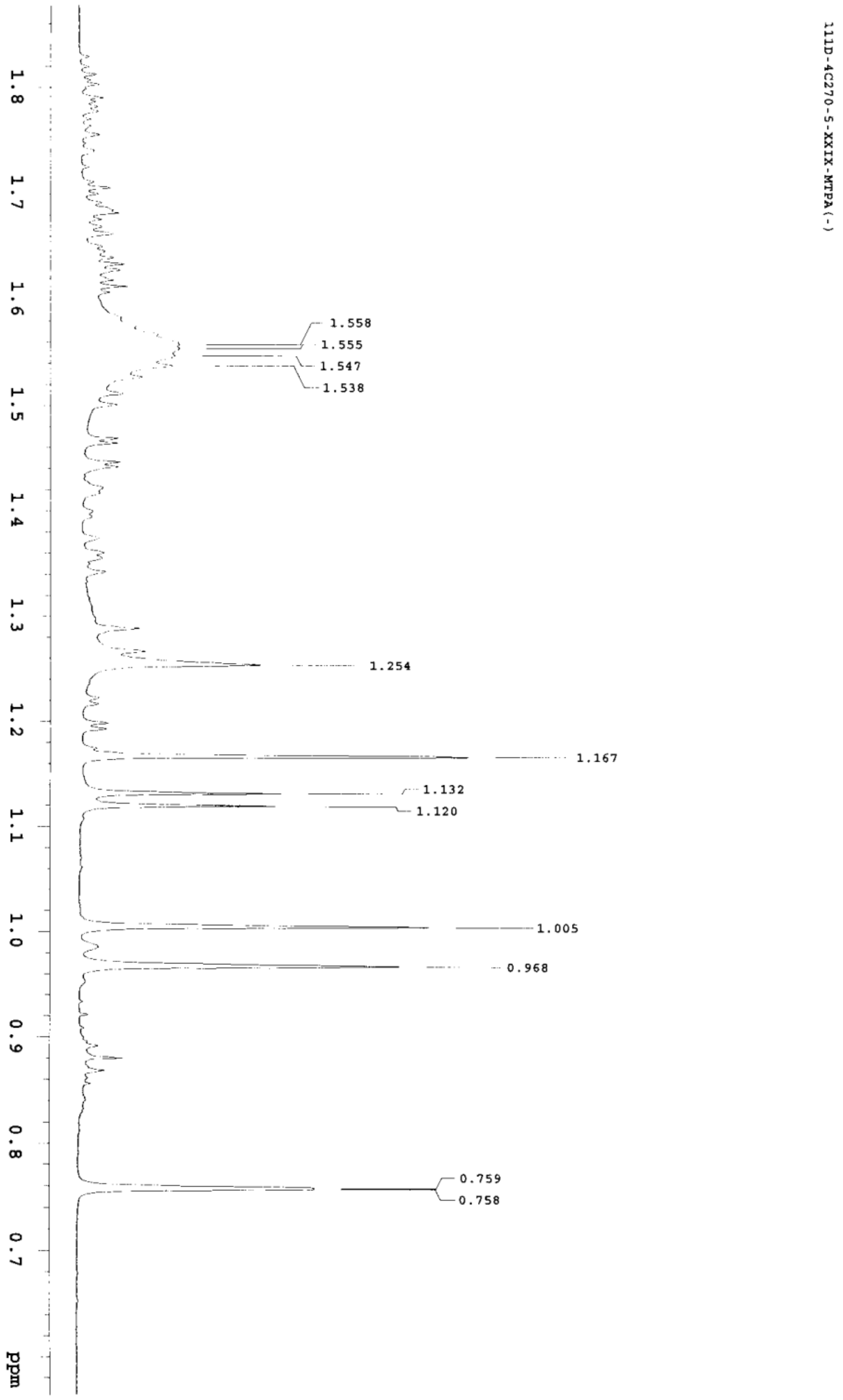
111D-4C270-5-XXIX-MTPA(-)
1.558
1.555
1.547
1.538
1.254
1.167
1.132
1.120
1.005
0.968
0.759
0.758
1.8
1.7
1.6
1.5
1.4
1.3
1.2
1.1
1.0
0.9
0.8
0.7
ppm

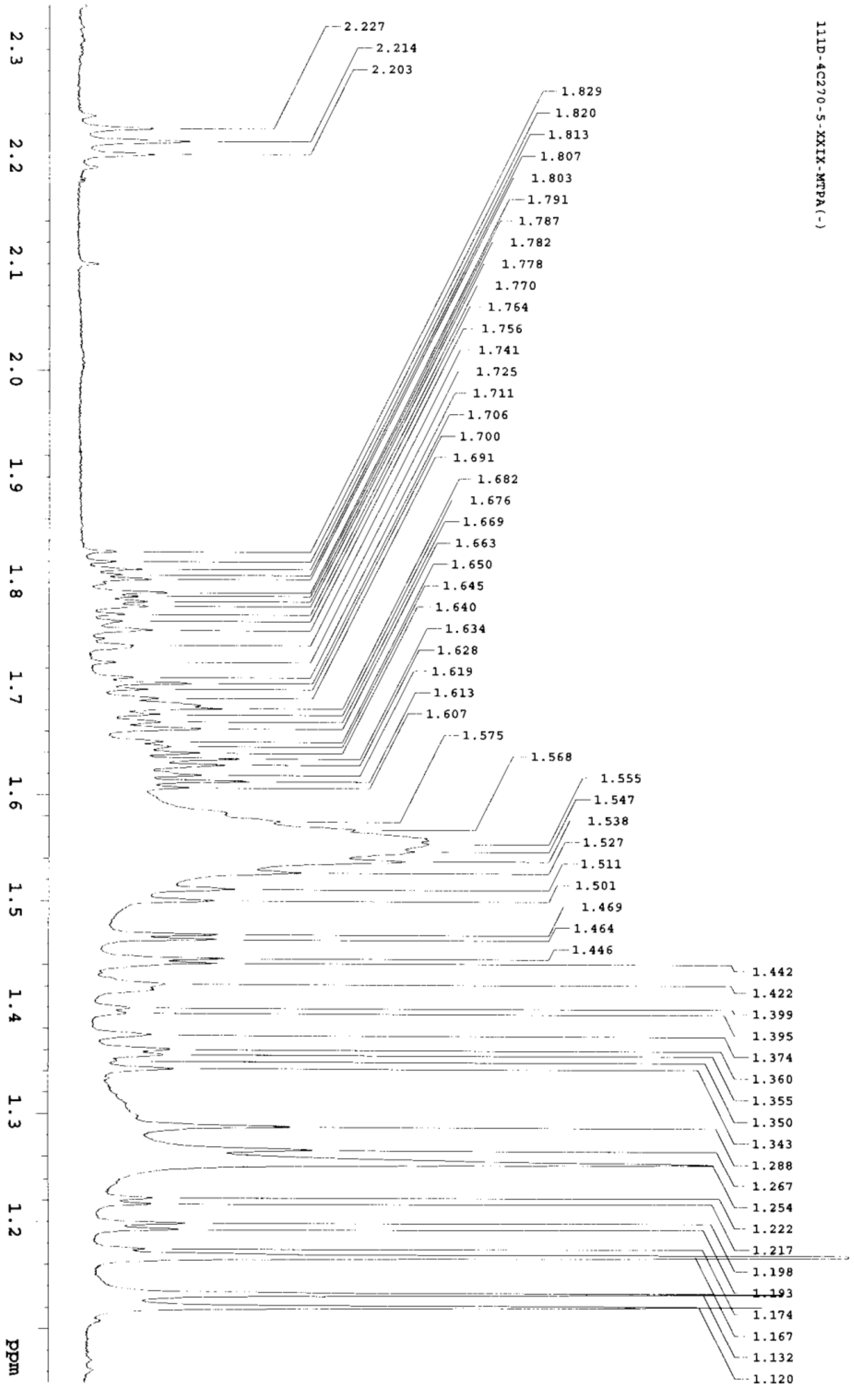
111D-4C270-5-XXIX-MTPA(-)
2.227
2.214
2.203
1.829
1.820
1.813
1.807
1.803
1.791
1.787
1.782
1.778
1.770
1.764
1.756
1.741
1.725
1.711
1.706
1.700
1.691
1.682
1.676
1.669
1.663
1.650
1.645
1.640
1.634
1.628
1.619
1.613
1.607
1.575
1.568
1.555
1.547
1.538
1.527
1.511
1.501
1.469
1.464
1.446
1.442
1.422
1.399
1.395
1.374
1.360
1.355
1.350
1.343
1.288
1.267
1.254
1.222
1.217
1.198
1.193
1.174
1.167
1.132
1.120
2.3
2.2
2.1
2.0
1.9
1.8
1.7
1.6
1.5
1.4
1.3
1.2
ppm

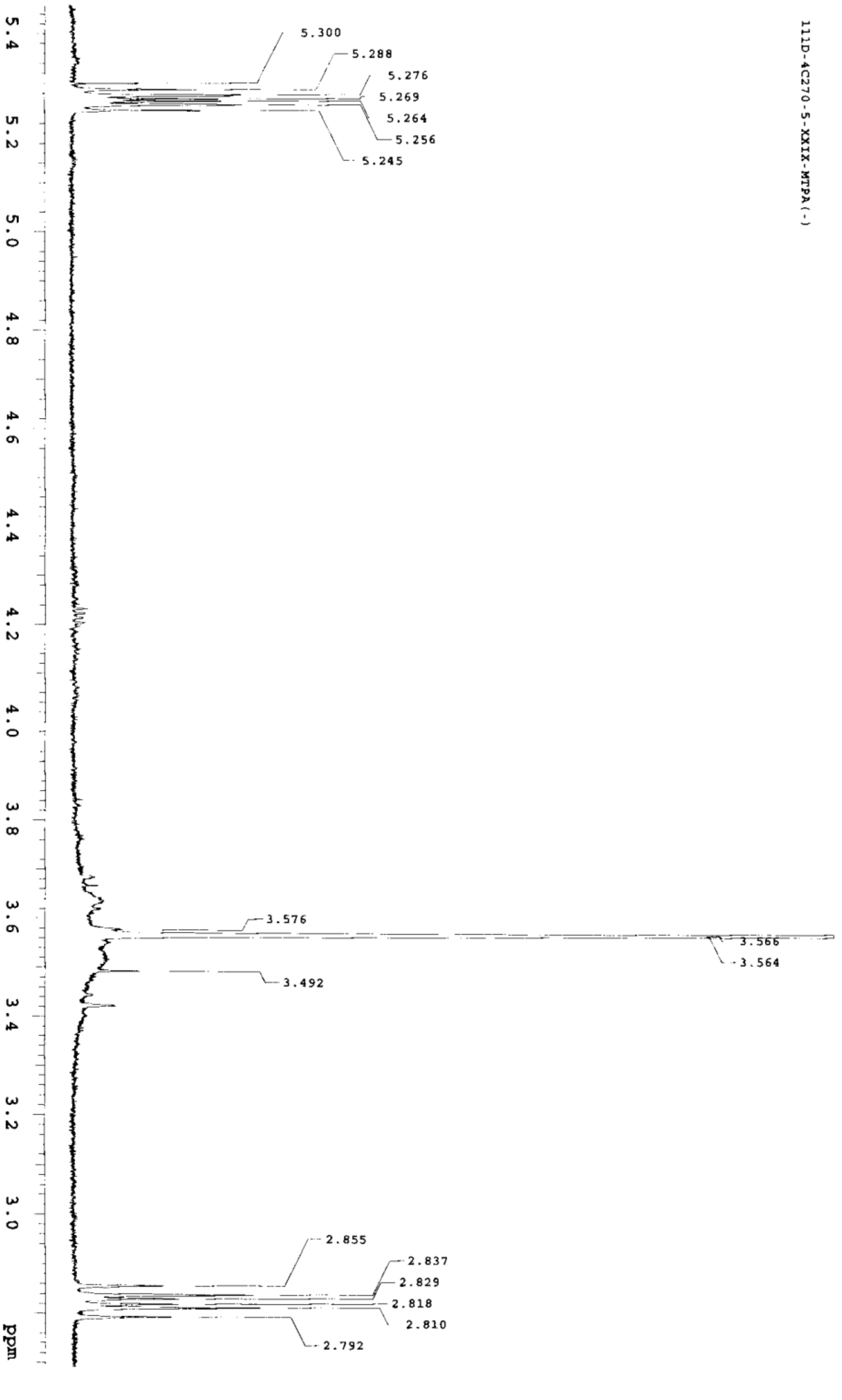
111D-4C270-5-XXIX-MTPA(-)
5.300
5.288
5.276
5.269
5.264
5.256
5.245
3.576
3.566
3.564
3.492
2.855
2.837
2.829
2.818
2.810
2.792
5.4
5.2
5.0
4.8
4.6
4.4
4.2
4.0
3.8
3.6
3.4
3.2
3.0
ppm

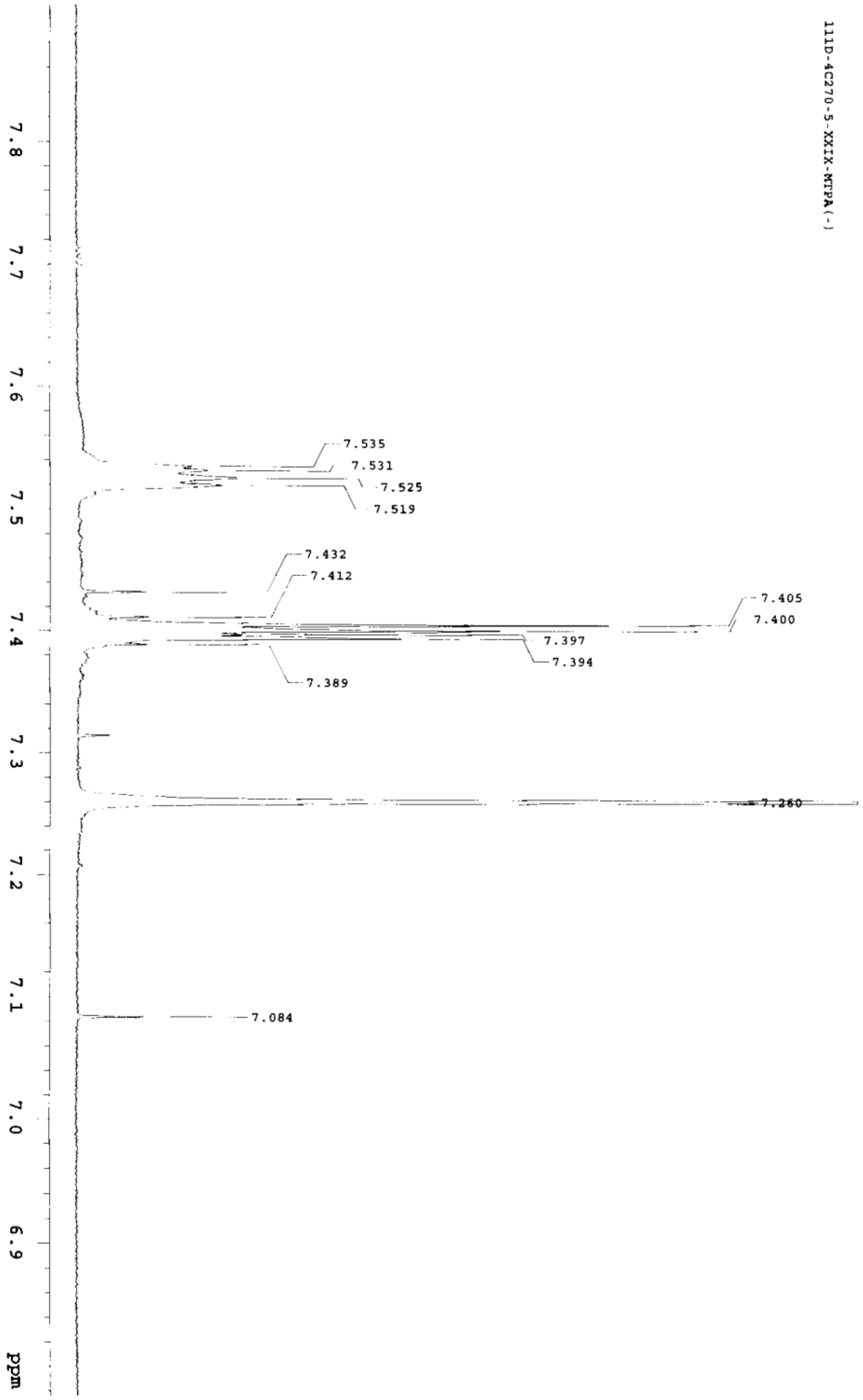
111D-4C270-5-XXIX-MTPA(-)
7.535
7.531
7.525
7.519
7.432
7.412
7.405
7.400
7.397
7.394
7.389
7.260
7.084
7.8
7.7
7.6
7.5
7.4
7.3
7.2
7.1
7.0
6.9
ppm

**Figure S30** $^{1}H$-$^{1}H$ **COSY of 2a**

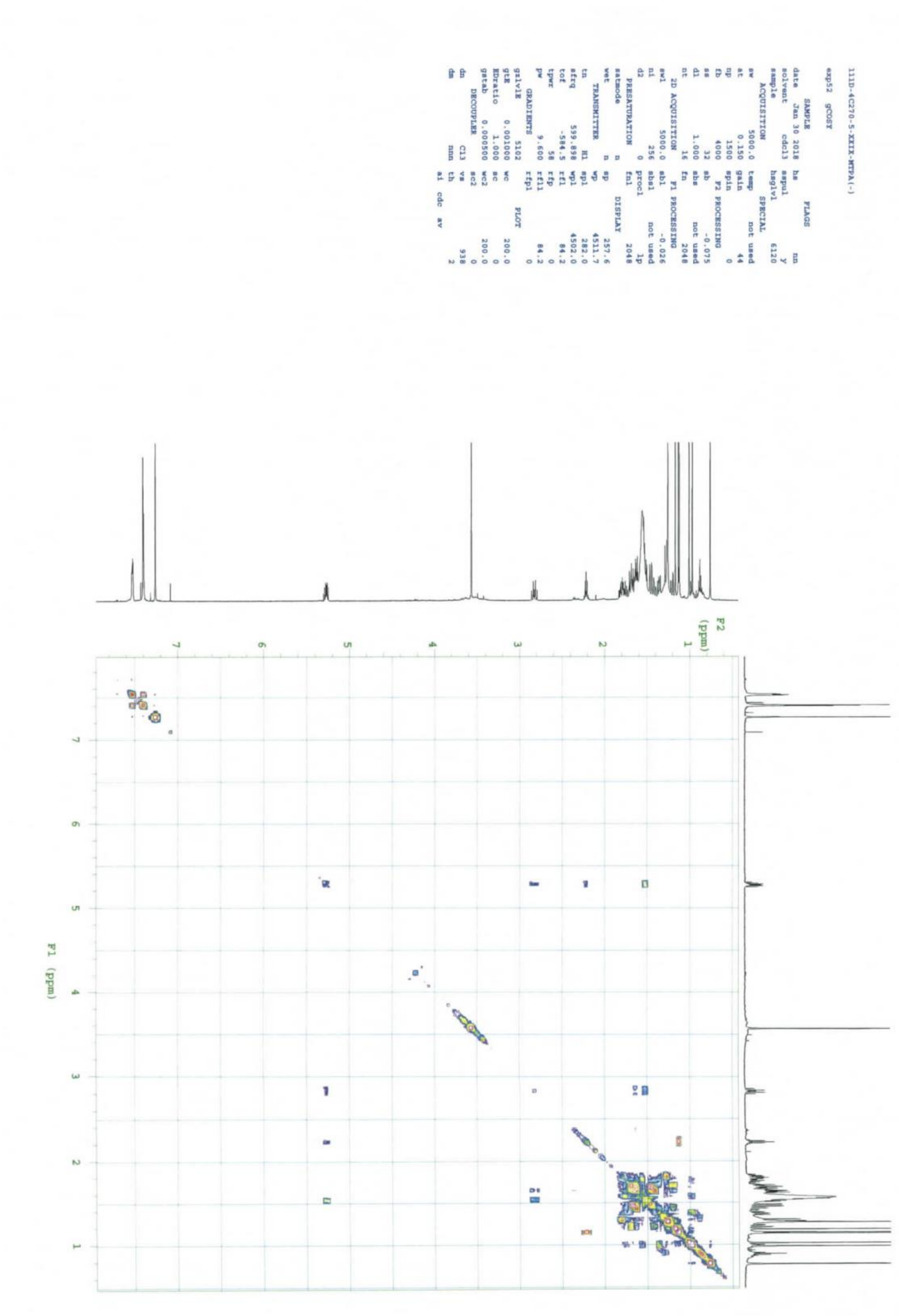

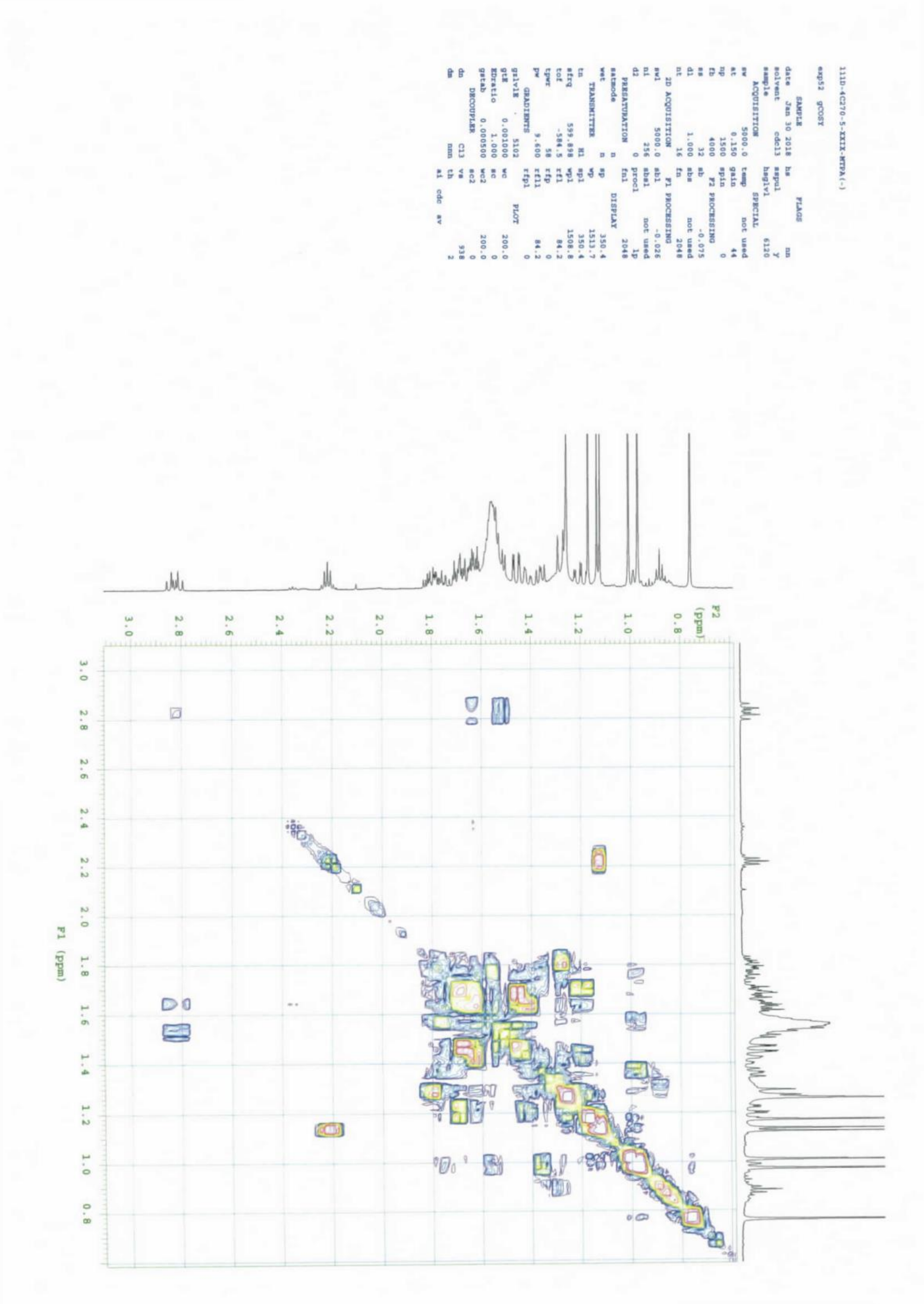
111D-4C270-5-XXIX-MTPA(-)
exp52 gCOSY
SAMPLE
date Jan 30 2018
solvent cdcl3
sample
ACQUISITION
sw 5000.0
at 0.150
np 1500
fb 4000
ss 32
d1 1.000
nt 16
2D ACQUISITION
sw1 5000.0
ni 256
d2 0
PRESATURATION
satmode n
wet n
TRANSMITTER
tn H1
sfrq 599.898
tof -584.5
tpwr 58
pw 9.600
GRADIENTS
gzlvlE 5102
gtE 0.001000
EDratio 1.000
gstab 0.000500
DECOUPLER
dn C13
dm nnn
FLAGS
hs nn
sspul y
hsglvl 6120
SPECIAL
temp not used
gain 44
spin 0
F2 PROCESSING
sb -0.075
sbs not used
fn 2048
F1 PROCESSING
sb1 -0.026
sbs1 not used
proc1 lp
fn1 2048
DISPLAY
sp 350.4
wp 1513.7
sp1 350.4
wp1 1508.8
rfl 84.2
rfp 0
rfl1 84.2
rfp1 0
PLOT
wc 200.0
sc 0
wc2 200.0
sc2 0
vs 938
th 2
ai cdc av
F2 (ppm)
F1 (ppm)

**Figure S31 NOESY of 2a**

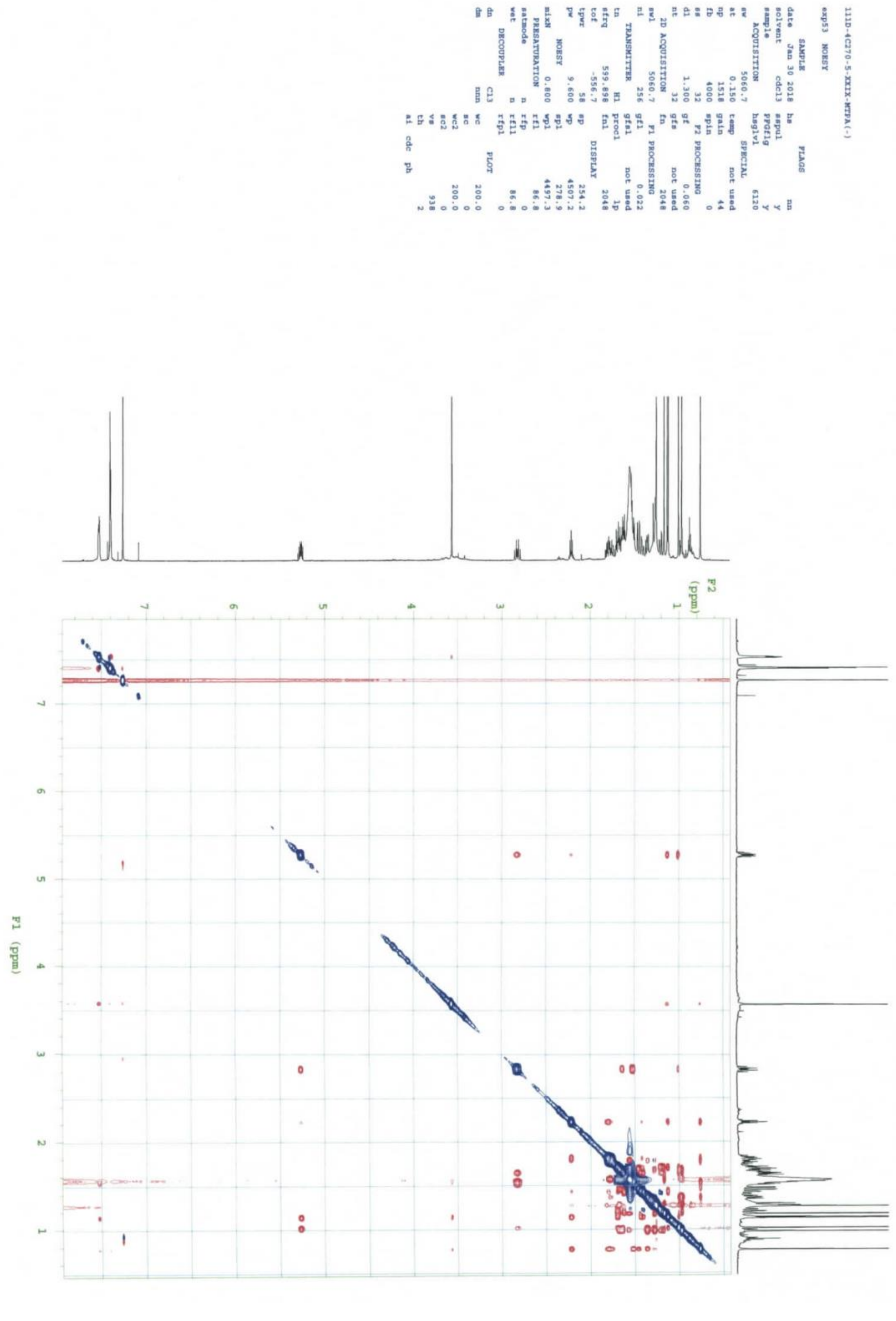

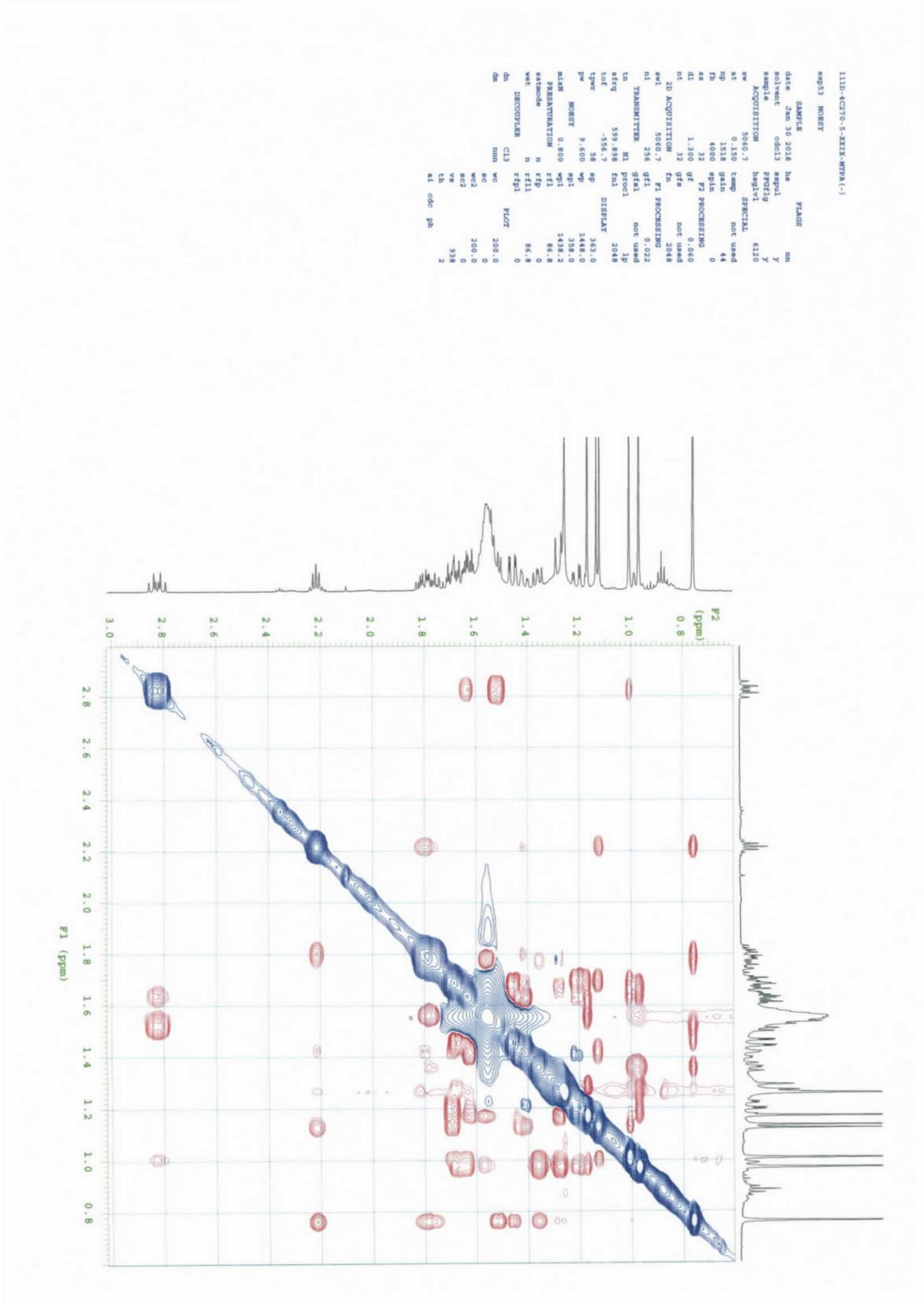
F2 (ppm)
F1 (ppm)

**Figure S32  FABMS of 2a**

```
[ Elemental Composition ]                                        Page: 1
Data  : 1802046                      Date : 14-Feb-2018 18:14
Sample: 111D-4 C271-5 MTPA(-) XXIX
Note : Matrix; NBA+Na
Inlet : Direct                       Ion Mode : FAB+
RT : 4.77 min                        Scan#: (40,45)
Elements : C 33/28, H 45/35, F 5/0, O 10/0, Na 1/0
Mass Tolerance       : 20ppm, 1mmu if m/z > 50
Unsaturation (U.S.) : -1.0 - 30.0

Observed m/z Int%   Err[ppm / mmu]    U.S.  Composition
  545.2860   100.0     +1.0 /  +0.5    8.5  C 30 H 41 F 3 O 4 Na
```

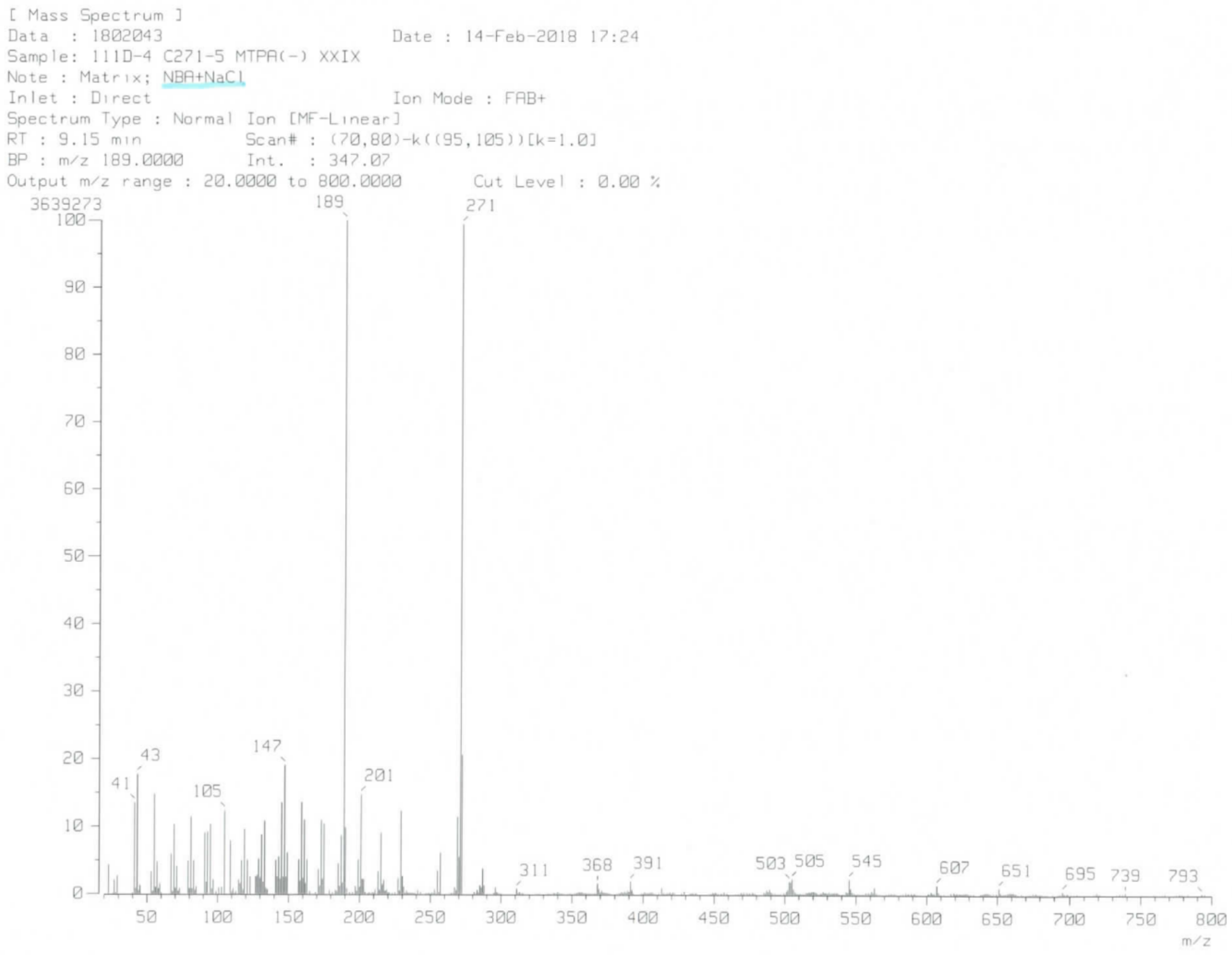
[ Mass Spectrum ]
Data : 1802043
Date : 14-Feb-2018 17:24
Sample: 111D-4 C271-5 MTPA(-) XXIX
Note : Matrix; NBA+NaCl
Inlet : Direct
Ion Mode : FAB+
Spectrum Type : Normal Ion [MF-Linear]
RT : 9.15 min
Scan# : (70,80)-k((95,105))[k=1.0]
BP : m/z 189.0000
Int. : 347.07
Output m/z range : 20.0000 to 800.0000
Cut Level : 0.00 %
3639273
189
271
41
43
105
147
201
311
368
391
503
505
545
607
651
695
739
793
m/z

**Figure S33** $^1$H NMR spectrum of 2b in $CDCl_3$

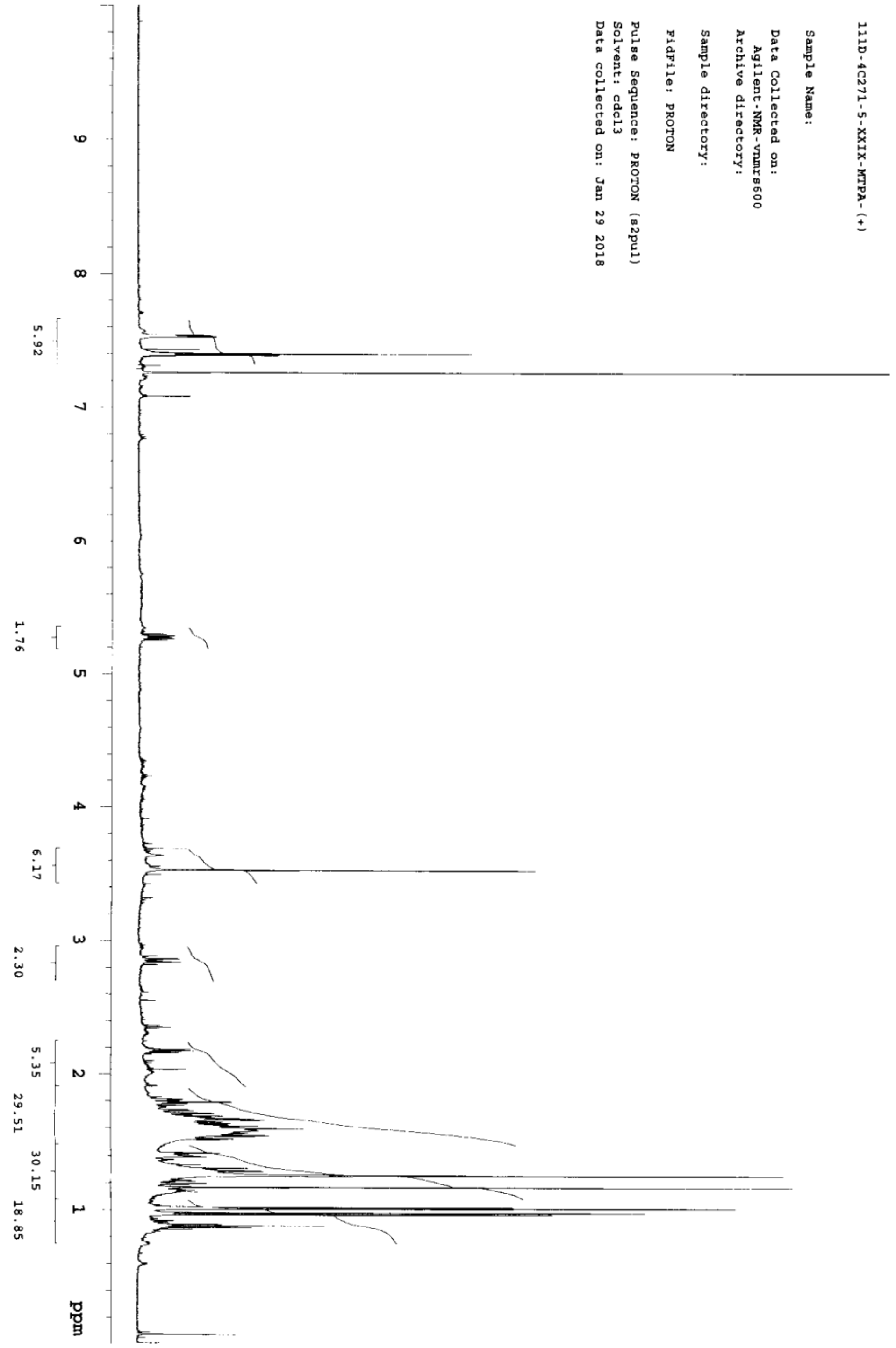

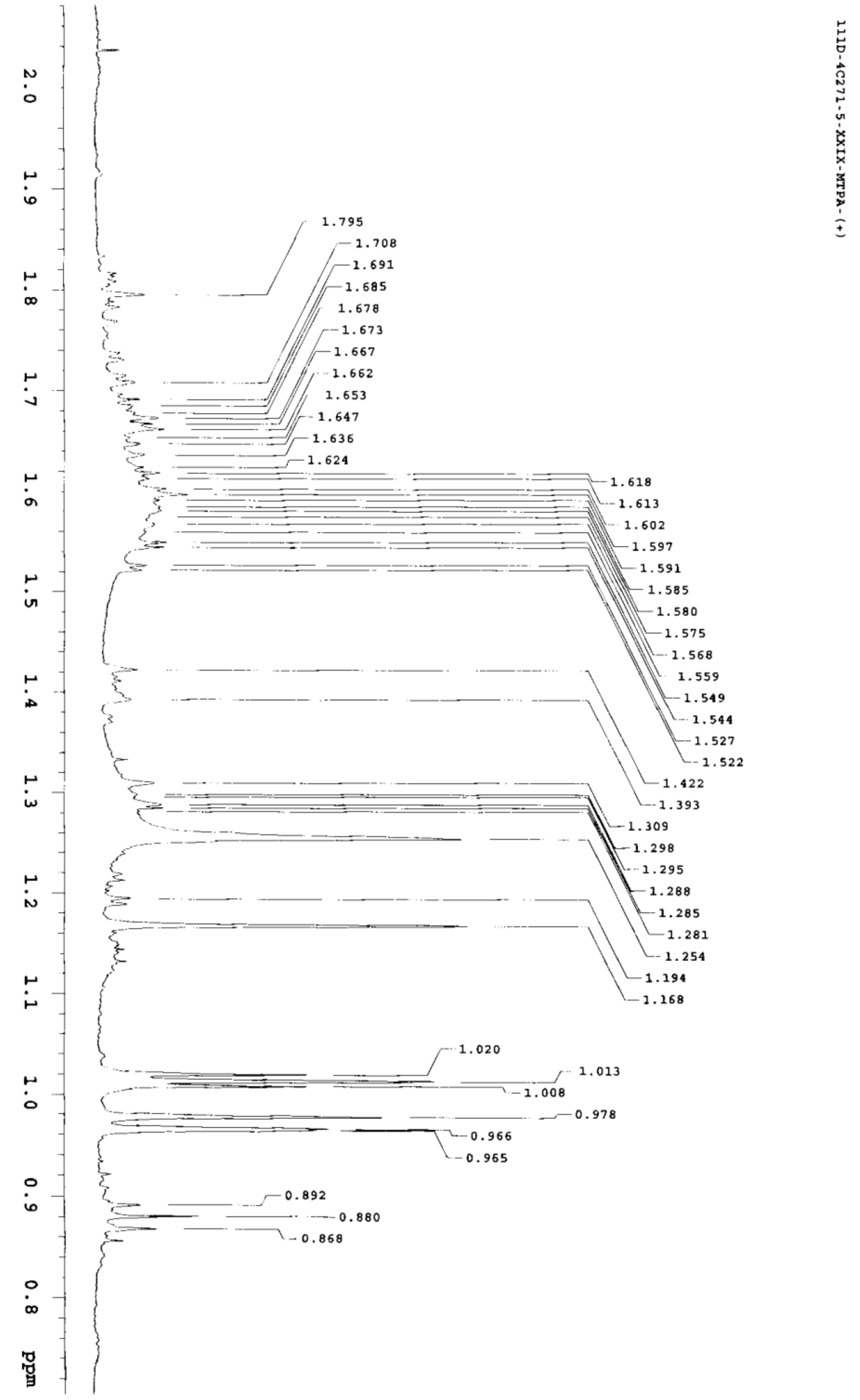
111D-4C271-5-XXIX-MTPA-(+)
1.795
1.708
1.691
1.685
1.678
1.673
1.667
1.662
1.653
1.647
1.636
1.624
1.618
1.613
1.602
1.597
1.591
1.585
1.580
1.575
1.568
1.559
1.549
1.544
1.527
1.522
1.422
1.393
1.309
1.298
1.295
1.288
1.285
1.281
1.254
1.194
1.168
1.020
1.013
1.008
0.978
0.966
0.965
0.892
0.880
0.868
2.0
1.9
1.8
1.7
1.6
1.5
1.4
1.3
1.2
1.1
1.0
0.9
0.8
ppm

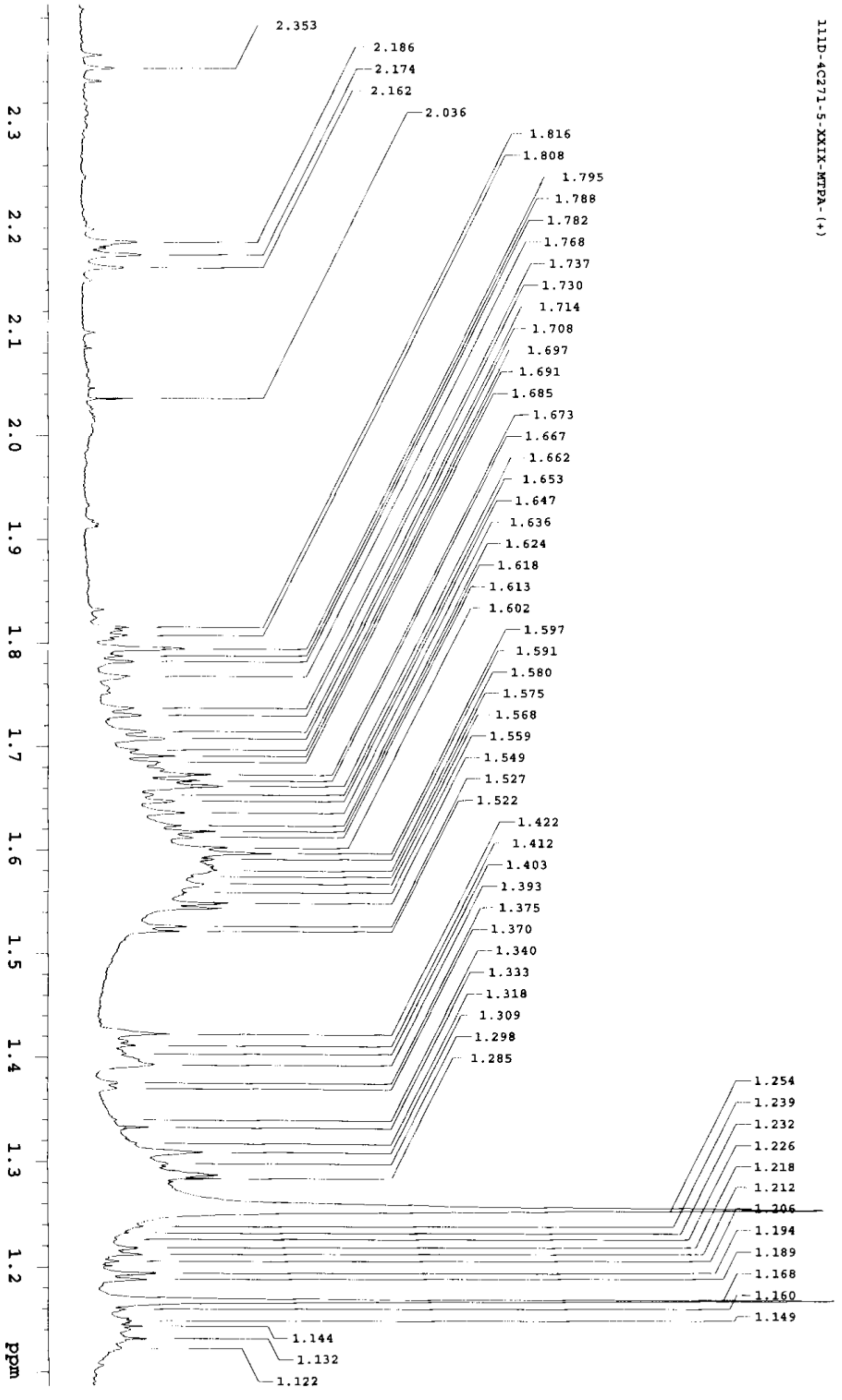
111D-4C271-5-XXIX-MTPA-(+)
2.3
2.2
2.1
2.0
1.9
1.8
1.7
1.6
1.5
1.4
1.3
1.2
ppm
2.353
2.186
2.174
2.162
2.036
1.816
1.808
1.795
1.788
1.782
1.768
1.737
1.730
1.714
1.708
1.697
1.691
1.685
1.673
1.667
1.662
1.653
1.647
1.636
1.624
1.618
1.613
1.602
1.597
1.591
1.580
1.575
1.568
1.559
1.549
1.527
1.522
1.422
1.412
1.403
1.393
1.375
1.370
1.340
1.333
1.318
1.309
1.298
1.285
1.254
1.239
1.232
1.226
1.218
1.212
1.206
1.194
1.189
1.168
1.160
1.149
1.144
1.132
1.122

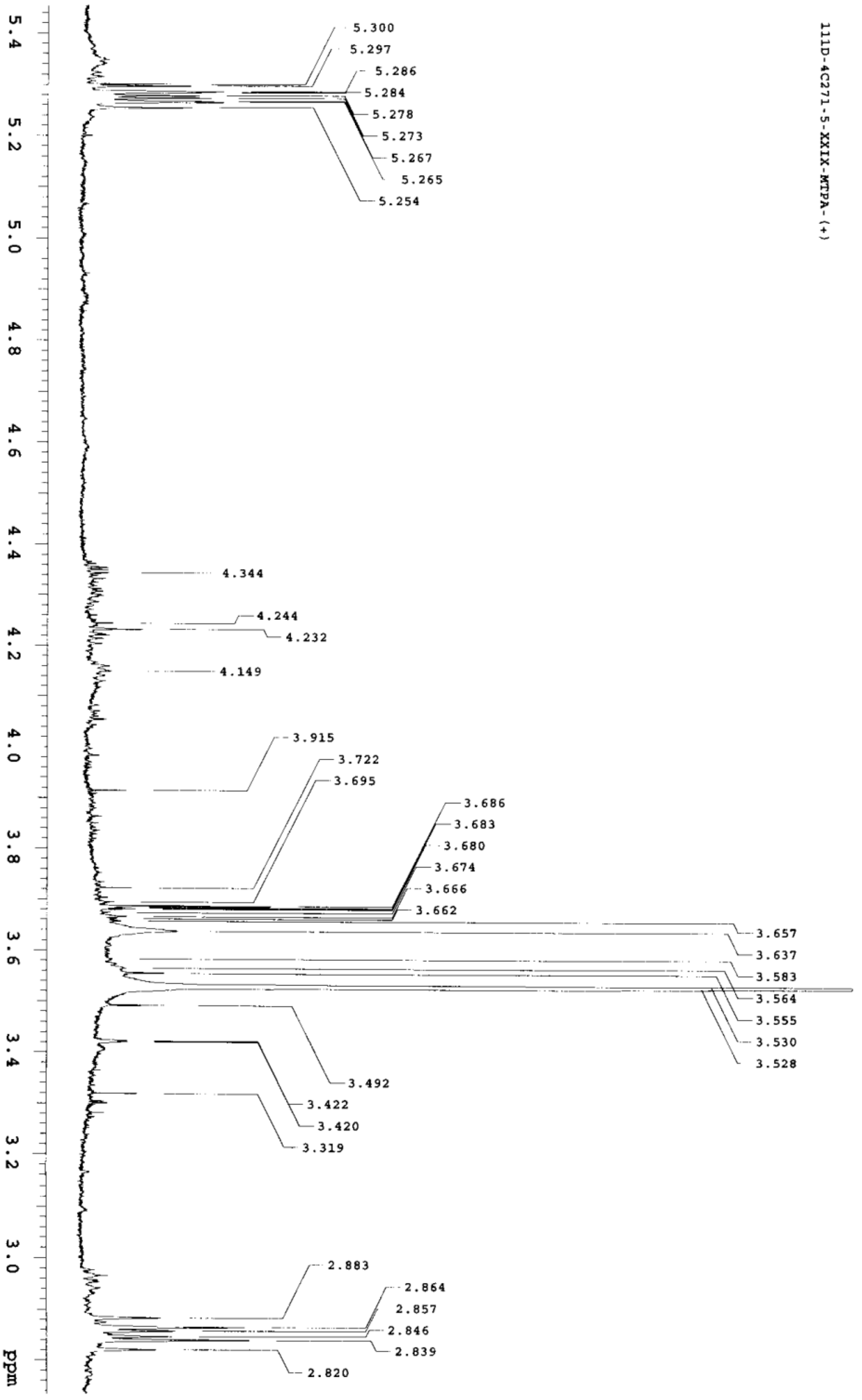
111D-4C271-5-XXIX-MTPA-(+)
5.300
5.297
5.286
5.284
5.278
5.273
5.267
5.265
5.254
4.344
4.244
4.232
4.149
3.915
3.722
3.695
3.686
3.683
3.680
3.674
3.666
3.662
3.657
3.637
3.583
3.564
3.555
3.530
3.528
3.492
3.422
3.420
3.319
2.883
2.864
2.857
2.846
2.839
2.820
5.4
5.2
5.0
4.8
4.6
4.4
4.2
4.0
3.8
3.6
3.4
3.2
3.0
ppm

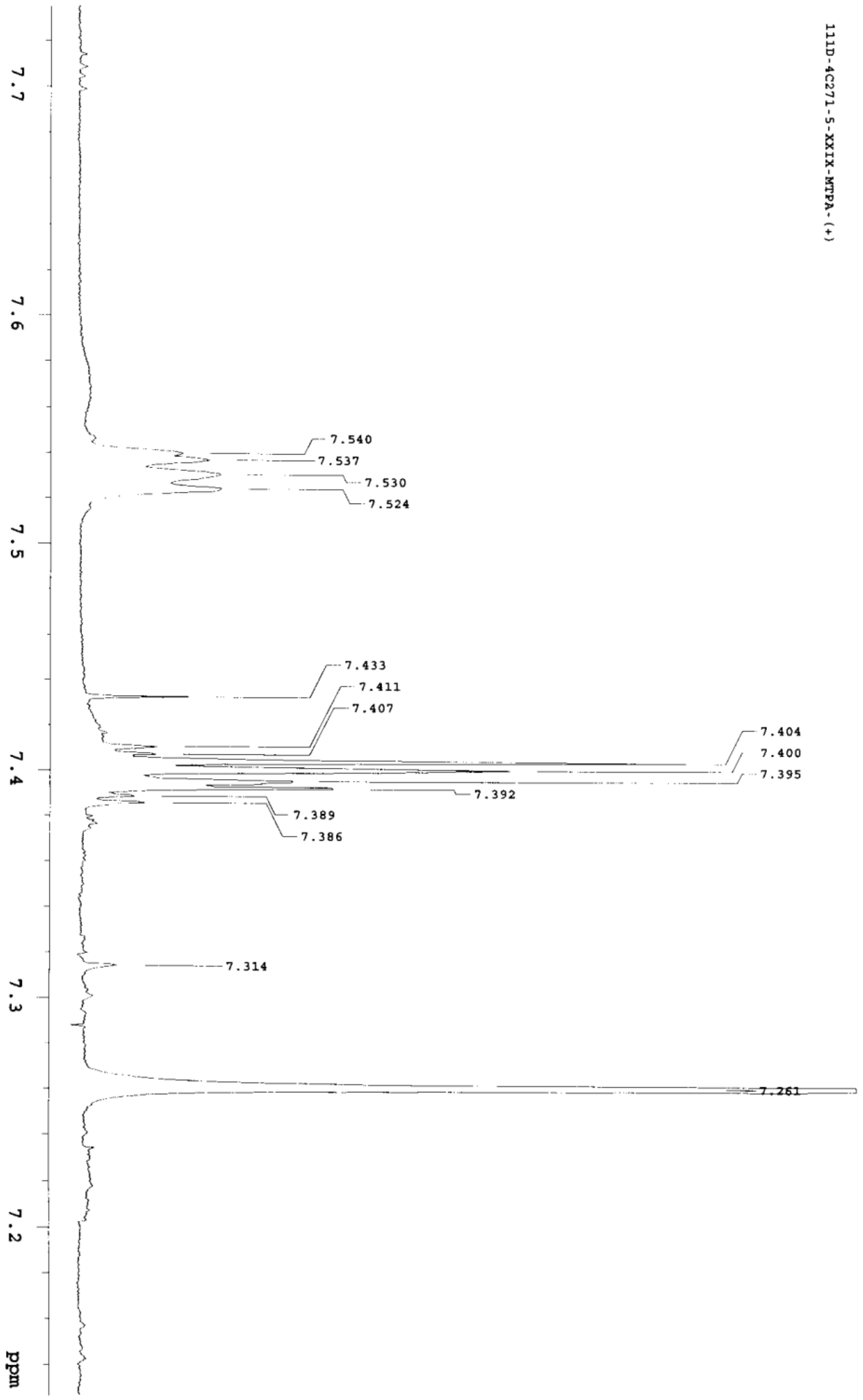
111D-4C271-5-XXIX-MTPA-(+)
7.540
7.537
7.530
7.524
7.433
7.411
7.407
7.404
7.400
7.395
7.392
7.389
7.386
7.314
7.261
7.7
7.6
7.5
7.4
7.3
7.2
ppm

**Figure S34 $^1$H-$^1$H COSY of 2b**

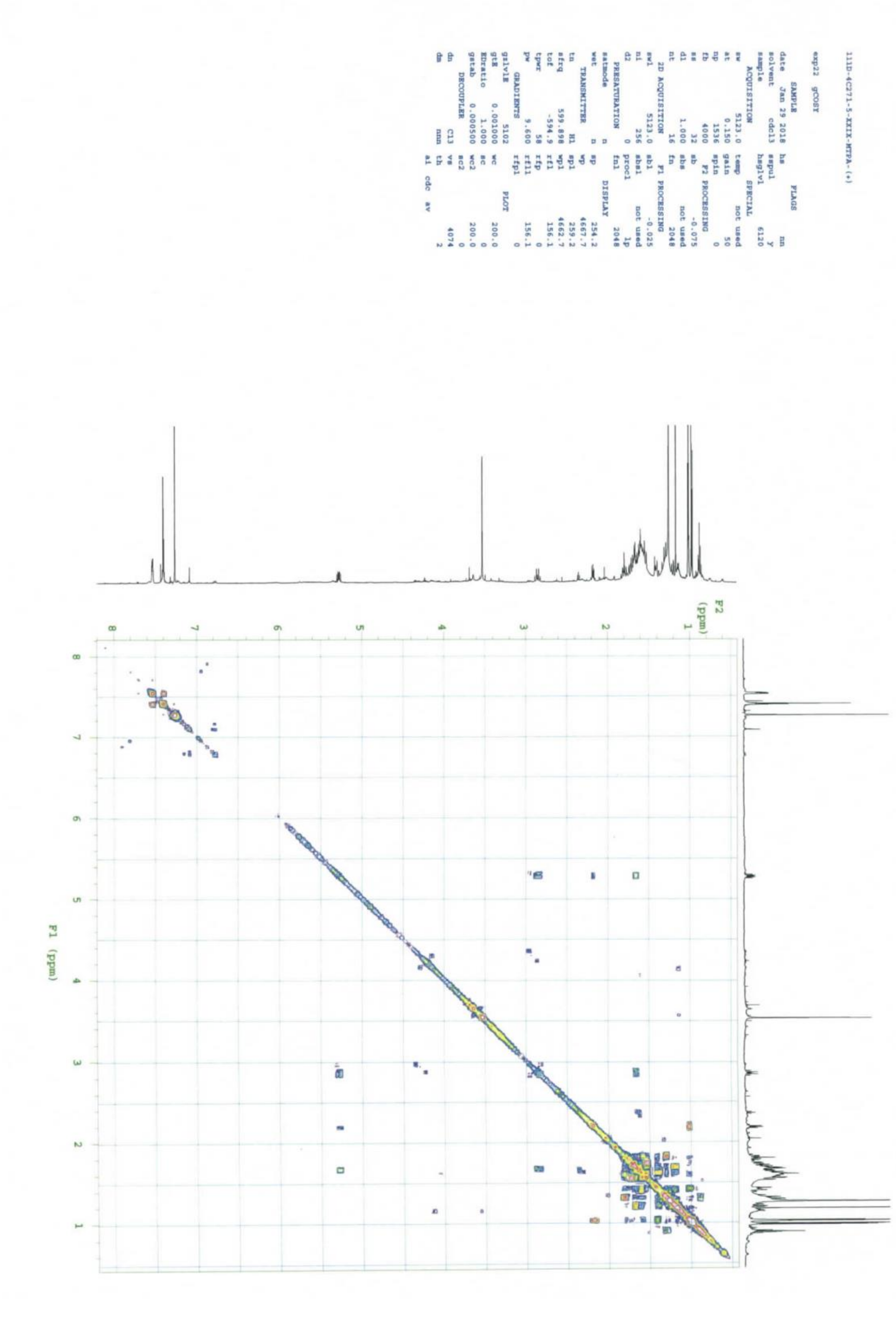

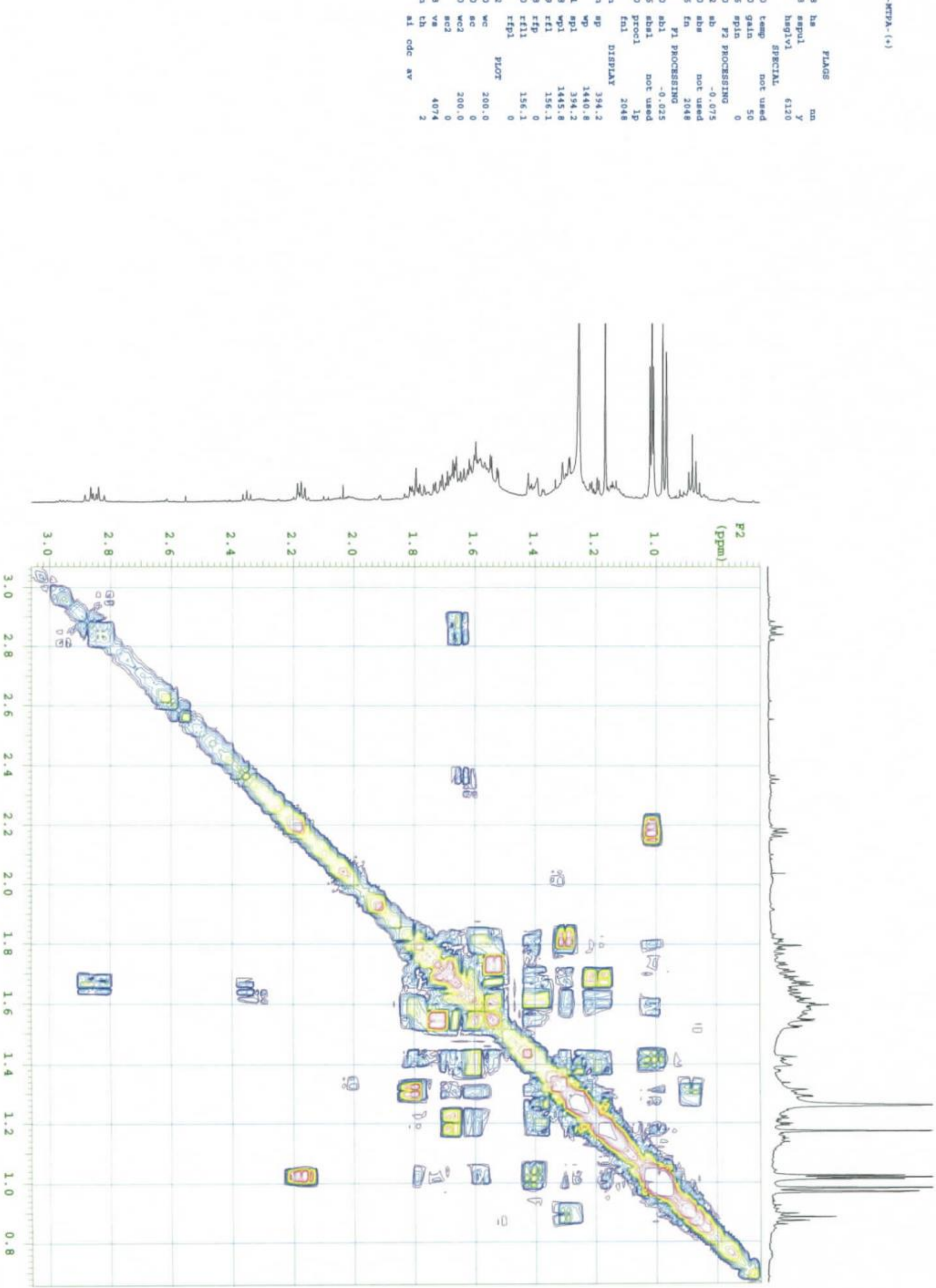

111D-4C271-5-XXIX-MTPA-(+)
exp22 gCOSY
SAMPLE
date Jan 29 2018
solvent cdcl3
sample
ACQUISITION
sw 5123.0
at 0.150
np 1536
fb 4000
ss 32
d1 1.000
nt 16
2D ACQUISITION
sw1 5123.0
ni 256
d2 0
PRESATURATION
satmode n
wet n
TRANSMITTER
tn H1
sfrq 599.898
tof -594.9
tpwr 58
pw 9.600
GRADIENTS
gzlvlE 5102
gtE 0.001000
EDratio 1.000
gstab 0.000500
DECOUPLER
dn C13
dm nnn
FLAGS
hs nn
sspul y
hsglvl 6120
SPECIAL
temp not used
gain 50
spin 0
F2 PROCESSING
sb -0.075
sbs not used
fn 2048
F1 PROCESSING
sb1 -0.025
sbs1 not used
proc1 lp
fn1 2048
DISPLAY
sp 394.2
wp 1440.8
sp1 394.2
wp1 1445.8
rfl 156.1
rfp 0
rfl1 156.1
rfp1 0
PLOT
wc 200.0
sc 0
wc2 200.0
sc2 0
vs 4074
th 2
ai cdc av
F2 (ppm)
F1 (ppm)

**Figure S35 NOESY of 2b**

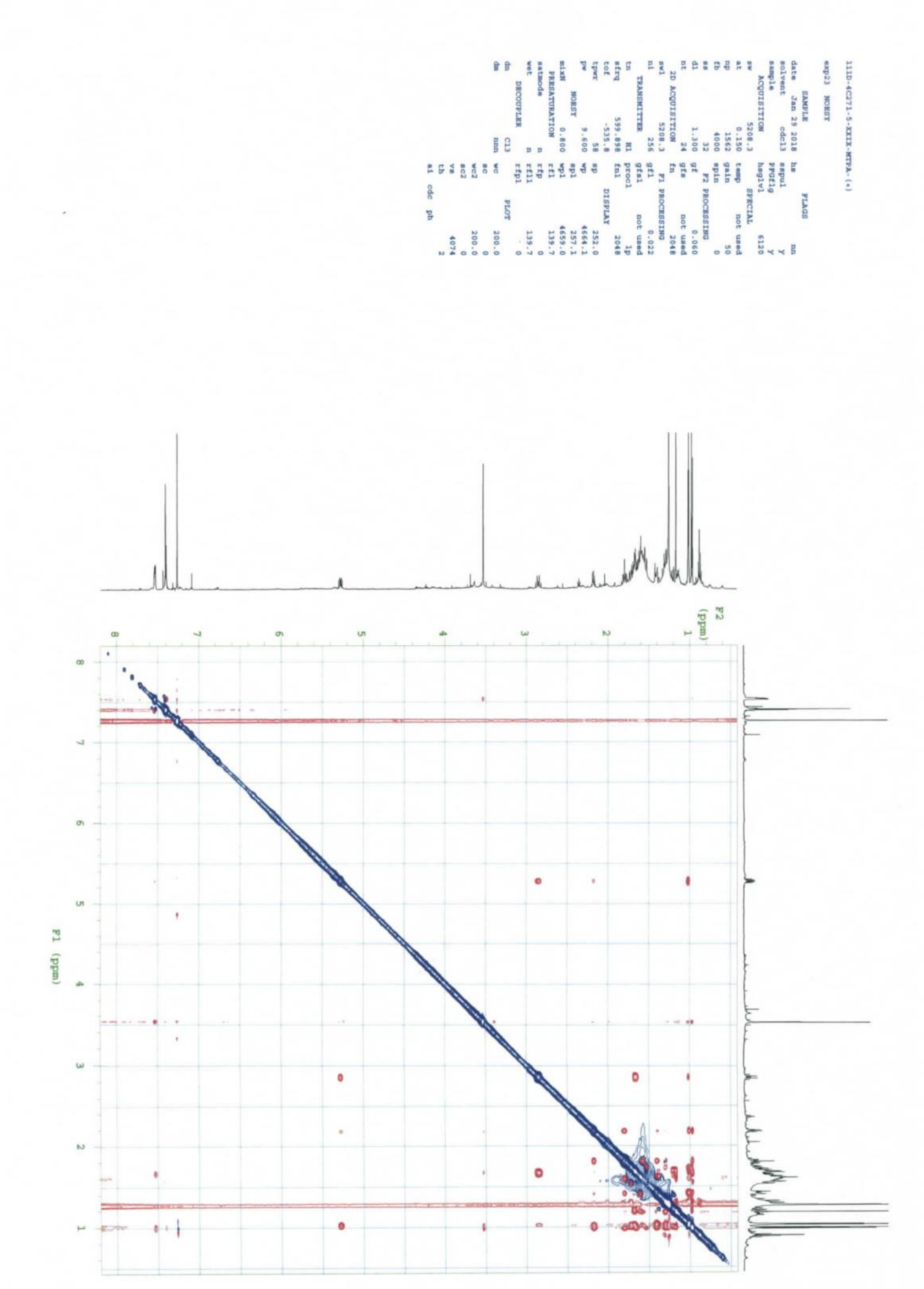

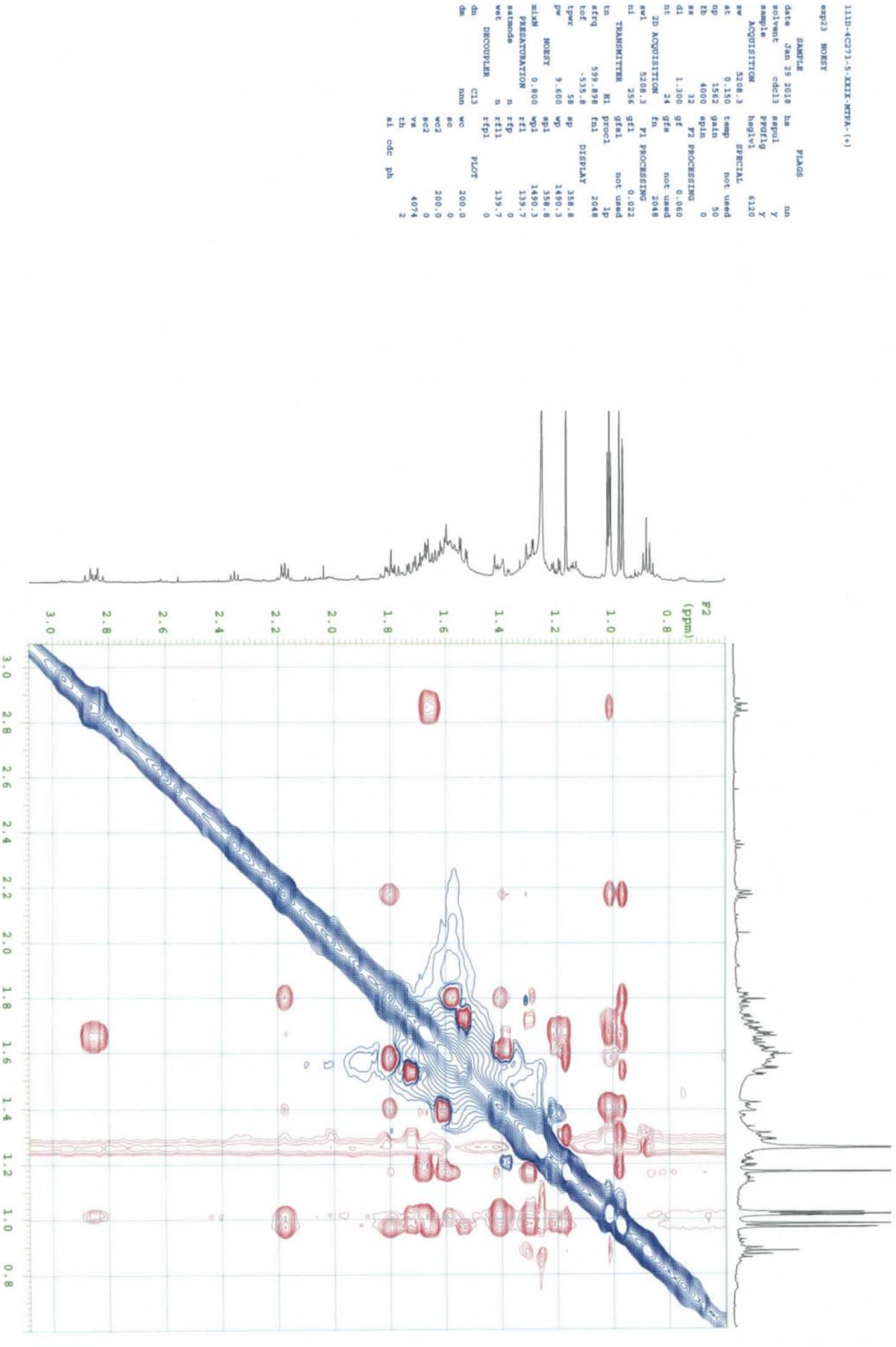
111D-4C271-5-XXIX-MTPA-(+)
exp23 NOESY
SAMPLE
date Jan 29 2018
solvent cdcl3
sample
ACQUISITION
sw 5208.3
at 0.150
np 1562
fb 4000
ss 32
d1 1.300
nt 24
2D ACQUISITION
sw1 5208.3
ni 256
TRANSMITTER
tn H1
sfrq 599.898
tof -535.8
tpwr 58
pw 9.600
NOESY
mixN 0.800
PRESATURATION
satmode n
wet n
DECOUPLER
dn C13
dm nnn
FLAGS
hs nn
sspul y
PFGflg y
hsglvl 6120
SPECIAL
temp not used
gain 50
spin 0
F2 PROCESSING
gf 0.060
gfs not used
fn 2048
F1 PROCESSING
gf1 0.022
gfs1 not used
proc1 lp
fn1 2048
DISPLAY
sp 358.8
wp 1490.3
sp1 358.8
wp1 1490.3
rfl 139.7
rfp 0
rfl1 139.7
rfp1 0
PLOT
wc 200.0
sc 0
wc2 200.0
sc2 0
vs 4074
th 2
ai cdc ph
F2 (ppm)
F1 (ppm)

**Figure S36 FABMS of 2b**

```
[ Elemental Composition ]                                          Page: 1
Data  : 1802047                      Date : 14-Feb-2018 18:26
Sample: 111D-4 C271-5 MTPA(+) XXIX
Note : Matrix; NBA+Na
Inlet : Direct                       Ion Mode : FAB+
RT : 5.34 min                        Scan#: (45,50)
Elements : C 32/28, H 45/35, F 5/0, O 10/0, Na 1/0
Mass Tolerance      : 20ppm, 1mmu if m/z > 50
Unsaturation (U.S.) : -1.0 - 30.0

Observed m/z Int%   Err[ppm / mmu]    U.S.  Composition
  545.2849   100.0     -1.1 /  -0.6    8.5  C 30 H 41 F 3 O 4 Na
```

[ Mass Spectrum ]
Data : 1802044 Date : 14-Feb-2018 17:42
Sample: 111D-4 C271-5 MTPA(+) XXIX
Note : Matrix; NBA+NaCl
Inlet : Direct Ion Mode : FAB+
Spectrum Type : Normal Ion [MF-Linear]
RT : 6.92 min Scan# : (52,62)-k((75,85))[k=1.0]
BP : m/z 189.0000 Int. : 157.06
Output m/z range : 20.0000 to 800.0000 Cut Level : 0.00 %

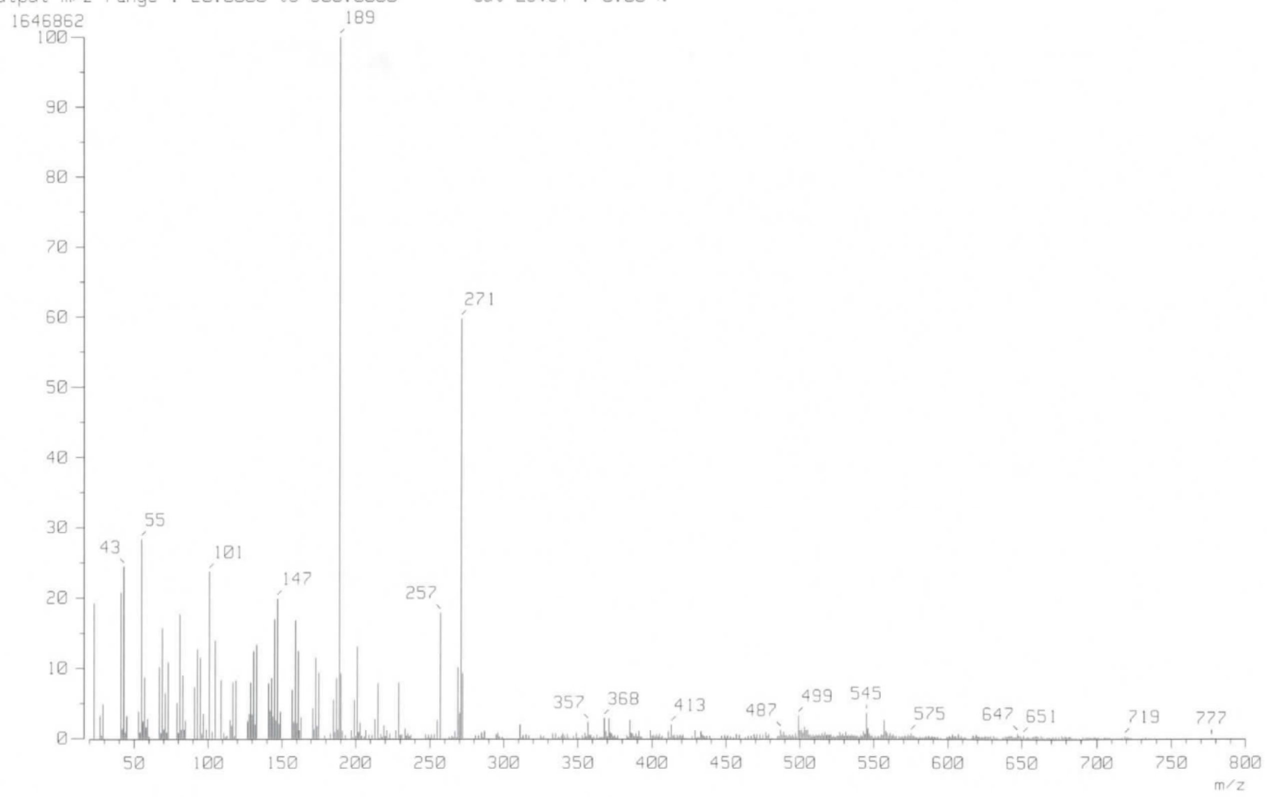

Supplement: Supplementary file 1 [file marinedrugs-17-00480-s001.zip › Supplementary files/SM-555194-Vol.2.pdf]
